# The Old Yellow Enzyme OfrA Fosters *Staphylococcus aureus* Survival *via* Affecting Thiol-Dependent Redox Homeostasis

**DOI:** 10.3389/fmicb.2022.888140

**Published:** 2022-05-17

**Authors:** Eslam S. Ibrahim, Knut Ohlsen

**Affiliations:** ^1^Institute of Molecular Infection Biology, University of Würzburg, Würzburg, Germany; ^2^Department of Microbiology and Immunology, Faculty of Pharmacy, Cairo University, Cairo, Egypt

**Keywords:** MRSA, blood, phagocytes, quinone, ROS, stress response, electrophilic stress

## Abstract

Old yellow enzymes (OYEs) are widely found in the bacterial, fungal, and plant kingdoms but absent in humans and have been used as biocatalysts for decades. However, OYEs’ physiological function in bacterial stress response and infection situations remained enigmatic. As a pathogen, the Gram-positive bacterium *Staphylococcus aureus* adapts to numerous stress conditions during pathogenesis. Here, we show that in *S. aureus* genome, two paralogous genes (*ofrA* and *ofrB*) encode for two OYEs. We conducted a bioinformatic analysis and found that *ofrA* is conserved among all publicly available representative staphylococcal genomes and some Firmicutes. Expression of *ofrA* is induced by electrophilic, oxidative, and hypochlorite stress in *S. aureus*. Furthermore, *ofrA* contributes to *S. aureus* survival against reactive electrophilic, oxygen, and chlorine species (RES, ROS, and RCS) *via* thiol-dependent redox homeostasis. At the host–pathogen interface, *S. aureus*Δ*ofrA* has defective survival in macrophages and whole human blood and decreased staphyloxanthin production. Overall, our results shed the light onto a novel stress response strategy in the important human pathogen *S. aureus*.

## Introduction

*Staphylococcus aureus* colonization is linked with an increased risk of infection (Krismer et al., 2017). *S. aureus* can cause minor (skin and soft tissue) and life-threatening infections (pneumonia, osteomyelitis, and bacteremia) (Tong et al., 2015). *S. aureus* is an ESKAPE pathogen, being increasingly resistant to the commonly prescribed antibiotics (Renner et al., 2017). Methicillin-resistant *S. aureus* increased prevalence leads to treatment failure due to multiple drug resistance (Fischbach and Walsh, 2009). Hence, we need a better understanding of the microbial factors impacting the host–pathogen interplay.

In cellular respiration, energy is produced *via* redox reactions in which electrons migrate through biomolecules to oxygen as final acceptor (Imlay, 2019). Reactive oxygen species (ROS) are generated either as an inevitable cost of oxidative respiration, a result of antibiotic exposure, or a consequence from the host’s immune response (Van Acker and Coenye, 2017; Imlay, 2019). Myeloperoxidase in activated macrophages and neutrophils generates reactive chlorine species (RCS). In particular, ROS and RCS are the main bacterial killing mechanisms in the phagolysosome (Klebanoff et al., 2013). *S. aureus* must cope with endogenous reactive electrophilic species (RES: menaquinones, siderophores, and methylglyoxal), reactive electrophilic species generated secondarily to ROS, and from host interaction (formaldehyde) (Groitl and Jakob, 2014; Chen et al., 2016). Therefore, *S. aureus* maintains defense systems against reactive oxygen, chlorine, and electrophilic species to quench their toxicities and repair the damaged biomolecules (Guerra et al., 2017; Reichmann et al., 2018; Linzner et al., 2020).

Electrophilic species have electron-deficient carbon centers such as α,β-unsaturated carbonyl compounds, quinones, and *N*-ethylmaleimide (NEM) (Farmer and Davoine, 2007). In *Escherichia coli*, NemA, a member of the old yellow enzyme (OYE) family, reduces NEM *in vitro* (Miura et al., 1997; Gray et al., 2013; Ozyamak et al., 2013). OYEs reduce activated C = C bonds in α,β-unsaturated carbonyl compounds *via* bound flavin mononucleotide cofactor and have broad substrate specificity (Williams and Bruce, 2002; Shi et al., 2020). OYEs are phylogenetically classified into the following: Class-I (from plants and bacteria), Class-II (from fungi), and Class-III (from bacteria) (Scholtissek et al., 2017). YqiG and YqjM are the two OYEs isolated from *Bacillus subtilis* and are orthologs to SAUSA300_0859 and SAUSA300_0322 in *S. aureus* USA300_FPR3757, respectively (Kitzing et al., 2005; Sheng et al., 2016). Recently, our group proved that SAUSA300_0859 exhibits a type-I nitroreductase activity against the DNA-binding antibacterial agent MT02 (El-Hossary et al., 2018). Despite the fact that many OYEs are useful biocatalysts, the physiological role of bacterial OYEs, to our knowledge, is still mysterious (Toogood et al., 2010).

Here, we show that OfrA has a role in preventing intoxication by RES, RCS, and ROS conditions and contributes to *S. aureus* survival in human blood and RAW 264.7 macrophage cell line. Furthermore, OfrA is associated with the carotenoid pigment (staphyloxanthin) production *via* upper mevalonate pathway.

## Materials and Methods

### Bacterial Strains, Growth Conditions, and Materials

A summary of the bacterial strains and oligos used in this study is listed in Supplementary Tables 1, 2, respectively.

B-medium is a modified LB medium suitable for staphylococci cultivation by adding 1 g/L potassium phosphate (Brückner, 2006). In cultivation steps, the ratio of the bacterial suspension to the total volume of the flask was less than or equal to 1:3 to ensure sufficient aeration.

RPMI medium (catalog number 72400021) was purchased from LIFE Technologies. Formaldehyde (FA), diamide, and NaOCl was bought from Fisher Scientific, MP Biomedicals, and Alfa Aesar, respectively. 4-Methylumbelliferyl-β-D-glucuronide hydrate (MUG), methylhydroquinone (MHQ), methylglyoxal (MG), H_2_O_2_, cumene hydroperoxide (CHP), and mevalonate were obtained from Sigma-Aldrich. Thiourea, N-acetylcysteine (NAC), and catalase was purchased from Carl Roth, Hölzel Diagnostika, and MP Biomedicals, respectively. Stressors were dissolved in sterilized Milli-Q water for β-galactosidase and survival assays.

### Bioinformatic and Phylogenetic Analyses

Completely assembled chromosomal sequences of *S. aureus* strains were retrieved from the NCBI website^1^ in May 2021. TBLASTN program from the standalone BLAST ncbi-blast-2.11.0+ used WP_000838037.1 as a query to search for possible proteins. Then, we used BLASTP program to identify the homology between the proteins retrieved *via* TBLASTN to WP_000838037.1. We consider 35% amino acids identity and protein length 375 ± 38 amino acids (10% deviation from WP_000838037.1) as a cutoff to limit OfrA-like proteins (Shi et al., 2020). Identical proteins were filtered out using SDDC program (Ibrahim et al., 2017).

Multiple sequence alignments were done using Clustal Omega^2^ with the default parameters. Phylogenetic trees were constructed using RAxML 8.0.0 software with the following setup (−f a −# autoMRE −m PROTGAMMAAUTO) (Stamatakis, 2014). Tree visualization and annotation were done using ggtree v2.0.1 (Yu, 2020).

### Chromosomal Manipulation of *Staphylococcus aureus* JE2

To construct EI011, we exchanged P*_*hla*_* in pKO10 with a 1-kb fragment upstream of *ofrA* (Ohlsen et al., 1997). The reporter plasmid was transformed into *E. coli* IM08B and then electroporated into *S. aureus* JE2 strain (Monk et al., 2015). We confirmed the single crossover event by sequencing of the amplified fragment using primer in the plasmid and another one upstream of the 1-kb fragment. To construct EI046 (JE2Δ*ofrA*), the allelic exchange in *S. aureus* JE2 strain was mediated by cloning the upstream and downstream fragments into pBASE6 shuttle vector (Geiger et al., 2012). After the double crossover with counter-selection, polymerase chain reaction (PCR) and sequencing were used to identify the mutant (Geiger et al., 2012).

### Minimum Inhibitory Concentration Assay

Overnight cultures in RPMI were diluted into final OD_600_ = 0.05 and incubated with serial dilutions of each compound. Minimum inhibitory concentration (MIC) is the minimum concentration that results in not more than (OD_600_ = 0.1) after 24-h incubation at 37°C with shaking at 200 rpm. OD_600_ were measured using Synergy H1 plate reader.

### β-Galactosidase Assay

The reporter strain was conditioned in RPMI for 24 h. We diluted the overnight culture 1:100 into fresh medium. In the overnight and the diluted culture, 10 mg/ml chloramphenicol was added as a final concentration. The resulting culture was grown in 37°C until transition from exponential phase to stationary phase (OD_600_ = 1.25 ± 0.05). 500 μl of the bacterial culture was supplemented with the stressor at the specified concentrations. After 2 h in 37°C with shaking at 200 rpm, samples were taken for analysis as indicated in the study of Vidal-Aroca et al. (2006).

### RNA Isolation and Reverse Transcription Quantitative Polymerase Chain Reaction

Overnight cultures grown in RPMI were diluted 1:100 to reach OD_600_ = 0.5. Samples were taken to represent the control before stress. Substances were added to the indicated concentrations and incubated for 15 min. After the incubation, the cultures were immediately put on ice to transfer to −80°C. RNA was isolated using RNAeasy Mini Kit following the manufacturer’s instructions. DNase I treatment was done using RapidOut DNA removal kit followed by cDNA synthesis *via* SuperScript IV Reverse Transcriptase utilizing random hexamer primers and *ofrA*-specific primer with non-staphylococcal tag (Supplementary Table 2). Quantitative PCR was performed with Biozym Blue S’Green qPCR Kit and *rho* and *rpoB* as the internal controls (Sihto et al., 2014).

### Bacterial Survival Assay

We diluted overnight cultures 1:100 in fresh RPMI followed by incubation at 37°C with shaking at 200 rpm until mid-logarithmic phase. OD_600_ were adjusted to be 0.4 after collecting the bacteria by centrifugation for 10 min at 4°C and 4,000 rpm. After adding the indicated concentration and incubation at 37°C for the indicated time interval, serial dilutions of the bacterial suspension were made followed by plating of 80 μl on LB agar using single plate-serial dilution spotting (SP-SDS) method (Thomas et al., 2015).

The exposure time to 1.5 mM NaOCl was 30 min, while we challenged the bacteria against 40 or 30 mM H_2_O_2_ for 1 h. MHQ and methylglyoxal were exposed for 3 h.

In H_2_O_2_ survival assay and after 1-h exposure, samples were centrifuged at 4,000 rpm for 10 min and then resuspended in sterile PBS supplemented with 10 mg/ml catalase. In NaOCl survival assay, the serial dilutions of the bacteria were made in sterile LB to quench the remaining NaOCl.

### Genomic DNA Isolation, Whole Genome Sequencing, and Variant Calling

Overnight cultures of *S. aureus* JE2 and EI046 (JE2Δ*ofrA*) strains were grown at 37°C with shaking at 200 rpm in B-medium. Genomic DNA was extracted using DNeasy Blood and Tissue Kit (from Qiagen) according to the manufacturer’s protocol modification for Gram-positive bacteria. Whole genome sequencing and variant calling were done by MicrobesNG. The raw sequenced reads are deposited in SRA database (BioProject ID: PRJNA812552).

### Macrophage Survival Assay

Similar protocol was applied as done in the study of Flannagan et al. (2018). RAW 264.1 macrophage cell line was prepared by passaging in RPMI medium supplemented with 10% FCS and Pen/Strep. Passages 12–15 were used in the bacterial survival assays. Bacteria grown in BHI to logarithmic phase were washed in sterile PBS, resuspended in RPMI, and added (∼5 × 10^7^ CFU) to ∼5 × 10^6^ RAW 264.1 cells in 24-well plates (MOI = 1:10).

Extracellular bacteria were killed by treatment with 150 μg/ml gentamicin in 1 h. We considered zero-time by adding fresh RPMI + 10% FCS medium. Samples (*n* = 5) were taken at time = 4, 24, and 48 h while the 4-h samples were set as the normalization factor as indicated (Fritsch et al., 2019). Bacterial counts were achieved as indicated above using SP-SDS method on LB agar.

### Whole Blood Killing

Venous blood specimens were collected from four healthy human blood donors (age: 21–32 years, gender: two women and two men) in tubes supplemented with anti-coagulant (1.6 mg/ml EDTA). Blood was kept at room temperature (RT) until use. We used a similar protocol to van der Maten et al. (2017) with some modifications.

Bacteria grown in BHI to logarithmic phase were washed two times with sterile PBS. Afterward, 30 μl of the bacterial suspension (2.2 × 10^7^ CFU/ml) was mixed with 100 μl of human blood (final concentration = 5 × 10^6^ CFU/ml). Saponin (final concentration = 1%), immediately or after 60-min incubation at 37°C with shaking, was added to blood–bacteria mixture for cell lysis. After incubation for 20 min at 4°C, viable bacterial cells were determined using SP-SDS method on LB agar in two technical replicates.

### RNA Isolation for RNA-Seq Experiment and Bioinformatic Analysis

Dilutions (1:100) of three independent overnight cultures of *S. aureus* JE2 and EI046 (JE2Δ*ofrA*) strains were grown at 37°C with shaking at 200 rpm in RPMI medium until OD_600_ = 0.5. We extracted total RNA using RNeasy Mini Kit (from Qiagen) as in the manufacturer’s manual. DNA digestion was done using RapidOut DNA Removal Kit. Negative amplification in PCR using 16S rDNA primers was taken as an evidence of successful DNase treatment. Evaluation of RNA quality, rRNA depletion, cDNA library generation, and sequencing were done by the Core Unit Systems Medicine Facility at the University Hospital Würzburg. Adaptor trimming was done using Cutadapt software. Trimmed reads were aligned to the reference genome (NC_007793). We used READemption pipeline for reads mapping, coverage calculations, gene quantification, and differential gene expression analysis (Förstner et al., 2014). We developed scripts for gene set enrichment analysis (GSEA) using clusterProfiler (Wu et al., 2021). Regulon analysis was done using self-written R scripts. RNA-seq data are available in NCBI’s Gene Expression Omnibus (GSE196683).

### Staphyloxanthin Assay

We diluted overnight cultures 1:100 in the respective medium (with supplementation if necessary) and allowed the bacterial growth in 37°C and shaking at 200 rpm for 16 h (stationary phase) (Sullivan and Rice, 2021). 2 ml of the bacteria was centrifuged at 16,000 rpm for 2 min and then washed in sterilized water. OD_600_ were recorded for normalization. 400 μl of methanol was added to the washed bacterial pellets and incubated at 55°C for 3 min. After centrifugation at 16,000 rpm for 2 min, 300 μl of the methanolic extract was added to 700 μl methanol. 200 μl of the solution was measured in three technical replicates at A_465_ with infinite 200Pro machine and methanol as a blank.

### Growth Inhibition Assay

Overnight cultures in RPMI were 1:100 diluted into fresh RPMI medium and incubated in 37°C with shaking at 200 rpm until OD_600_ = 0.5. Then, we diluted the bacteria down to ∼5 × 10^5^ cells and mixed with different concentration of streptonigrin (0–2 μg/ml). OD_600_ were measured using Synergy H1 plate reader.

### Statistical Analysis

Statistical analysis was done under R version 3.6.1 using rstatix R package version 0.6.0 and ggpubr R package version 0.4.0. Statistical tests were indicated in the corresponding figure legends. We considered statistical significance if *p* < 0.05.

## Results

### OfrA Is an Old Yellow Enzyme Flavin Oxidoreductase

**FIGURE 1:**
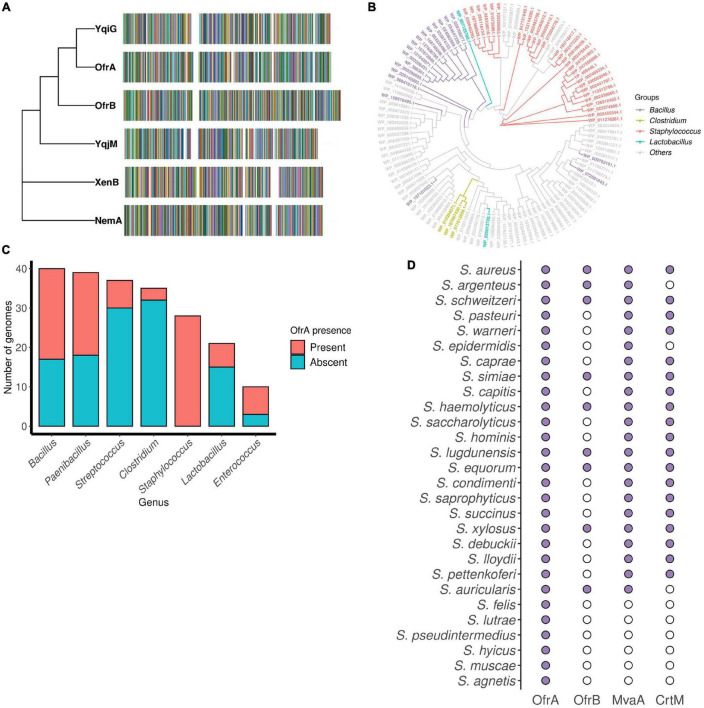
OfrA conservation in Firmicutes and staphylococci. **(A)** Phylogenetic analysis of OYE examples in Gram-positive *S. aureus* (OfrA, OfrB) and *B. subtilis* (YqiG, YqjM) compared to the Gram-negative *E. coli* (NemA) and *Pseudomonas fluorescens* (XenB) with distinctive multiple sequence alignment generated by Clustal Omega. **(B)** Maximum likelihood tree showing the evolutionary relationship of OfrA in different Firmicutes chromosomes. Multiple sequence alignment was utilized to build the phylogenetic tree using RAxML software and visualized with ggtree. **(C)** Bar chart shows the presence or absence of OfrA in seven Firmicutes genera (*Bacillus*, *Paenibacillus*, *Streptococcus*, *Clostridium*, *Staphylococcus*, *Lactobacillus*, and *Enterococcus*). Filtration criteria were based on 35% amino acid identity cutoff and protein length = 375 ± 38 amino acids, refer to section “Materials and Methods”. **(D)** OfrA conservation across the different staphylococci compared to the conservation of OfrB, MvaA (mevalonate pathway), and CrtM (staphyloxanthin biosynthesis) in the same genomes. Filled and unfilled circles indicate gene presence and absence, respectively. CrtM, squalene desaturase; MvaA, hydroxymethylglutaryl-CoA reductase; OfrA, **o**ld yellow enzyme **f**lavin oxido**r**eductase **A**; OfrB, **o**ld yellow enzyme **f**lavin oxido**r**eductase **B**.

Utilizing TBLASTN of WP_000838037.1 (SAUSA300_0859 gene product) against *S. aureus* USA300_FPR3757 genome, we found that OYEs are encoded from two paralogous genes (SAUSA300_0859 and SAUSA300_0322). We propose to name SAUSA300_0859 as **o**ld yellow enzyme **f**lavin oxido**r**eductase **A** (*ofrA*) and SAUSA300_0322 as *ofrB*. Upon NCBI’s CDD search, OfrA and OfrB contain “OYE_like_4_FMN” domain. OfrA and OfrB orthologs are conserved in *B. subtilis* as YqiG and YqjM, respectively (Figure 1A). Multiple sequence alignment shows that *E. coli*_NemA and *P. fluorescens*_XenB do not belong to the same class of Gram-positive OYEs (Figure 1A); rather, NemA and XenB belong to Class-I of classical OYEs whereas YqjM belongs to Class-III OYEs (Scholtissek et al., 2017). OfrA does not belong to any of the studied OYEs classes and represent a novel class of OYEs (Scholtissek et al., 2017). Therefore, we hypothesized that OfrA could play different roles in *S. aureus* than NemA in *E. coli*. Since *S. aureus* has a wide spectrum of genomic lineages, we were interested to study *ofrA* conservation in *S. aureus* strains and Firmicutes.

### OfrA Is Conserved in Staphylococci and Some Firmicutes

OfrA is encoded in all publicly available 749 chromosomes of *S. aureus* strains with 98–100% amino acid identities (Supplementary Table 3 and Supplementary Figure 1A). On genus level, OfrA is encoded from the 28 staphylococcal representative chromosomes. Staphylococcal OfrA orthologs cluster in three distinct clades (Figure 1B, Supplementary Figure 1B, and Supplementary Table 4). The phylogenetic tree illustrates that OYEs are not subjected to horizontal transfer; rather, they evolved within the encoding organism to adapt to certain function.

Although different OYEs are encoded in a number of representative Firmicutes’ chromosomes, OfrA-like orthologs are limited to only a few species (Figure 1C and Supplementary Figure 2). However, OfrB is not conserved across the different staphylococci (Figure 1D). In fact, BLASTP retrieved OfrA orthologs from the *ofrB*-minus genomes (such as *S. saprophyticus*, *S. hominis*, and *S. epidermidis*). From our analysis, we learned that some of the *ofrB*-minus genomes encode variants of the OYEs, other than OfrB, with varying lengths and/or other fused protein domains. Apparently, an earlier speciation event in a common ancestor resulted in OfrA and OfrB differences. Since OfrA is associated with MT02 resistance and is found conserved in staphylococci, we intended to understand the function of *ofrA* in the human pathogen *S. aureus* as an example of OYEs.

### Electrophilic Stress Conditions Induce *ofrA*

Previously, we showed that the bisquaternary bisnaphthalimide MT02 induces *ofrA* (El-Hossary et al., 2018). Thus, we hypothesized that compounds with an electron-deficient center (such as electrophilic stress generators) could similarly induce *ofrA*. To avoid the quenching activity of standard laboratory media (TSB and LB), we used RPMI as a well-defined medium, which, in addition, mimics the host environment (Meerwein et al., 2020).

To screen for important induction conditions, we constructed a reporter strain (EI011) that harbors a chromosomally encoded β-galactosidase from a promoter-less *lacZ* gene under the control of P*_*ofrA*_* (Supplementary Figure 3). Since β-galactosidase assay is protein-based, we chose 2-h exposure time to report for *ofrA* induction. We tested a range of RES conditions such as diamide, fosfomycin (Fosfo), formaldehyde (FA), methylglyoxal (MG), and MHQ at the MIC against EI011 to avoid false negative results from stressors’ toxicities in higher concentrations. The MIC concentration did not significantly affect the bacterial growth in the 2-h experimental time (Supplementary Figure 4).

**FIGURE 2:**
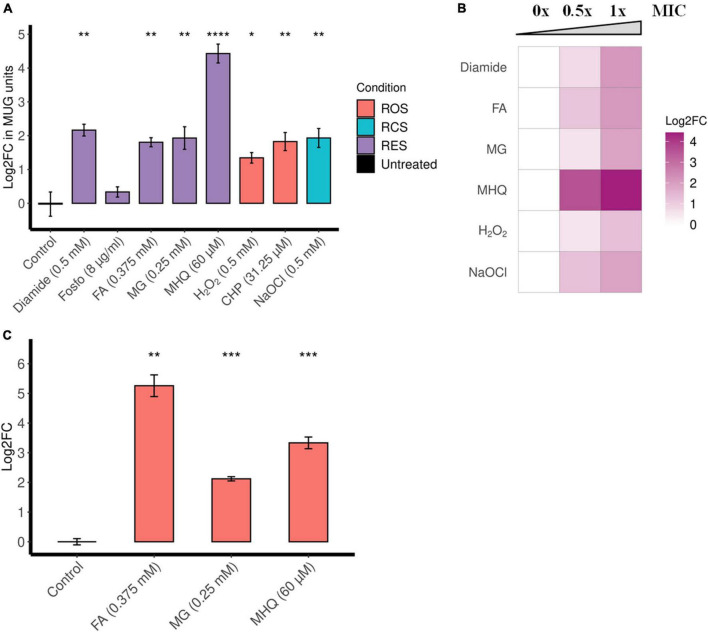
*ofrA* is induced in RES, ROS, and RCS in a dose-dependent manner. **(A)** *ofrA* induction conditions using the reporter strain EI011, which harbors a chromosomally encoded *lacZ* under P*_*ofrA*_* (Supplementary Figure 3). After 2-h incubation with shaking at 37°C, β-galactosidase assay was used to report *ofrA* transcriptional level. The corresponding concentrations were indicated in the graph. A total of four biological replicates were compared to untreated controls using unpaired two-tailed Student’s *t*-test. Error bars represent the standard error of the means. **(B)** Dose dependency of *ofrA* induction using β-galactosidase assays. The highest concentration is the minimum inhibitory concentration (1 × MIC), the intermediate concentration is 0.5 × MIC, and compared to control (no compounds were added = 0 × MIC). Log2FC was calculated as average from three biological replicates. **(C)** RT-qPCR shows *ofrA* induction in *S. aureus* JE2 background in agreement with the reporter system. JE2 strain was cultivated in RPMI until mid-logarithmic phase (OD_600_ = 0.5). Samples were taken before adding the compounds as a control. After adding the compounds, the bacterial pellets were collected after 15 min of incubation at 37°C with shaking. A total of three biological replicates were compared to untreated controls *via* unpaired two-tailed Student’s *t*-test. Error bars represent the standard error of the means. *p < 0.05; **p < 0.01; ***p < 0.001; ****p < 0.0001. CHP, cumene hydroperoxide; FA, formaldehyde; Fosfo, fosfomycin; MG, methylglyoxal; MHQ, methylhydroquinone; MIC, minimum inhibitory concentration; RCS, reactive chlorine species; RES, reactive electrophilic species; ROS, reactive oxygen species.

β-Galactosidase assays suggest that diamide, formaldehyde, methylglyoxal, and MHQ induce *ofrA* (Figure 2A). MHQ results in the highest upregulation (21-folds), while formaldehyde, methylglyoxal, and diamide result in approximately fourfold upregulation. However, there is no upregulation upon exposure to fosfomycin (Figure 2A). Furthermore, the β-galactosidase assays show a dose-dependent induction by diamide, formaldehyde, methylglyoxal, and MHQ (Figure 2B).

Since diamide is a non-specific disulfide-stress inducer, we were interested in the induction with toxic aldehydes (formaldehyde and methylglyoxal) and quinone-stress (MHQ). Reverse transcription quantitative polymerase chain reaction (RT-qPCR), comparing *ofrA* mRNA levels after 15-min exposure in *S. aureus* JE2 strain background, confirms the results obtained by the reporter strain (Figure 2C). After formaldehyde, methylglyoxal, and MHQ exposure for 15 min, there were 38-, 4-, and 10-fold upregulation in *ofrA*, respectively.

### OfrA Protects *Staphylococcus aureus* in Quinone Stress and Against Toxic Aldehydes

*Staphylococcus aureus* faces electrophilic stress in many natural niches including host–pathogen interface. Therefore, we were interested in elucidating the role of OfrA in *S. aureus* survival in electrophilic stress conditions in more detail. To address this, we used a marker-less deletion mutant in *S. aureus* JE2 (EI046 = Δ*ofrA*) as well as a complemented strain (EI047 = p*ofrA*) which harbors a plasmid-based expression of *ofrA* from its natural promoter. To assure the absence of secondary mutations that could affect any phenotype, we sequenced the whole genome of JE2 and Δ*ofrA*. The results show that there are no discriminating mutations in Δ*ofrA* compared to JE2 except for *ofrA* mutation.

**FIGURE 3:**
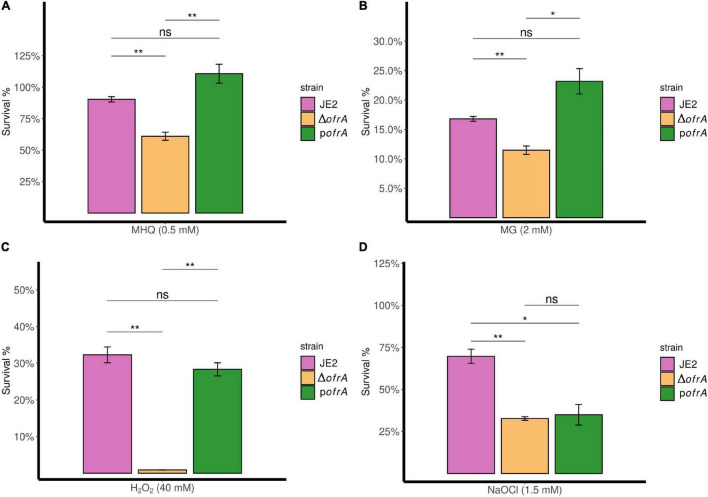
OfrA provides *S. aureus* resistance against quinone stress, toxic aldehydes, oxidative, and hypochlorite stresses. Bacterial survival assays in RES (MHQ and MG), ROS (H_2_O_2_), and RCS (NaOCl). The three strains; JE2, Δ*ofrA*, and p*ofrA* were allowed to grow until the logarithmic phase (OD_600_ = 0.4–0.6). Bacterial pellets were washed with sterile 1 × PBS, and the OD_600_ were adjusted to 0.4 in fresh RPMI. We added: **(A)** 0.5 mM MHQ for 3 h, **(B)** 2 mM MG for 3 h, **(C)** 40 mM H_2_O_2_ for 1 h, or **(D)** 1.5 mM NaOCl for 30 min. Samples were taken from the untreated control (for normalization) or with the stress conditions after the indicated time points for CFU determination using SP-SDS method on LB agar. Data represent four–five biological replicates. Error bars represent the standard error of the means. Statistical analysis was carried out using one-way ANOVA and pairwise *t*-test with Bonferroni *p*-value adjustment; ns, not significant; *p < 0.05; **p < 0.01. MG, methylglyoxal; MHQ, methylhydroquinone; RCS, reactive chlorine species; RES, reactive electrophilic species; ROS, reactive oxygen species.

In bacterial survival assays, we compared the survival of Δ*ofrA* vs. JE2 strain after 3-h exposure of 0.5 mM MHQ and 2 mM methylglyoxal compared to an untreated control. In quinone stress, JE2 strain survived 90%; however, Δ*ofrA* survived only 61%. p*ofrA* with restored *ofrA* expression complemented the survival defect phenotype (Figure 3A).

Δ*ofrA* exhibits a similar survival defect in methylglyoxal compared to the parental strain which we could restore in the complemented strain p*ofrA* (Figure 3B). We concluded that OfrA is important to mediate quinone-stress and toxic aldehydes and that OfrA is an important factor in *S. aureus* defense against electrophilic stress conditions.

### OfrA Affects the Survival of *Staphylococcus aureus* in Oxidative and Hypochlorite Stress

Since NemA was reported to be important in hypochlorite stress in *E. coli*, we also analyzed the role of OfrA in ROS and hypochlorite stress conditions. β-Galactosidase assays suggest that oxidative stress [H_2_O_2_ and cumene hydroperoxide (CHP)] and hypochlorite (NaOCl) stress induce *ofrA* (Figure 2A). Moreover, *ofrA* induction follows a dose response at different concentrations of H_2_O_2_ and NaOCl (Figure 2B).

Next, we analyzed the survival of the deletion mutant Δ*ofrA* vs. JE2 wild-type (WT) after 1-h exposure of 40 mM H_2_O_2_ compared to untreated control. The *ofrA* mutant strain had decreased survival in 40 mM H_2_O_2_ compared to WT (Figure 3C). Moreover, the complementation in p*ofrA* restored the WT phenotype indicating that OfrA enhances *S. aureus* survival in oxidative stress.

In 1.5 mM NaOCl, JE2 survived (70%) after 30 min compared to 33% survival of Δ*ofrA* denoting OfrA importance in *S. aureus* survival against NaOCl (Figure 3D). However, p*ofrA* failed to complement the mutant phenotype. We hypothesized that complementation with a high-copy number plasmid could result in overconsumption of cellular resources and therefore a decreased resistance against NaOCl taking in consideration the devastating non-specific effects of HOCl (da Cruz Nizer et al., 2020). From the whole genome sequencing results, there are no secondary mutations that could affect NaOCl survival phenotype. In addition, NaOCl results in *ofrA* induction (Figure 2A). Hence, we concluded that OfrA is also an important factor in *S. aureus* defense against ROS and hypochlorite stress conditions. To assure that there are no effects of strain growth behavior on the survival phenotypes, we observed the growth kinetics of the logarithmic phase cells of the three strains in RPMI medium (Supplementary Figure 5). Similar growth kinetics suggested that the growth behavior did not contribute to any of the measured survival phenotypes.

### *Staphylococcus aureus* USA300 JE2Δ*ofrA* Shows Decreased Fitness at the Host–Pathogen Interface by Survival Defect in Murine Macrophages RAW 264.7 Cell Line and Whole Human Blood

Macrophages produce reactive oxygen, chlorine, and electrophilic species as the killing factors against internalized *S. aureus* (Moldovan and Fraunholz, 2019). Since OfrA is important in survival in these stress conditions, we wondered whether *ofrA* mutation results in defective macrophage survival.

**FIGURE 4:**
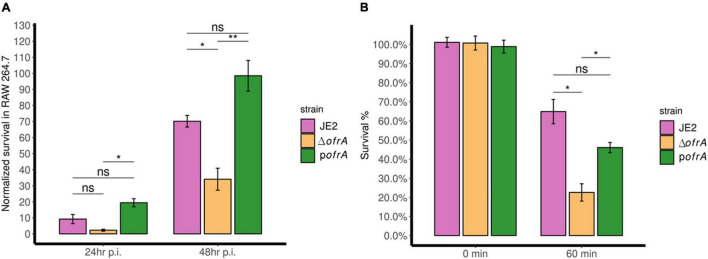
OfrA promotes *S. aureus* fitness at the host-pathogen interface by enhancing survival in RAW 264.7 macrophages and whole human blood. **(A)** Macrophage survival assay. JE2, Δ*ofrA*, and p*ofrA* were added to RAW 264.7 macrophage cell lines in 1:10 MOI. After 1 h of infection, gentamicin (150 μg/ml) was used to kill extracellular bacteria for 1 h. Fresh RPMI + 10% FCS was added (*t* = 0). At (*t* = 4 h), viable intracellular bacteria were determined and the CFU/ml was used as a normalization factor. Samples were taken at (*t* = 24 and 48 h). The assay was repeated for three independent experiments. Data represent five biological replicates from one of the three experiments. **(B)** Whole human blood killing assay. A total of 5 × 10^6^ CFU/ml of each strain were incubated in whole human blood for 60 min at 37°C with continuous shaking. The number of viable bacteria was enumerated after serial dilutions in sterile 1 × PBS using SP-SDS method on LB agar and normalized to the viable cells without incubation. The experiment was repeated in blood taken from four different blood donors. Data represent four biological replicates from one of the four experiments. Error bars represent the standard error of the means. Statistical analysis was carried out using one-way ANOVA and pairwise *t*-test with Bonferroni correction *p*-value adjustment; ns, not significant; *p < 0.05; **p < 0.01.

After 24 h, Δ*ofrA* survival was reduced in RAW 264.7 macrophages compared to JE2 but this was not statistically significant. However, the difference became significant after 48 h. After 48 h, Δ*ofrA* survived significantly (∼50%) less than JE2 in RAW 264.7 cell line (Figure 4A). In the complemented strain, the difference between p*ofrA* and Δ*ofrA* was statistically significant even after 24 h. The bigger difference could be explained by *ofrA* dosage effect from the high-copy number plasmid utilized in the complementation. We concluded that OfrA affects the bacterial fitness by enhancing *S. aureus* JE2 survival in macrophages.

The bacteria–immune response interaction in human blood determines the fatality of *S. aureus*-mediated bacteremia. We wondered whether OfrA contributes to *S. aureus* JE2 virulence *via* promoting survival in human blood. After 1 h of incubation with whole human blood, Δ*ofrA* survives (∼23%) compared to the WT (∼65%) (Figure 4B). The complementation in p*ofrA* restores the survivability of the mutant back to ∼46% (Figure 4B). In conclusion, *ofrA* contributes to *S. aureus* survival in whole human blood.

### *ofrA* Deletion Promotes Transcriptional Changes in Some Redox and Stress-Related Genes

To understand *ofrA* function in *S. aureus*, we compared the transcriptome of Δ*ofrA* vs. JE2 in mid-logarithmic phase in RPMI. Through RNA-seq experiment, we found that the *ofrA* mutant had decreased RNA abundances corresponding to 93 genes and increased RNA abundances corresponding to 95 genes (Supplementary Table 5). Several redox-related (SAUSA300_0339, SAUSA300_0340, SAUSA300_0212, SAUSA300_0213, *ypdA*, and *cymR*) and stress-related genes (*csbD*, *clpB*, *sigB*, and *rsbW*) are deregulated. Using regulon analysis and GSEA, we observed the following: (1) one-carbon metabolism is inhibited in Δ*ofrA* indicating an unbalanced redox status (Shetty and Varshney, 2021), and (2) the carotenoid biosynthesis (*crtOPQMN*) is suppressed in the mutant (Supplementary Table 6).

To validate the results of RNA-seq analysis, we performed RT-qPCR to quantify the mRNA abundances of *crtM*, *acuA*, and *rocD* genes. RT-qPCR confirmed the results obtained by the RNA-seq analysis. In RT-qPCR, log_2_ (fold change) of *crtM* expression is −0.7 ± 0.1 in Δ*ofrA* compared to JE2 (Supplementary Figure 6). Moreover, log_2_ (fold change) of *acuA* and *rocD* expression is −0.6 ± 0.2 and 2.9 ± 0.2, respectively (Supplementary Figure 6).

### Suppressed Staphyloxanthin Production in Δ*ofrA* Is Glucose-Independent But Mevalonate-Dependent

**FIGURE 5:**
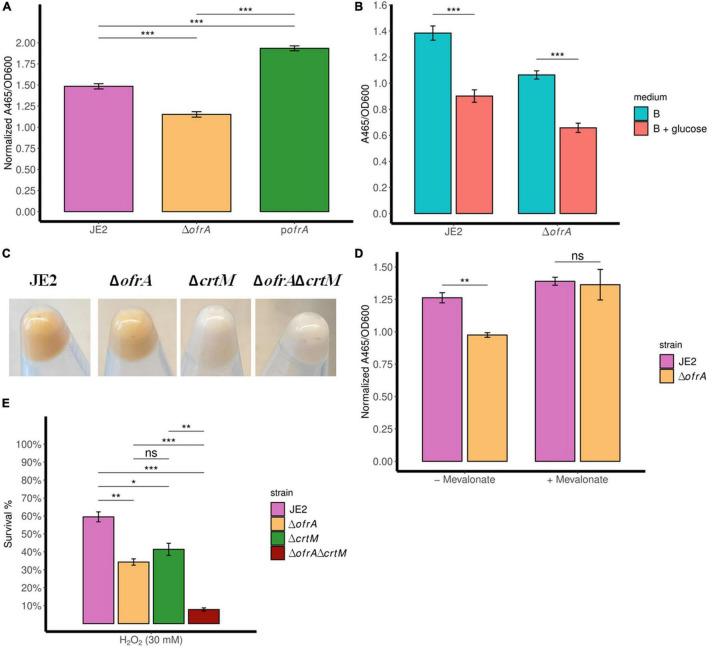
*ofrA* mutation decreases STX production *via* the upper mevalonate pathway but cannot solely explain ROS hypersensitivity. Staphyloxanthin assay showing STX levels in TSB medium **(A,C)**, B-medium **(B)**, and RPMI **(D)**. The strains were grown in overnight culture in the respective medium without any supplementation. Then, we diluted the overnight cultures 1:100 in fresh medium without or with supplementation; 0.5% glucose **(B)** or 1 mM mevalonate **(D)**. After 24 h, the bacteria were collected and washed with sterile water. OD_600_ were recorded for normalization. STX was extracted using methanol (refer to section “Materials and Methods”). A_465_ were used for measuring the extracted STX. Error bars represent the standard error of the means **(A,D)** and standard deviation **(B)** of four biological replicates. Statistical analysis was carried out using unpaired two-tailed Student’s *t*-test **(B,D)** or one-way ANOVA and pairwise *t*-test with Bonferroni *p*-value adjustment **(A)**; ns, not significant; *p < 0.05; **p < 0.01; ***p < 0.001. **(E)** Bacterial survival assays showing *crtM* mutation additive effect to *ofrA* mutation in ROS hypersensitivity. The strains were grown in overnight culture in RPMI medium. We diluted the overnight cultures 1:100 in fresh RPMI until mid-logarithmic phase. Cells were harvested by centrifugation and washed with sterile PBS. OD_600_ were adjusted to 0.4. Bacteria were challenged with 30 mM H_2_O_2_. After 1 h of exposure to 30 mM H_2_O_2_, viable cells were diluted in PBS after catalase treatment for residual H_2_O_2_. Samples were taken from the untreated control (for normalization) or with the stress condition after 1 h for CFU determination using SP-SDS method on LB agar. STX, staphyloxanthin.

The carotenoid pigment (staphyloxanthin) production is mediated *via* the *crtOPQMN* operon (Götz, 2005). Staphyloxanthin (STX) is a virulence factor that affects the survival of *S. aureus* against oxidative stress and human neutrophils, so we were interested in quantifying STX levels in the *ofrA* mutant (Clauditz et al., 2006). Indeed, STX is decreased in Δ*ofrA* compared to JE2 and p*ofrA* (Figure 5A).

Acetyl-CoA is the key input of mevalonate pathway to produce farnesyl pyrophosphate (FPP), which enters the *crtOPQMN* pathway (Pelz et al., 2005). STX was previously shown to be decreased with glucose due to intracellular acetyl-CoA loss (Tiwari et al., 2018). To test the acetyl-CoA-dependency of STX phenotype in the *ofrA* mutant, we measured STX level in B-medium (contains no glycolytic substrates) ± 0.5% glucose.

As expected, glucose decreased STX levels in WT (Figure 5B). However, the intracellular acetyl-CoA loss did not affect the ratio between Δ*ofrA* and JE2 in STX production (Figure 5B). This result suggests that Δ*ofrA* does not have decreased STX production *via* change in acetyl-CoA concentration. Conversely, the loss of *crtM* in JE2 and Δ*ofrA*, transduced from strain Newman (Clauditz et al., 2006), resulted in the disappearance of the *ofrA*-dependent phenotype, and both strains become white (Figure 5C; Reichert et al., 2018). Therefore, we concluded that *ofrA* mutation could affect the mevalonate pathway.

The mevalonate pathway is classified into upper (*mvaS*, *mvaA*) and lower (*mvaK1*, *mvaK2*, and *mvaD*) mevalonate pathways (Reichert et al., 2018). The output of the upper mevalonate pathway is the mevalonate itself. So, we were interested to understand the dependency of *ofrA*-mediated STX phenotype on the presence of mevalonate.

We compared the STX production ±1 mM mevalonate in RPMI medium. The presence of mevalonate results in the disappearance of *ofrA*-mediated phenotype in Δ*ofrA* compared to JE2 (Figure 5D). Therefore, we deduced that the *ofrA* mutant has decreased STX production *via* the upper mevalonate pathway in *S. aureus*.

### Lower Staphyloxanthin Generation Cannot Solely Explain Reactive Oxygen Species Hypersensitivity in Δ*ofrA*

To understand whether ROS hypersensitivity is linked to decreased STX, we challenged JE2Δ*crtM* and JE2Δ*crtM*Δ*ofrA* strains against H_2_O_2_ in the survival assay. As expected, *crtM* and *ofrA* mutations in JE2 resulted in decreased survival in ROS (Figure 5E). If low STX production is responsible for ROS-mediated killing, the double deletion mutants shall behave as Δ*crtM* and Δ*ofrA*. Contrary to this hypothesis, the double mutation in both genes, JE2Δ*crtM*Δ*ofrA*, causes H_2_O_2_ hypersensitivity and more killing in 30 mM H_2_O_2_ (Figure 5E). Thus, *crtM* and *ofrA* are important in ROS survival but independent of each other.

### OfrA Contributes to Reactive Oxygen Species Tolerance Through Affecting Thiol-Dependent Redox Homeostasis

From RNA-seq analysis, we know that *ofrA* mutation does not result in upregulation of *sodA*, *sodM*, *katA*, peroxidases, and *hmp*, which indicates that intracellular levels of O_2_^–^ and H_2_O_2_ are within the WT levels (Supplementary Table 5). Therefore, *ofrA*-dependent ROS hypersensitivity is downstream to H_2_O_2_ production.

**FIGURE 6:**
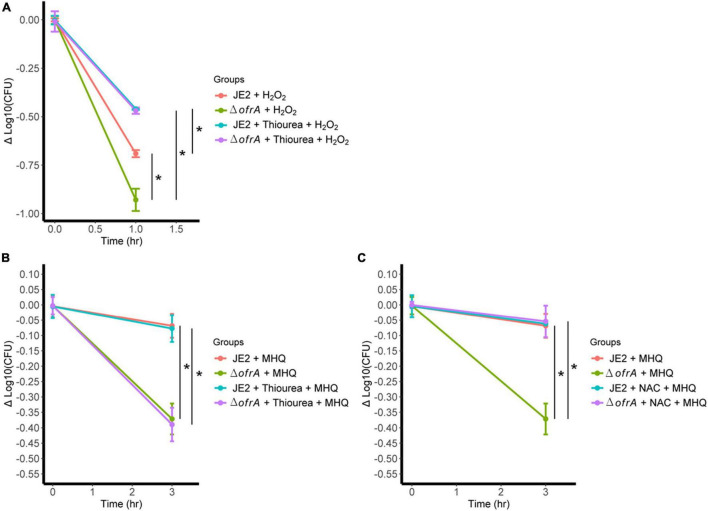
*ofrA* mutation increases ROS-mediated killing *via* disturbing thiol-dependent redox homeostasis. **(A)** Bacterial survival assay in 40 mM H_2_O_2_ with or without 120 mM thiourea. The strains were grown in overnight culture in RPMI medium. We diluted the overnight cultures 1:100 in fresh RPMI until mid-logarithmic phase. Cells were harvested by centrifugation and washed with sterile PBS. OD_600_ were adjusted to 0.4. Bacteria were challenged with H_2_O_2_ with or without 120 mM thiourea. Bacterial survival assay in 0.5 mM MHQ with or without 120 mM thiourea **(B)** or 1.25 mM NAC **(C)**. Data represent four biological replicates. Error bars represent the standard error of the means. Statistical analysis was carried out using one-way ANOVA and pairwise *t*-test with Bonferroni *p*-value adjustment; *p < 0.05. MHQ, methylhydroquinone; NAC, *N*-acetyl cysteine.

The only plausible explanation of ROS hypersensitivity we had is that *ofrA* contributes to the repair mechanism of thiol-oxidation caused by H_2_O_2_. This notion is supported by the fact that *ofrA* is generally induced with electrophilic, hypochlorite, and oxidative stress. Thiourea scavenges the hydroxyl radical that should decrease the H_2_O_2_-mediated killing (Wasil et al., 1987). As expected, the survival of Δ*ofrA* was lower than JE2 strain in H_2_O_2_ (Figure 6A). Addition of 120 mM thiourea resulted in increased *S. aureus* JE2 survival and Δ*ofrA* up to a similar level (Figure 6A). Therefore, we concluded that *ofrA* contributes to oxidative stress tolerance *via* a repair mechanism downstream to H_2_O_2_ but upstream to hydroxyl radical-mediated lethality.

Since MHQ is the highest induction condition (Figure 2A) and the hydroxyl radical is the main killing mechanism after H_2_O_2_ challenge (Figure 6A), we hypothesized that the survival defect of the mutant in ROS is secondary to disruption of thiol-dependent homeostasis upon ROS challenging.

In *S. aureus*, MHQ imposes oxidative and electrophilic stress (Fritsch et al., 2019). To test our hypothesis, we conducted MHQ survival assay ± *N*-acetyl cysteine (NAC). NAC supports the thiol-dependent redox homeostasis that acts as both reactive oxygen and electrophilic species scavenger (Pedre et al., 2021), and thiourea as ROS scavenger *via* thiol-independent mechanism. If our hypothesis was correct, thiourea would not be able to quench the electrophilic stress. 120 mM thiourea does not abolish MHQ toxicity in *ofrA* mutation; however, 1.25 mM NAC does (Figures 6B,C). We, therefore, concluded that *ofrA* plays a role in the thiol-dependent redox homeostasis, which affects the survival in oxidative, electrophilic, and hypochlorite stress, and that is an essential function during infection inside macrophages, and in human blood.

## Discussion

Old yellow enzyme family proteins are widely distributed in the bacterial kingdom with yet-to-be explored functions. In this study, we aimed at identifying the physiological role of the staphylococcal conserved OYE OfrA in *S. aureus*. We learnt that OfrA is an important resistance factor against reactive species (RES, RCS, and ROS). Moreover, the virulence of *S. aureus* is decreased by compromised survival in murine RAW 264.7 macrophages and whole human blood after *ofrA* deletion.

We noticed that *ofrA* mRNA levels are stable in different media and growth phases and were only slightly (approximately two to fourfolds) upregulated under all tested stress conditions except for MHQ induction. One reason for that behavior could be the promiscuity known to OYEs so that higher protein levels could cause cellular toxicity from the low-substrate specificities (Lee et al., 2013). Noteworthy, in the complementation analysis, we tried to use a complemented strain in which *ofrA* transcription is initiated *via* xylose-dependent promoter. No growth could be noticed using 0.5% xylose for overexpression possibly because of the aforementioned cellular-mediated toxicities from inducing a high gene dosage of *ofrA* or overconsuming the reducing equivalents NAD(P)H.

In agreement to our β-galactosidase reporter system, and RT-qPCR validation, *ofrA* (SACOL0959 in *S. aureus* COL and SA0817 in *S. aureus* N315) upregulation could be observed in previous transcriptome studies in the presence of MHQ (Fritsch et al., 2019), NaOCl (Loi et al., 2018), and H_2_O_2_ (Chang et al., 2006). Moreover, reactive sulfur species (RSS) result in *ofrA* induction in *S. aureus* (Peng et al., 2017; Loi et al., 2019). Since *ofrA* induction conditions include RES, ROS, RCS, and RSS, we believe that *ofrA* transcriptional regulation responds to a wide variety of conditions that disrupts the redox homeostasis.

The OYE NemA of *E. coli* was reported to be important in hypochlorite stress (Gray et al., 2013; Lee et al., 2013; Ozyamak et al., 2013). Remarkedly, in *S. aureus*, we observed that OfrA is important in protecting against intoxication by ROS and toxic aldehydes in addition to hypochlorite stress. Therefore, we conclude that the compromised survival phenotype of *ofrA* mutant after oxidative, electrophilic, and hypochlorite stress could be due to a defect in a common redox-balancing mechanism important in the three conditions. Most likely, this involves thiol-disulfide homeostasis of so far unknown proteins as shown by our quenching experiments using NAC and thiourea. In relation, methylglyoxal is detoxified *via* both thiol-dependent and -independent pathways. In agreement to our latter conclusion, the thiol-dependent mechanism is the essential pathway for *S. aureus* survival against methylglyoxal (Imber et al., 2018).

In the classical mevalonate pathway, HMG-CoA reductase is the rate-limiting step for the mevalonate production and essential for *S. aureus* growth in the absence of mevalonate supplementation (Wilding et al., 2000; Matsumoto et al., 2016). HMG-CoA reductase uses NAD(P)H as a reducing equivalent for the mevalonate production. Therefore, the availability of NAD(P)H could be the critical factor to explain the decreased mevalonate production and hence the staphyloxanthin production. *S. aureus* JE2Δ*ofrA* has a decreased levels of staphyloxanthin compared to its parental strain (Figures 5A–D). Since OYEs use the reducing equivalents NAD(P)H to regenerate their prosthetic FMN group as an integral part of their activity (Toogood et al., 2010), we believe that *ofrA* mutation affects the NAD(P)H/NAD(P) ratio in *S. aureus* and staphyloxanthin production.

*S. aureus*Δ*ofrA* shows a quick survival defect in whole human blood. Neutrophils in the human blood represent 60% of the leukocyte population and kill the invading bacteria *via* ROS. We assume that *ofrA* mutation-dependent killing mechanism in whole blood is due to the ROS generated by neutrophils. One possibility could be that higher levels of intracellular iron could indirectly enhance the production of HO⋅ *via* Fenton reaction and result in higher toxicities from the same dose of H_2_O_2_ (Wang and Zhao, 2009). However, we could exclude these mechanisms as growth inhibition experiments using streptonigrin, which requires intracellular iron for its antimicrobial activities (White and Yeowell, 1982; Duggan et al., 2020), have shown a similar growth of WT and mutant strains indicating that both strains contain comparable amounts of intracellular iron (Supplementary Figure 7). Since also the survival rate of the mutant in macrophages was reduced, we conclude that OfrA is an important factor to resist killing of *S. aureus* by redox-based molecules produced within phagocytes. Interestingly, a knockout of NTR2 gene, which encodes for an OYE orthologous to OfrA, in the parasite *Leishmania* results in reduced replication within macrophages (Wyllie et al., 2016). Therefore, OYEs could function as anti-stress mechanism included in different eukaryotic and bacterial backgrounds with chromosomal evolution for better fitting the special niche of the organism.

In our attempt to understand the role of OfrA in *S. aureus*, we investigated a transcriptomic approach. *ofrA* mutation leads to slight transcriptomic changes at standard growth conditions in RPMI. Although no specific pattern of deregulated genes could be found, a number of genes involved in redox and stress-related mechanisms were affected in the mutant which reflects the proposed broad substrate specificity of OYEs. The transcriptome data are in line with our hypothesis that OfrA is a member of redox buffering systems that regularly functions under stress and is linked to energy metabolism. Since OfrA has a proposed function in thiol-dependent redox homeostasis, we believe that a targeted thiol redox proteomic approach will be a promising approach in studying the effect of *ofrA* mutation.

**FIGURE 7:**
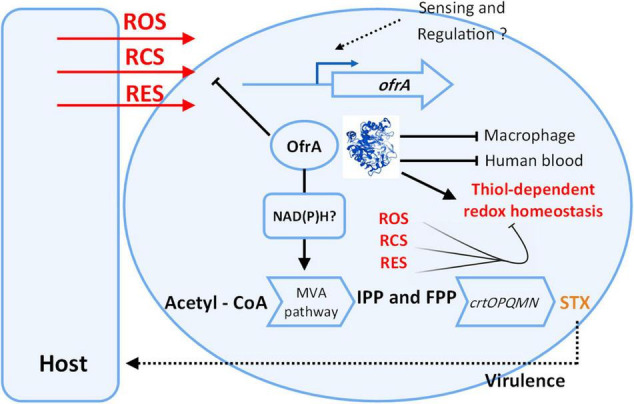
Cartoon representation shows our current understanding of *ofrA* function in *S. aureus*. OfrA protein 3D structure was predicted *via* AlphaFold. For 3D visualization, refer to alphafold.ebi.ac.uk/entry/Q2FZU7. *ofrA* is induced in ROS, RES, and RCS conditions which are available at the host-*S. aureus* interface. We showed that *ofrA* is an important factor in *S. aureus* resistance to the aforementioned stress conditions. *ofrA* contributes to *S. aureus* virulence *via* human blood and macrophage survival. *ofrA* mutation is involved in decreased STX production *via* MVA pathway. Both STX and *ofrA* protects *S. aureus* against oxidative stress *via* different mechanisms. *ofrA* supports the thiol-dependent redox homeostasis. FPP, farnesyl pyrophosphate; IPP, isopentenyl pyrophosphate; MVA, mevalonate; STX, staphyloxanthin.

We present our current understanding of OfrA functions in *S. aureus* based on our results in Figure 7. Our findings suggest that OfrA participates in oxidative, hypochlorite, and electrophilic stress mediation. This has relevance at the bacteria–host interface as OfrA supports intra-macrophage replication and survival. Moreover, OfrA protects *S. aureus* against killing in whole human blood. In addition, STX production is inhibited in the *ofrA* mutant *via* the upper mevalonate pathway, which is, however, not the main mechanism of OfrA-mediated protection against ROS. Overall, we provide evidence that OfrA protects *S. aureus* against numerous stress types through thiol-dependent redox homeostasis.

## Data Availability Statement

The datasets presented in this study can be found in online repositories. The RNA-seq datasets for this study can be found in the NCBI’s Gene Expression Omnibus (https://www.ncbi.nlm.nih.gov/geo/query/acc.cgi?acc=GSE196683). The raw sequenced reads of the whole-genome sequencing are deposited in the SRA database (BioProject ID: PRJNA812552).

## Author Contributions

EI performed the experimental work, designed the experiments, analyzed the data, and wrote the manuscript. KO supervised the project, obtained the funding, discussed the data, and revised the manuscript. Both authors contributed to the article and approved the submitted version.

## Conflict of Interest

The authors declare that the research was conducted in the absence of any commercial or financial relationships that could be construed as a potential conflict of interest.

## Publisher’s Note

All claims expressed in this article are solely those of the authors and do not necessarily represent those of their affiliated organizations, or those of the publisher, the editors and the reviewers. Any product that may be evaluated in this article, or claim that may be made by its manufacturer, is not guaranteed or endorsed by the publisher.

## Footnotes

1
https://www.ncbi.nlm.nih.gov/genome/browse/

2
https://www.ebi.ac.uk/Tools/msa/clustalo/
