# Supplementary material for: The Old Yellow Enzyme OfrA Fosters Staphylococcus aureus Survival via Affecting Thiol-Dependent Redox Homeostasis
Source: Front Microbiol. 2022 May 17;13:888140. doi: 10.3389/fmicb.2022.888140 (PMC9152700; doi:10.3389/fmicb.2022.888140)
Supplement: Supplementary file 1 [file Data_Sheet_1.pdf]

Supplementary Material

Supplementary Figure Legends

Figure S1. Conservation of OfrA and OfrA-orthologues in Firmicutes.

WP_000838037.1 was a query of blastp and searched against proteins encoded in different *S. aureus* strains (A) and *Staphylococcus* species (B).

Figure S2. OfrA distribution in selected Firmicutes.

Blast analysis in *Bacillus* (A), *Clostridium* (B), *Staphylococcus* (C), and *Lactobacillus* (D) species shows OfrA-encoding genes in some genomes. For more information, please, refer to Supplementary Table 4. Filled and unfilled circles indicate gene presence and absence, respectively.

Figure S3. Reporter strain (EI011) construction.

A) Reporter strain construction strategy. P_{ofrA} was cloned upstream to a promoter-less *lacZ*. The β-galactosidase activity is monitored by converting the non-fluorescent substrate (MUG) into the trackable fluorescent MU.

B) Genetic map shows the *lacZ*-reporter system as a chromosomal integrant. Genes from left to right are the following; SAUSA300_0858, *lacZ*, pKO10-backbone, SAUSA300_0858, *ofrA*, *rocD*.

Figure S4. Bacterial growth with the exposure to the testing concentrations of stressors.

Growth of the mid-logarithmic phase *S. aureus* JE2 were monitored by OD_{500} in RPMI medium. We added the stress compounds at the tested concentrations when we considered the supplementation time as $t = 0$. Data represent three biological replicates. Error bars represent the standard deviation.

Figure S5. Growth kinetics of *S. aureus* JE2, Δ*ofrA*, and p*ofrA* in RPMI medium.

Logarithmic phase cells of the three strains were diluted in pre-warmed fresh RPMI medium to the early logarithmic phase and allowed to grow until the stationary phase was reached. We considered the dilution time as $t = 0$. Data represent three biological replicates. Error bars represent the standard deviation.

Figure S6. RT-qPCR validating the RNA-seq experiment results.

RNA samples subjected to RNA-seq were used for cDNA synthesis. Data represent three biological replicates. Statistical analysis was done using unpaired two-tailed Student *t*-test; $*p < 0.05$, $**p < 0.01$.
Error bars represent the standard error of the mean.

Figure S7. JE2 and EI046 have comparable intracellular iron levels.

Growth inhibition assay shows insignificant difference between JE2 and Δ*ofrA* (EI046) in streptonigrin (related to intracellular iron levels) after 8 hr (A), 12 hr (B), 16 hr (C), and 20 hr (D). Data represent three biological replicates. Error bars represent the standard deviation.

Supplementary Tables Legends

Table S1. Bacterial strains used in this study.

Table S2. Oligos used in this study.

Table S3. The distribution of OfrA orthologues in *S. aureus* strains.

Table S4. The distribution of OfrA orthologues in Firmicutes.

Table S5. Genes deregulated in EI046/JE2 strain with P-value < 0.05.

Table S6. GSEA analysis results.

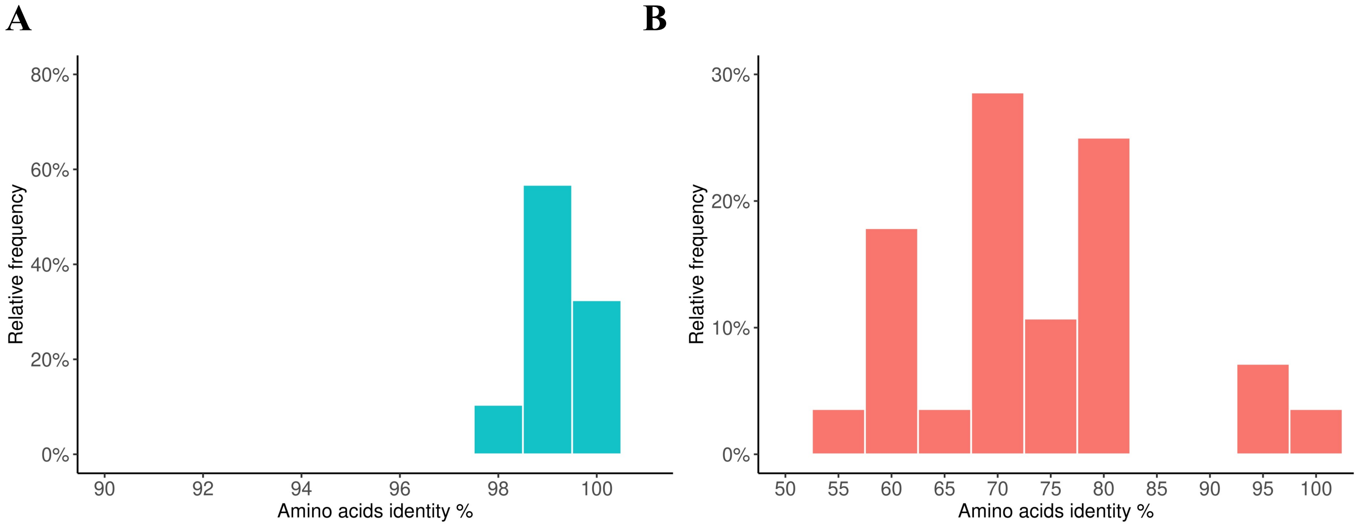

Figure S1. Conservation of OfrA and OfrA-orthologues in Firmicutes.

WP_000838037.1 was a query of blastp and searched against proteins encoded in different *S. aureus* strains (A) and *Staphylococcus* species (B).

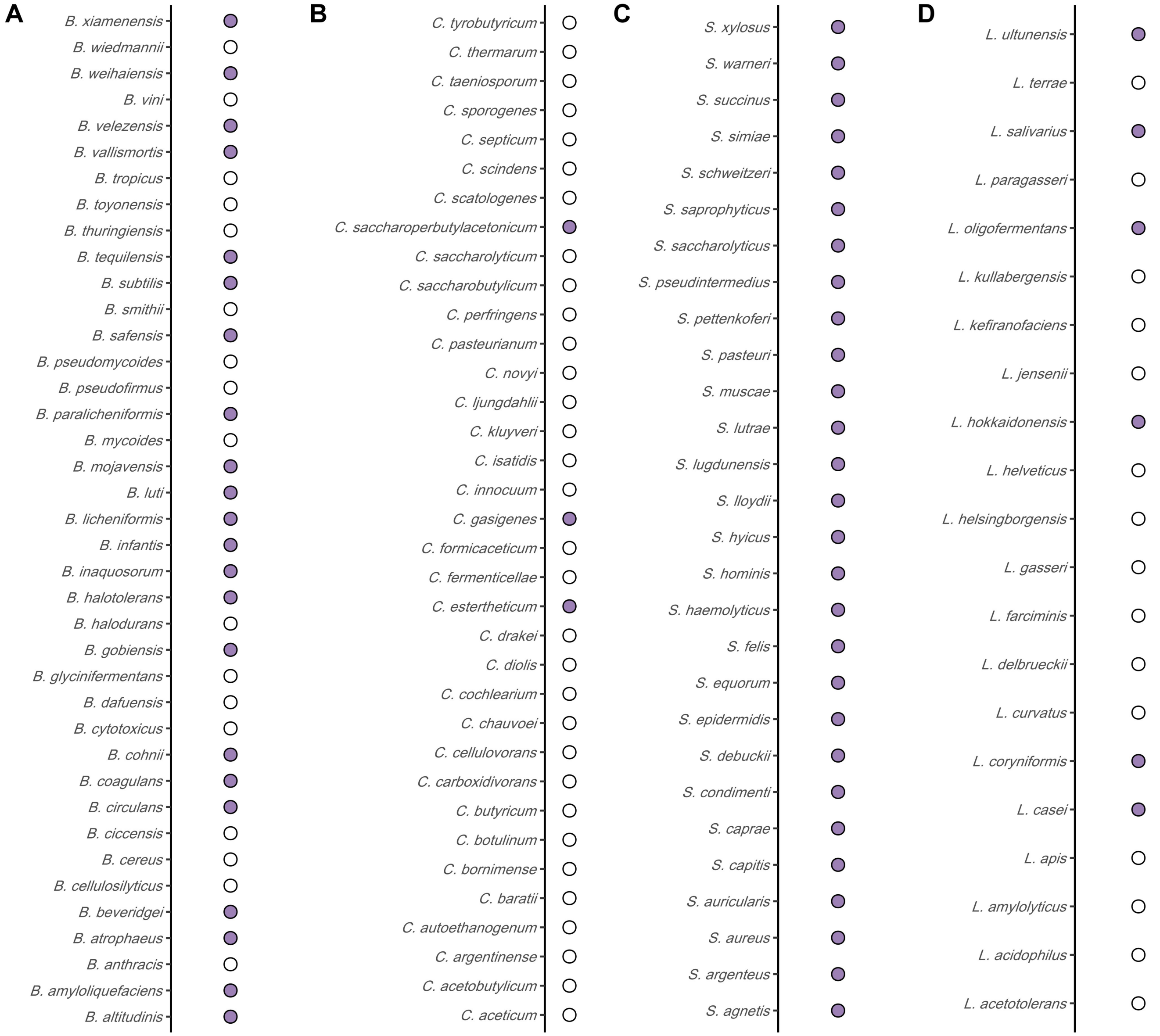

Figure S2. OfrA distribution in selected Firmicutes.

Blast analysis in *Bacillus* (A), *Clostridium* (B), *Staphylococcus* (C), and *Lactobacillus* (D) species shows OfrA-encoding genes in some genomes. For more information, please, refer to Supplementary Table 4. Filled and unfilled circles indicate gene presence and absence, respectively.

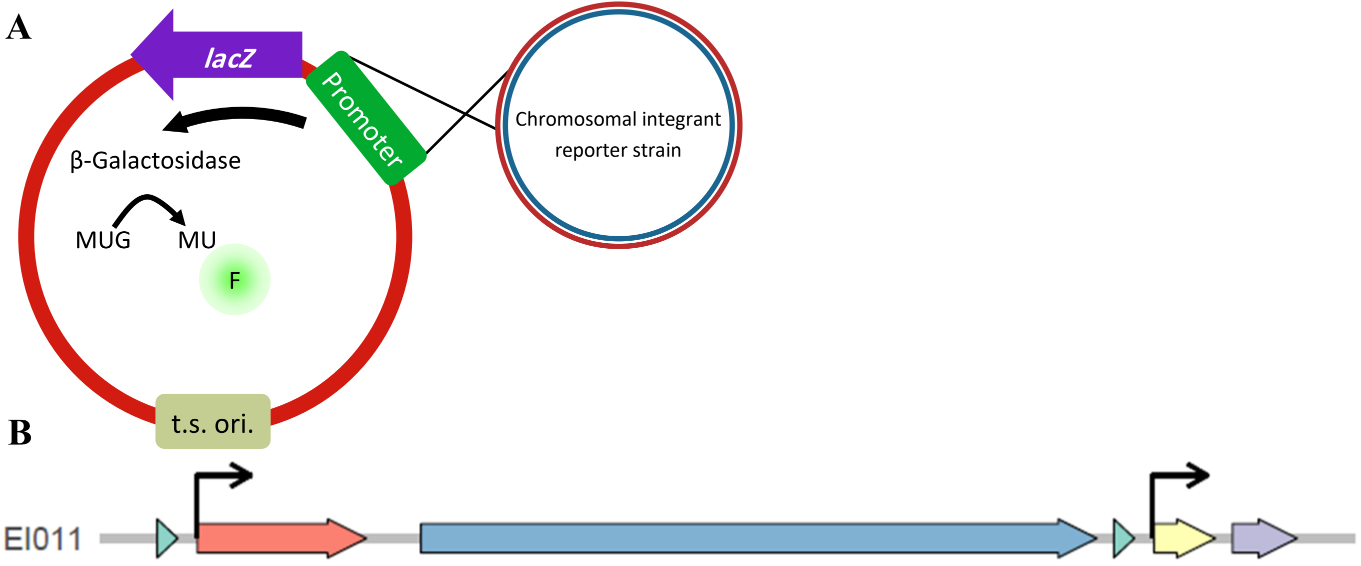

Figure S3. Reporter strain (EI011) construction.

A) Reporter strain construction strategy. P_{ofrA} was cloned upstream to a promoter-less *lacZ*. The β-galactosidase activity is monitored by converting the non-fluorescent substrate (MUG) into the trackable fluorescent MU.

B) Genetic map shows the *lacZ*-reporter system as a chromosomal integrant. Genes from left to right are the following; SAUSA300_0858, *lacZ*, pKO10-backbone, SAUSA300_0858, *ofrA*, *rocD*.

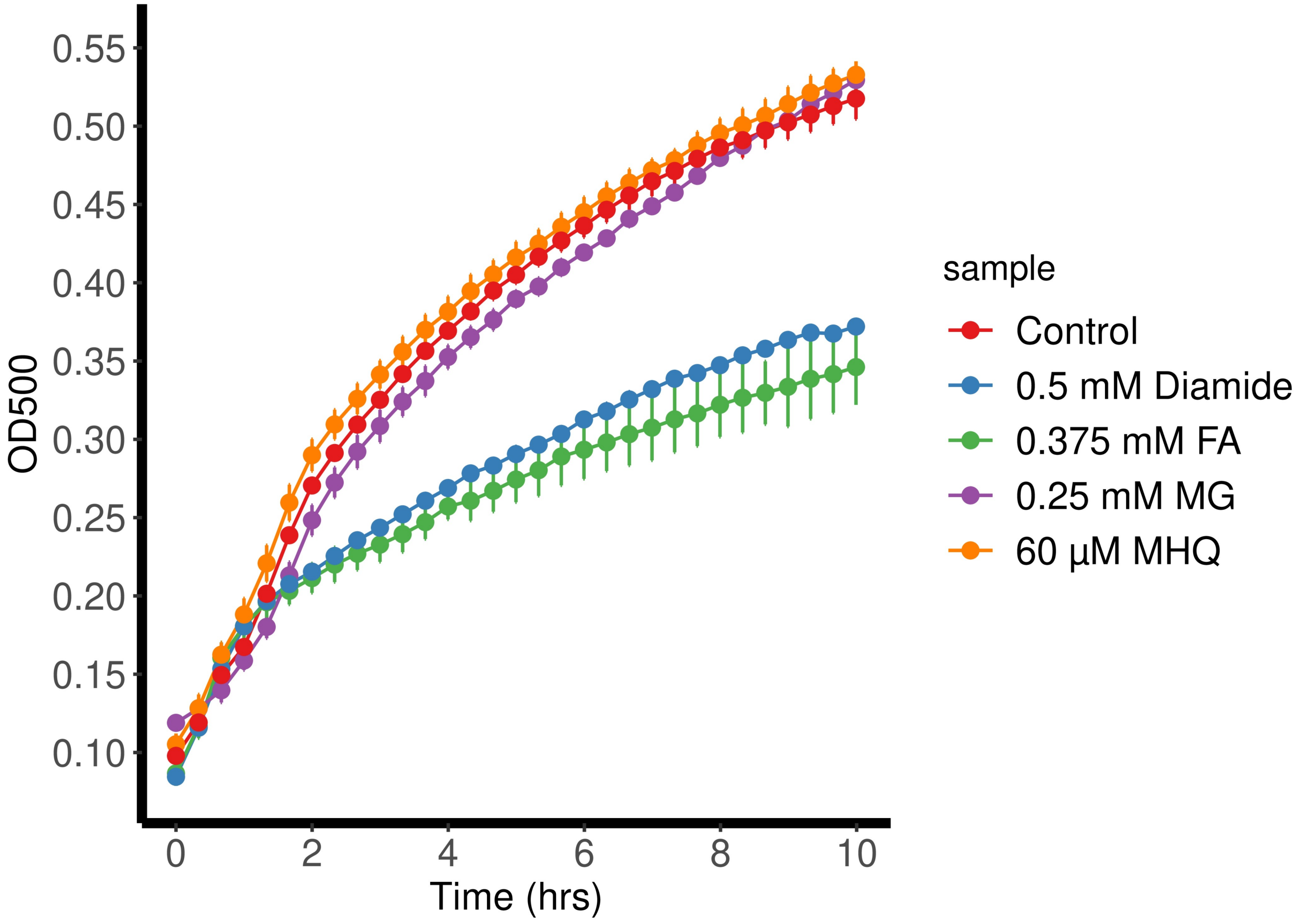

Figure S4. Bacterial growth with the exposure to the testing concentrations of stressors.

Growth of the mid-logarithmic phase *S. aureus* JE2 were monitored by OD_{500} in RPMI medium. We added the stress compounds at the tested concentrations when we considered the supplementation time as $t = 0$. Data represent three biological replicates. Error bars represent the standard deviation.

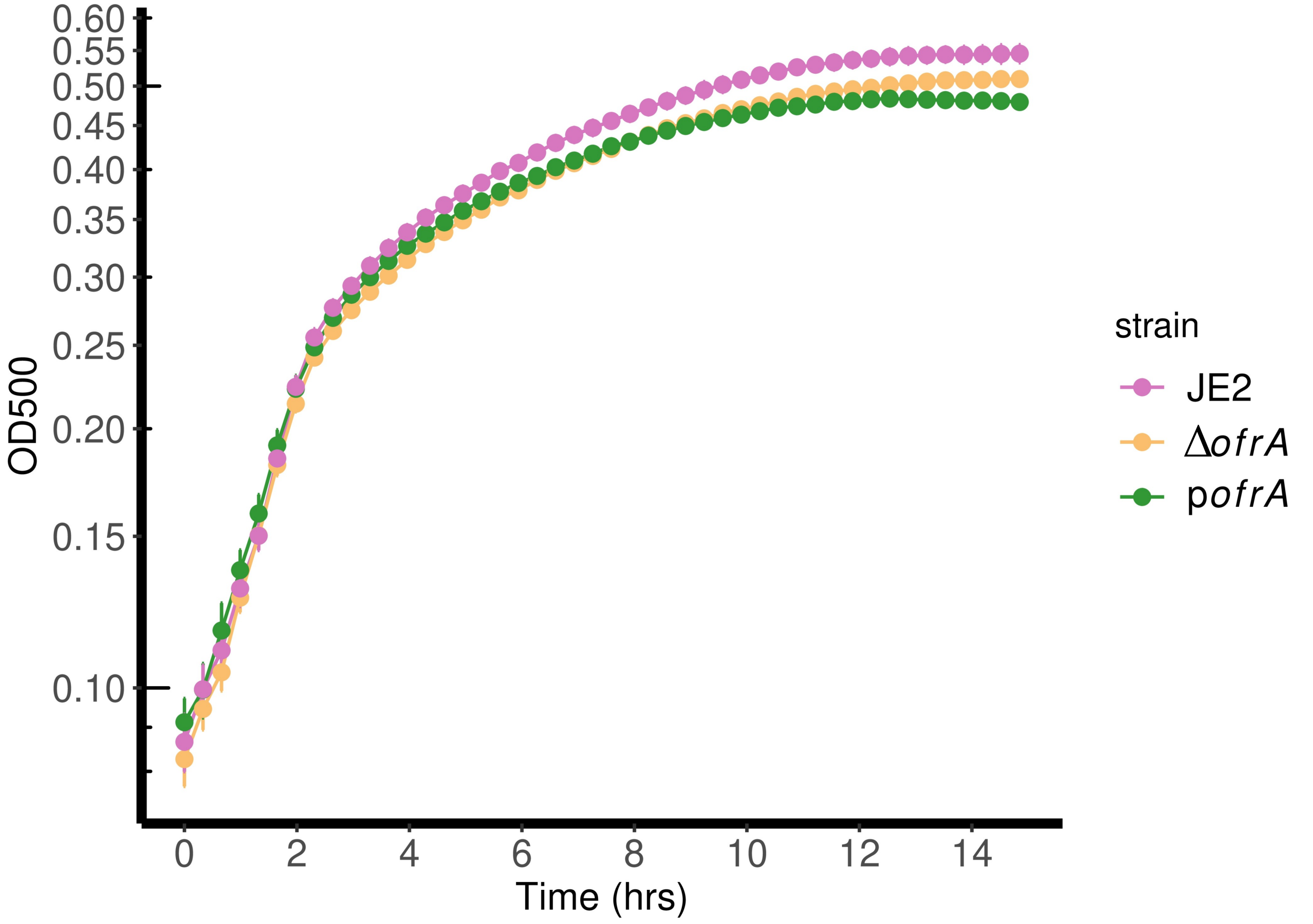

Figure S5. Growth kinetics of *S. aureus* JE2, Δ*ofrA*, and p*ofrA* in RPMI medium.

Logarithmic phase cells of the three strains were diluted in pre-warmed fresh RPMI medium to the early logarithmic phase and allowed to grow until the stationary phase was reached. We considered the dilution time as $t = 0$. Data represent three biological replicates. Error bars represent the standard deviation.

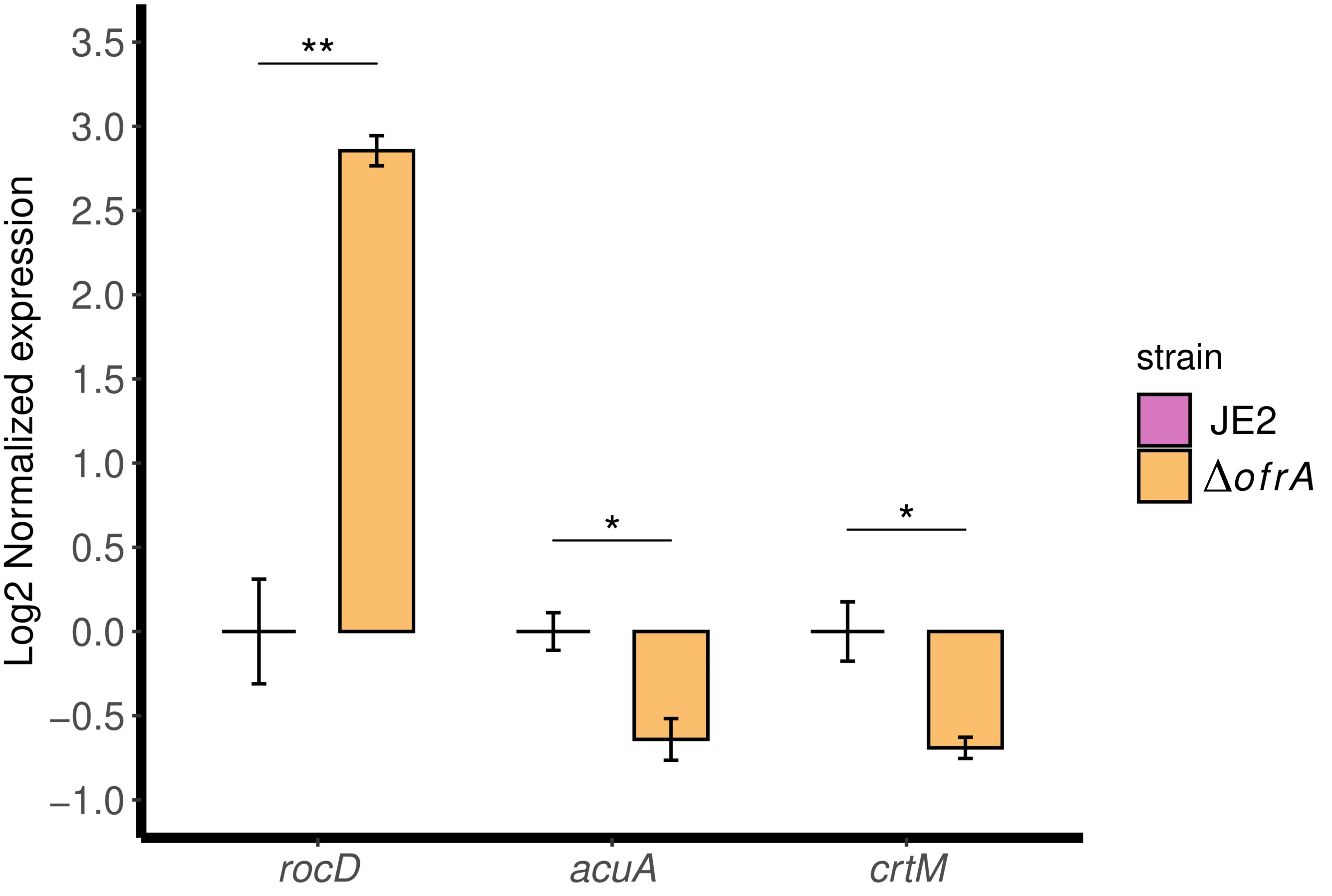

Figure S6. RT-qPCR validating the RNA-seq experiment results.

RNA samples subjected to RNA-seq were used for cDNA synthesis. Data represent three biological replicates. Statistical analysis was done using unpaired two-tailed Student *t*-test; *$p < 0.05$, **$p < 0.01$. Error bars represent the standard error of the mean .

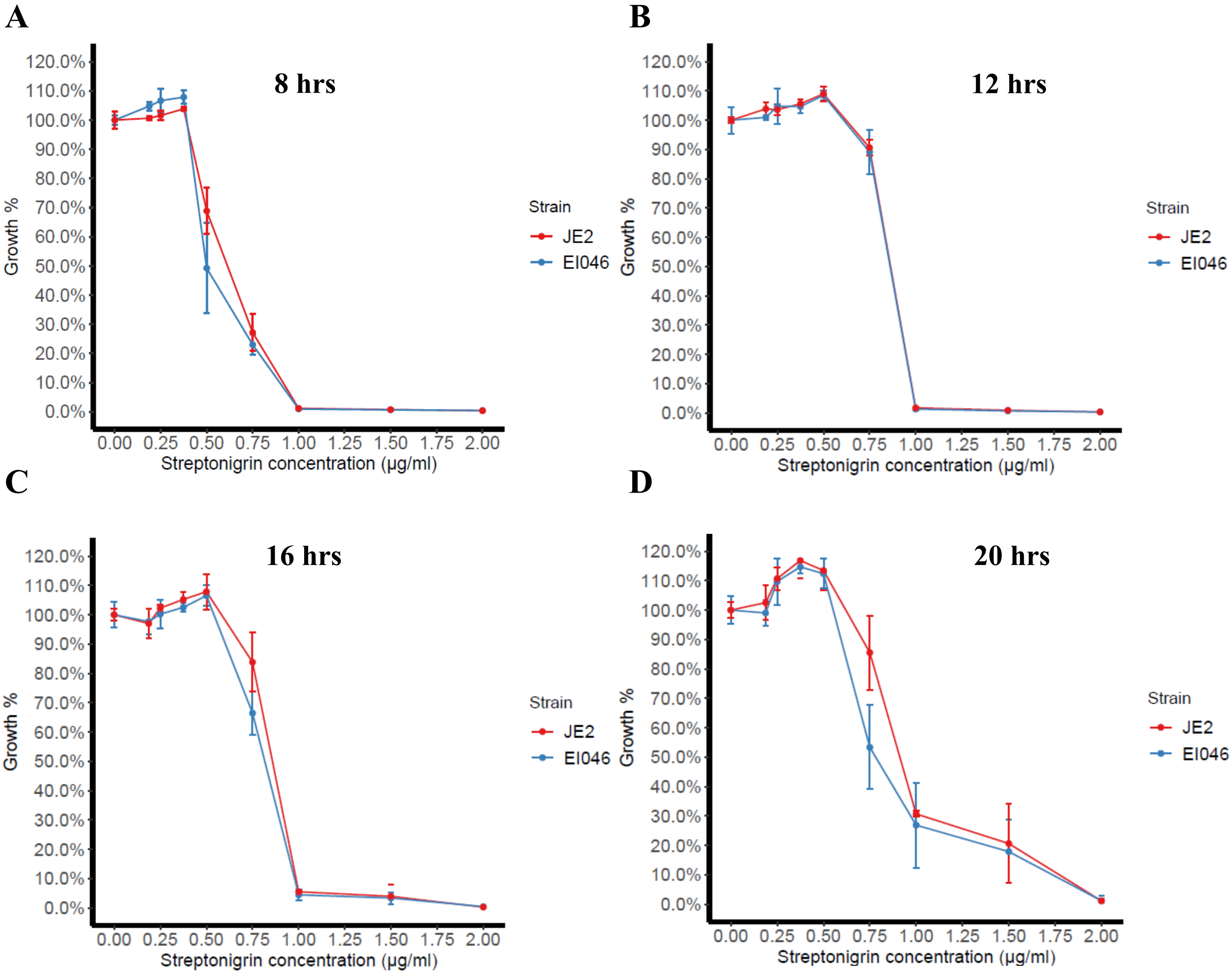

Figure S7. JE2 and EI046 have comparable intracellular iron levels.

Growth inhibition assay shows insignificant difference between JE2 and Δ*ofrA* (EI046) in streptonigrin (related to intracellular iron levels) after 8 hr (A), 12 hr (B), 16 hr (C), and 20 hr (D). Data represent three biological replicates. Error bars represent the standard deviation.

Table S1. Bacterial strains used in this study.

Strain	Description	Reference
Escherichia coli		
DH5α	F-endA1 hsdR17 supE44 thi-1 recA1 gyrA96 relA1 Δ(argFlacZYA) U169 (Φ60ΔlacZ M15λ-)	MBI Fermentas., Waltham (MA), USA
DH5α pKO10::P_{ofrA}-*lacZ*	Construction of reporter plasmid	This study
DH5α pBASE6Δ*ofrA*	Construction of deletion plasmid	This study
DH5α pRB473	empty plasmid	PD.Dr. Wilma Ziebuhr
DH5α pRB473-*ofrA*	Construction of complementation plasmid	This study
IM08B	mcrA Δ(mrr-hsdRMS-mcrBC) φ80lacZΔM15 ΔlacX74 recA1 araD139 Δ(ara-leu)7697 galU galK rpsL endA1 nupG Δdcm ΩPhelp-hsdMS (CC8-2) ΩPN25-hsdS (CC8-1)	
IM08B pBASE6Δ*ofrA*	deletion plasmid	This study
IM08B pRB473-*ofrA*	complementation plasmid	This study
Staphylococcus aureus		
JE2	CA-MRSA, USA300 Isolate, no plasmids, ErmS	
JE2 pKO10::P_{ofrA}-*lacZ*	Temperature sensitive reporter plasmid in *S. aureus* JE2 strain	This study
EI011 (JE2::P_{ofrA}-*lacZ*)	chromosomal integrant reporter strain	This study
EI046 (*S. aureus* JE2Δ*ofrA*)	marker-less deletion mutant	This study
EI047 (*S. aureus* JE2Δ*ofrA::*pRB473-*ofrA*)	complemented strain	This study
NewmannΔ*crtM::cat*	deletion mutant in *crtM* replaced by *cat* (chloramphenicol resistance)	Prof. Friedrich Götz
EI051 (*S. aureus* JE2Δ*crtM::cat*)	deletion mutant of JE2 strain in *crtM* replaced by *cat*(chloramphenicol resistance)	This study
EI052 (*S. aureus* JE2Δ*ofrA*Δ*crtM::cat*)	deletion mutant of EI046 strain in *crtM* replaced by *cat*(chloramphenicol resistance)	This study

Table S2. Oligos used in this study.

Name	Sequence
Sequencing primers	
Δ*ofrA*_seq	ATTGGAGAATGAAAAAATTACATG
pKO10_seq	CGATTAAGTTGGGTAACGC
pBASE.1	AAATTACGCCCCGCCCTG
pBASE.2	CATCAACAATCCGTTCTGC
*ofrA*_report_up	GCAATCTCAATATATTTATCAAGAAAGC
cDNA synthesis and qPCR primers	
tag.*ofrA*.cDNA	ACTTAGCGCGGGAACACGCATGATGTATTTGTACGAGTGC
tag.qPCR	ACTTAGCGCGGGAACACG
ofrA.qPCR	GCCATCAATCGCGCATGACA
rpoB.qPCR.1	CTAAGCACAGAGGTCGT
rpoB.qPCR.2	ACGGCATCCTCATAGT
rho.qPCR.1	GAAGCTGCTGAAGTCG
rho.qPCR.2	CGTCCATACGTGAACCC
acuA.qPCR.1	TGGGGGCAATTGAAGTCAGC
acuA.qPCR.2	CACCAGTTGCCATTAACCGCT
crtM.qPCR.1	GCAACATGCTGAAGCGCCA
crtM.qPCR.2	TCAAAGAAAAGCGGTTTGGGCA
rocD.qPCR.1	AGCAGGATTAGGTCGTTCGGG
rocD.qPCR.2	TGAACCATGTGAGCCAGGTGT
Cloning primers	
P*ofrA*_pKO10.1	GGCCCTTTCGTCTTCAATTAAAGATAATAGTTGAGGTTGC
P*ofrA*_pKO10.2	CAACAAGCTGGGGATCTTTACTTAATTCCTCCTTAAAATTATTGAG
0857_0860_pES2.BamHI.1	CCGGGATCCGGACATCGTCTCCATTTTCTTCAACAATCGTGACACC
0857_0860_pES2.XhoI.2	CCGCTCGAGGTGCCATCATCCATTCGTACAGGGATACGCAC
*ofrA*_up_pBASE6.SacI.1	CCGGAGCTCGTGGCTGGCCTCAACC
*ofrA*_down_pBASE6.NheI.2	CCGGCTAGCCTAATACAACAGAAATTGGGAAGACTC
*ofrA*_pRB473.EcoRI.1	CCGGAATTCAACAGAAAAATATGGGTTTCAAAC
*ofrA*_pRB473.BamHI.2	CCGGGATCCAGAATTGATAATTTTACACTTAATCAAAAG
PCR-mediated plasmid deletions primers	
ofrA.del.1	GATAAATCAATTCTTTTGCTTGTAATCATTTAAGTAATTATGTCATTATAAATGTAAGGG
ofrA.del.2	TAAATGATTACAAGCAAAAGAATTGATTTATCTGTTCCTTTAGTCGTTTCGAATTGCTCG

Table S3. The distribution of OfrA orthologues in *S. aureus* strains.

Organism Name	Chromosome	presence	aa_identity	aa_similarity	alignment_length	protein_id	protein_length
Staphylococcus aureus subsp. aureus NCTC 8325	NC_007795.1	TRUE	100	100	375	YP_499446.1	375
Staphylococcus aureus	NZ_CP011526.1	TRUE	100	100	375	WP_000838037.1	375
Staphylococcus aureus	NZ_CP035101.1	TRUE	100	100	375	WP_000838037.1	375
Staphylococcus aureus	NZ_CP040998.1	TRUE	100	100	375	WP_000838037.1	375
Staphylococcus aureus	NZ_CP018629.1	TRUE	100	100	375	WP_000838037.1	375
Staphylococcus aureus	NZ_CP013957.1	TRUE	100	100	375	WP_000838037.1	375
Staphylococcus aureus	NZ_AP019306.1	TRUE	99.467	99.73333333	375	WP_000838029.1	375
Staphylococcus aureus	NZ_LR027878.1	TRUE	100	100	375	WP_000838037.1	375
Staphylococcus aureus	NZ_CP030138.1	TRUE	100	100	375	WP_000838037.1	375
Staphylococcus aureus	NZ_LR130515.1	TRUE	100	100	375	WP_000838037.1	375
Staphylococcus aureus	NZ_CP012011.1	TRUE	100	100	375	WP_000838037.1	375
Staphylococcus aureus	NZ_CP009681.1	TRUE	100	100	375	WP_000838037.1	375
Staphylococcus aureus	NZ_CP027788.1	TRUE	100	100	375	WP_000838037.1	375
Staphylococcus aureus subsp. aureus TW20	NC_017331.1	TRUE	100	100	375	WP_000838037.1	375
Staphylococcus aureus	NZ_CP015447.2	TRUE	100	100	375	WP_000838037.1	375
Staphylococcus aureus	NZ_LR027874.1	TRUE	100	100	375	WP_000838037.1	375
Staphylococcus aureus	NZ_CP012013.1	TRUE	100	100	375	WP_000838037.1	375
Staphylococcus aureus	NZ_CP012018.1	TRUE	100	100	375	WP_000838037.1	375
Staphylococcus aureus subsp. aureus Z172	NC_022604.1	TRUE	100	100	375	WP_000838037.1	375
Staphylococcus aureus	NZ_CP012015.1	TRUE	100	100	375	WP_000838037.1	375
Staphylococcus aureus	NC_021670.1	TRUE	100	100	375	WP_000838037.1	375
Staphylococcus aureus	NZ_LR027869.1	TRUE	100	100	375	WP_000838037.1	375
Staphylococcus aureus	NZ_CP029198.1	TRUE	99.467	99.73333333	375	WP_000838029.1	375
Staphylococcus aureus	NZ_CP012012.1	TRUE	100	100	375	WP_000838037.1	375
Staphylococcus aureus	NZ_LR822061.1	TRUE	99.467	99.73333333	375	WP_000838029.1	375
Staphylococcus aureus	NZ_LR822060.1	TRUE	99.467	99.73333333	375	WP_000838029.1	375
Staphylococcus aureus	NZ_CP039157.1	TRUE	99.467	100	375	WP_000838016.1	375
Staphylococcus aureus	NZ_CP058312.1	TRUE	100	100	375	WP_000838037.1	375
Staphylococcus aureus	NZ_CP041010.1	TRUE	100	100	375	WP_000838037.1	375
Staphylococcus aureus	NZ_CP029653.1	TRUE	97.867	98.93333333	375	WP_000838046.1	375
Staphylococcus aureus	NZ_AP019305.1	TRUE	99.467	99.73333333	375	WP_000838029.1	375

Staphylococcus aureus	NZ_AP019543.1	TRUE	99.467	99.73333333	375	WP_000838029.1	375
Staphylococcus aureus	NZ_AP019545.1	TRUE	99.467	99.73333333	375	WP_000838029.1	375
Staphylococcus aureus	NZ_LT992462.1	TRUE	98.933	99.73333333	375	WP_000838045.1	375
Staphylococcus aureus	NZ_CP029685.1	TRUE	100	100	375	WP_000838037.1	375
Staphylococcus aureus	NZ_CP026072.1	TRUE	99.467	100	375	WP_000838016.1	375
Staphylococcus aureus	NZ_CP007499.1	TRUE	99.467	100	375	WP_000838016.1	375
Staphylococcus aureus	NZ_LR134351.1	TRUE	97.333	98.66666667	375	WP_129545252.1	375
Staphylococcus aureus	NZ_CP013953.1	TRUE	99.467	99.73333333	375	WP_000838029.1	375
Staphylococcus aureus	NZ_CP020960.1	TRUE	100	100	375	WP_000838037.1	375
Staphylococcus aureus	NZ_LS483309.1	TRUE	100	100	375	WP_000838037.1	375
Staphylococcus aureus	NZ_CP047781.1	TRUE	97.6	98.93333333	375	WP_000838047.1	375
Staphylococcus aureus	NZ_LS483319.1	TRUE	100	100	375	WP_000838037.1	375
Staphylococcus aureus	NZ_CP047782.1	TRUE	97.6	98.93333333	375	WP_000838047.1	375
Staphylococcus aureus	NZ_AP019542.1	TRUE	99.467	99.73333333	375	WP_000838029.1	375
Staphylococcus aureus	NZ_CP043302.1	TRUE	99.467	100	375	WP_000838016.1	375
Staphylococcus aureus	NZ_AP020320.1	TRUE	99.467	99.73333333	375	WP_000838029.1	375
Staphylococcus aureus	NZ_CP040625.1	TRUE	100	100	375	WP_000838037.1	375
Staphylococcus aureus	NZ_CP040622.1	TRUE	100	100	375	WP_000838037.1	375
Staphylococcus aureus	NZ_LR027877.1	TRUE	100	100	375	WP_000838037.1	375
Staphylococcus aureus	NZ_LS483301.1	TRUE	100	100	375	WP_000838037.1	375
Staphylococcus aureus	NZ_CP022908.1	TRUE	100	100	375	WP_000838037.1	375
Staphylococcus aureus	NZ_CP062467.1	TRUE	99.2	99.2	375	WP_000838041.1	375
Staphylococcus aureus	NZ_CP047801.1	TRUE	97.6	98.93333333	375	WP_000838047.1	375
Staphylococcus aureus	NZ_LR027876.1	TRUE	100	100	375	WP_000838037.1	375
Staphylococcus aureus	NZ_LR027873.1	TRUE	100	100	375	WP_000838037.1	375
Staphylococcus aureus subsp. aureus str. JKD6008	NC_017341.1	TRUE	100	100	375	WP_000838037.1	375
Staphylococcus aureus	NZ_LT992471.1	TRUE	98.933	99.73333333	375	WP_000838045.1	375
Staphylococcus aureus	NZ_CP029678.1	TRUE	100	100	375	WP_000838037.1	375
Staphylococcus aureus USA300-ISMMS1	NZ_CP007176.1	TRUE	100	100	375	WP_000838037.1	375
Staphylococcus aureus	NZ_CP014415.1	TRUE	100	100	375	WP_000838037.1	375
Staphylococcus aureus	NZ_CP014444.1	TRUE	100	100	375	WP_000838037.1	375
Staphylococcus aureus	NZ_CP014429.1	TRUE	100	100	375	WP_000838037.1	375
Staphylococcus aureus	NZ_CP014412.1	TRUE	100	100	375	WP_000838037.1	375
Staphylococcus aureus	NZ_CP014420.1	TRUE	100	100	375	WP_000838037.1	375

Staphylococcus aureus	NZ_CP014426.1	TRUE	100	100	375	WP_000838037.1	375
Staphylococcus aureus	NZ_CP014423.1	TRUE	100	100	375	WP_000838037.1	375
Staphylococcus aureus	NZ_CP014409.1	TRUE	100	100	375	WP_000838037.1	375
Staphylococcus aureus	NZ_CP014435.1	TRUE	100	100	375	WP_000838037.1	375
Staphylococcus aureus	NZ_CP014381.1	TRUE	100	100	375	WP_000838037.1	375
Staphylococcus aureus	NZ_CP014368.1	TRUE	100	100	375	WP_000838037.1	375
Staphylococcus aureus	NZ_CP014384.1	TRUE	100	100	375	WP_000838037.1	375
Staphylococcus aureus	NZ_CP058615.1	TRUE	100	100	375	WP_000838037.1	375
Staphylococcus aureus	NZ_CP014392.1	TRUE	100	100	375	WP_000838037.1	375
Staphylococcus aureus	NZ_CP014397.1	TRUE	100	100	375	WP_000838037.1	375
Staphylococcus aureus	NZ_CP014402.1	TRUE	100	100	375	WP_000838037.1	375
Staphylococcus aureus	NZ_CP014371.1	TRUE	100	100	375	WP_000838037.1	375
Staphylococcus aureus	NZ_CP014376.1	TRUE	100	100	375	WP_000838037.1	375
Staphylococcus aureus	NZ_CP014387.1	TRUE	100	100	375	WP_000838037.1	375
Staphylococcus aureus	NZ_CP012119.2	TRUE	100	100	375	WP_000838037.1	375
Staphylococcus aureus	NZ_CP014432.1	TRUE	100	100	375	WP_000838037.1	375
Staphylococcus aureus	NZ_CP014438.1	TRUE	100	100	375	WP_000838037.1	375
Staphylococcus aureus	NZ_CP014441.1	TRUE	100	100	375	WP_000838037.1	375
Staphylococcus aureus	NZ_CP029166.1	TRUE	99.467	100	375	WP_000838016.1	375
Staphylococcus aureus	NZ_CP031537.1	TRUE	100	100	375	WP_000838037.1	375
Staphylococcus aureus	NZ_AP020324.1	TRUE	99.467	99.73333333	375	WP_000838029.1	375
Staphylococcus aureus subsp. aureus T0131	NC_017347.1	TRUE	100	100	375	WP_000838037.1	375
Staphylococcus aureus	NZ_CP028190.1	TRUE	97.867	98.93333333	375	WP_000838046.1	375
Staphylococcus aureus	NZ_CP022904.1	TRUE	99.467	99.73333333	375	WP_000838029.1	375
Staphylococcus aureus	NZ_CP022903.1	TRUE	99.467	99.73333333	375	WP_000838029.1	375
Staphylococcus aureus	NZ_CP022905.1	TRUE	99.467	99.73333333	375	WP_000838029.1	375
Staphylococcus aureus	NZ_CP022893.1	TRUE	99.467	99.73333333	375	WP_000838029.1	375
Staphylococcus aureus	NZ_CP022894.1	TRUE	99.467	99.73333333	375	WP_000838029.1	375
Staphylococcus aureus	NZ_CP029082.1	TRUE	99.467	99.73333333	375	WP_000838029.1	375
Staphylococcus aureus	NZ_CP029657.1	TRUE	99.467	99.73333333	375	WP_000838029.1	375
Staphylococcus aureus	NZ_CP013955.1	TRUE	99.467	99.73333333	375	WP_000838029.1	375
Staphylococcus aureus	NZ_LT992468.1	TRUE	98.933	99.73333333	375	WP_000838045.1	375
Staphylococcus aureus	NZ_CP029658.1	TRUE	99.467	99.73333333	375	WP_000838029.1	375
Staphylococcus aureus	NZ_CP029680.1	TRUE	99.467	99.73333333	375	WP_000838029.1	375
Staphylococcus aureus	NZ_CP039156.1	TRUE	99.467	99.73333333	375	WP_000838029.1	375

Staphylococcus aureus	NZ_CP029087.1	TRUE	99.467	99.73333333	375	WP_000838029.1	375
Staphylococcus aureus subsp. aureus JH9	NC_009487.1	TRUE	99.467	99.73333333	375	WP_000838029.1	375
Staphylococcus aureus subsp. aureus JH1	NC_009632.1	TRUE	99.467	99.73333333	375	WP_000838029.1	375
Staphylococcus aureus	NZ_CP030326.1	TRUE	99.467	99.73333333	375	WP_000838029.1	375
Staphylococcus aureus	NZ_LT992460.1	TRUE	98.933	99.73333333	375	WP_000838045.1	375
Staphylococcus aureus	NZ_AP019751.1	TRUE	99.467	99.73333333	375	WP_000838033.1	375
Staphylococcus aureus	NZ_CP022582.1	TRUE	99.467	99.73333333	375	WP_000838029.1	375
Staphylococcus aureus	NZ_CP014362.1	TRUE	100	100	375	WP_000838037.1	375
Staphylococcus aureus	NZ_CP014365.1	TRUE	100	100	375	WP_000838037.1	375
Staphylococcus aureus	NZ_LT992475.1	TRUE	98.933	99.73333333	375	WP_000838045.1	375
Staphylococcus aureus	NZ_CP009423.1	TRUE	100	100	375	WP_000838037.1	375
Staphylococcus aureus	NZ_CP012692.1	TRUE	99.467	99.73333333	375	WP_000838029.1	375
Staphylococcus aureus	NZ_CP029172.1	TRUE	98.933	99.73333333	375	WP_000838045.1	375
Staphylococcus aureus subsp. aureus MRSA252	NC_002952.2	TRUE	97.6	98.93333333	375	WP_000838047.1	375
Staphylococcus aureus	NZ_LS483484.1	TRUE	97.6	98.93333333	375	WP_000838047.1	375
Staphylococcus aureus	NZ_CP018766.1	TRUE	99.467	99.73333333	375	WP_000838029.1	375
Staphylococcus aureus	NZ_CP047321.1	TRUE	100	100	375	WP_000838037.1	375
Staphylococcus aureus	NZ_CP013231.1	TRUE	100	100	375	WP_000838037.1	375
Staphylococcus aureus	NZ_CP029667.1	TRUE	100	100	375	WP_000838037.1	375
Staphylococcus aureus	NZ_LT992476.1	TRUE	98.933	99.73333333	375	WP_000838045.1	375
Staphylococcus aureus	NZ_AP017377.1	TRUE	100	100	375	WP_000838037.1	375
Staphylococcus aureus	NZ_CP045435.1	TRUE	99.467	99.73333333	375	WP_000838033.1	375
Staphylococcus aureus	NZ_CP047791.1	TRUE	97.6	98.93333333	375	WP_000838047.1	375
Staphylococcus aureus	NZ_CP007670.1	TRUE	100	100	375	WP_000838037.1	375
Staphylococcus aureus	NZ_CP033112.1	TRUE	97.6	98.93333333	375	WP_000838047.1	375
Staphylococcus aureus	NZ_CP033865.1	TRUE	97.6	98.93333333	375	WP_124095487.1	375
Staphylococcus aureus	NZ_CP013621.1	TRUE	98.933	99.73333333	375	WP_000838045.1	375
Staphylococcus aureus	NZ_CP022906.1	TRUE	100	100	375	WP_000838037.1	375
Staphylococcus aureus	NZ_CP022902.1	TRUE	100	100	375	WP_000838037.1	375
Staphylococcus aureus	NZ_LT992464.1	TRUE	98.933	99.73333333	375	WP_000838045.1	375
Staphylococcus aureus	NZ_LT992461.1	TRUE	98.933	99.73333333	375	WP_000838045.1	375
Staphylococcus aureus	NZ_LR130518.1	TRUE	100	100	375	WP_000838037.1	375
Staphylococcus aureus	NZ_CP062469.1	TRUE	99.467	99.73333333	375	WP_000838029.1	375
Staphylococcus aureus	NZ_CP009554.1	TRUE	97.6	98.93333333	375	WP_000838047.1	375

Staphylococcus aureus	NZ_CP019945.1	TRUE	99.467	99.73333333	375	WP_000838039.1	375
Staphylococcus aureus	NZ_LR027870.1	TRUE	100	100	375	WP_000838037.1	375
Staphylococcus aureus	NZ_CP053075.1	TRUE	99.467	99.73333333	375	WP_000838029.1	375
Staphylococcus aureus	NZ_CP047779.1	TRUE	97.6	98.93333333	375	WP_000838047.1	375
Staphylococcus aureus	NZ_CP060491.1	TRUE	100	100	375	WP_000838037.1	375
Staphylococcus aureus	NZ_CP028163.1	TRUE	98.933	99.73333333	375	WP_000838045.1	375
Staphylococcus aureus	NZ_CP026067.1	TRUE	99.467	100	375	WP_000838024.1	375
Staphylococcus aureus	NZ_CP029664.1	TRUE	100	100	375	WP_000838037.1	375
Staphylococcus aureus	NZ_CP053639.1	TRUE	99.467	100	375	WP_000838016.1	375
Staphylococcus aureus subsp. aureus Mu3	NC_009782.1	TRUE	99.467	99.73333333	375	WP_000838029.1	375
Staphylococcus aureus	NZ_LT992458.1	TRUE	98.933	99.73333333	375	WP_000838045.1	375
Staphylococcus aureus	NZ_CP043386.1	TRUE	100	100	375	WP_000838037.1	375
Staphylococcus aureus	NZ_CP043392.1	TRUE	100	100	375	WP_000838037.1	375
Staphylococcus aureus	NZ_CP043389.1	TRUE	100	100	375	WP_000838037.1	375
Staphylococcus aureus subsp. aureus str. Newman	NC_009641.1	TRUE	100	100	375	WP_000838037.1	375
Staphylococcus aureus	NZ_CP023391.1	TRUE	100	100	375	WP_000838037.1	375
Staphylococcus aureus subsp. aureus str. Newman	NZ_CP023390.1	TRUE	100	100	375	WP_000838037.1	375
Staphylococcus aureus	NZ_LT598688.1	TRUE	100	100	375	WP_000838037.1	375
Staphylococcus aureus	NZ_CP019590.1	TRUE	100	100	375	WP_000838037.1	375
Staphylococcus aureus subsp. aureus Mu50	NC_002758.2	TRUE	99.467	99.73333333	375	WP_000838029.1	375
Staphylococcus aureus	NZ_CP026958.1	TRUE	99.467	99.73333333	375	WP_000838029.1	375
Staphylococcus aureus	NZ_CP026069.1	TRUE	99.467	99.73333333	375	WP_000838029.1	375
Staphylococcus aureus	NZ_CP029675.1	TRUE	99.467	99.73333333	375	WP_000838029.1	375
Staphylococcus aureus	NZ_CP016863.1	TRUE	100	100	375	WP_000838037.1	375
Staphylococcus aureus	NZ_CP026074.1	TRUE	99.467	99.73333333	375	WP_000838029.1	375
Staphylococcus aureus	NZ_CP053356.1	TRUE	99.467	99.73333333	375	WP_000838029.1	375
Staphylococcus aureus	NZ_CP059155.1	TRUE	97.6	98.93333333	375	WP_000838047.1	375
Staphylococcus aureus	NZ_CP058613.1	TRUE	100	100	375	WP_000838037.1	375
Staphylococcus aureus	NZ_CP047809.1	TRUE	99.733	99.73333333	375	WP_161575636.1	375
Staphylococcus aureus	NZ_AP020322.1	TRUE	99.467	99.73333333	375	WP_000838029.1	375
Staphylococcus aureus	NZ_CP034441.1	TRUE	99.467	99.73333333	375	WP_000838033.1	375
Staphylococcus aureus	NZ_CP040232.1	TRUE	98.933	99.73333333	375	WP_000838045.1	375
Staphylococcus aureus	NZ_CP014407.1	TRUE	100	100	375	WP_000838037.1	375

Staphylococcus aureus	NZ_CP031667.1	TRUE	100	100	375	WP_000838037.1	375
Staphylococcus aureus	NZ_CP031670.1	TRUE	100	100	375	WP_000838037.1	375
Staphylococcus aureus	NZ_LT992477.1	TRUE	98.933	99.73333333	375	WP_000838045.1	375
Staphylococcus aureus	NZ_CP035369.1	TRUE	100	100	375	WP_000838037.1	375
Staphylococcus aureus LAC	NZ_CP055225.1	TRUE	100	100	375	WP_000838037.1	375
Staphylococcus aureus	NZ_CP020619.1	TRUE	100	100	375	WP_000838037.1	375
Staphylococcus aureus	NZ_LN626917.1	TRUE	97.6	98.93333333	375	WP_045178726.1	375
Staphylococcus aureus	NZ_CP026961.1	TRUE	100	100	375	WP_000838037.1	375
Staphylococcus aureus	NZ_CP042650.1	TRUE	99.467	99.46666667	375	WP_044288779.1	375
Staphylococcus aureus	NZ_CP016858.1	TRUE	100	100	375	WP_000838037.1	375
Staphylococcus aureus	NZ_CP016855.1	TRUE	100	100	375	WP_000838037.1	375
Staphylococcus aureus	NZ_CP007690.1	TRUE	100	100	375	WP_000838037.1	375
Staphylococcus aureus subsp. aureus USA300_TCH1516	NC_010079.1	TRUE	100	100	375	WP_000838037.1	375
Staphylococcus aureus	NZ_CP010299.1	TRUE	100	100	375	WP_000838037.1	375
Staphylococcus aureus	NZ_CP010296.1	TRUE	100	100	375	WP_000838037.1	375
Staphylococcus aureus	NZ_CP010298.1	TRUE	100	100	375	WP_000838037.1	375
Staphylococcus aureus	NZ_CP010300.1	TRUE	100	100	375	WP_000838037.1	375
Staphylococcus aureus subsp. aureus USA300_FPR3757	NC_007793.1	TRUE	100	100	375	WP_000838037.1	375
Staphylococcus aureus	NZ_CP029031.1	TRUE	100	100	375	WP_000838037.1	375
Staphylococcus aureus	NZ_CP029032.1	TRUE	100	100	375	WP_000838037.1	375
Staphylococcus aureus	NZ_CP010295.1	TRUE	100	100	375	WP_000838037.1	375
Staphylococcus aureus	NZ_CP010297.1	TRUE	100	100	375	WP_000838037.1	375
Staphylococcus aureus subsp. aureus ST398	NC_017333.1	TRUE	98.933	99.73333333	375	WP_000838045.1	375
Staphylococcus aureus	NZ_CP029669.1	TRUE	100	100	375	WP_000838037.1	375
Staphylococcus aureus	NZ_CP047802.1	TRUE	99.467	99.73333333	375	WP_000838029.1	375
Staphylococcus aureus	NZ_LT992469.1	TRUE	98.933	99.73333333	375	WP_000838045.1	375
Staphylococcus aureus	NZ_CP047865.1	TRUE	99.467	99.73333333	375	WP_000838029.1	375
Staphylococcus aureus	NZ_CP040619.1	TRUE	99.2	99.73333333	375	WP_031806857.1	375
Staphylococcus aureus	NZ_CP029751.1	TRUE	99.2	100	375	WP_000838025.1	375
Staphylococcus aureus	NZ_CP047856.1	TRUE	99.467	99.73333333	375	WP_000838029.1	375
Staphylococcus aureus	NZ_LS483316.1	TRUE	100	100	375	WP_000838037.1	375
Staphylococcus aureus	NZ_LT992473.1	TRUE	98.933	99.73333333	375	WP_000838045.1	375
Staphylococcus aureus	CP053353.1	TRUE	99.467	100	375	QKL43590.1	375
Staphylococcus aureus	NZ_CP029663.1	TRUE	99.467	99.73333333	375	WP_000838029.1	375

Staphylococcus aureus	NZ_LT992467.1	TRUE	98.933	99.73333333	375	WP_000838045.1	375
Staphylococcus aureus	NZ_CP039167.1	TRUE	99.2	99.2	375	WP_000838041.1	375
Staphylococcus aureus	NZ_CP031890.1	TRUE	100	100	375	WP_000838037.1	375
Staphylococcus aureus	NZ_CP031888.1	TRUE	100	100	375	WP_000838037.1	375
Staphylococcus aureus	NZ_CP033987.1	TRUE	99.467	99.73333333	375	WP_000838029.1	375
Staphylococcus aureus	NZ_CP033982.1	TRUE	99.467	99.73333333	375	WP_000838029.1	375
Staphylococcus aureus	NZ_CP025495.1	TRUE	100	100	375	WP_000838037.1	375
Staphylococcus aureus	NZ_CP034102.1	TRUE	98.933	99.73333333	375	WP_000838020.1	375
Staphylococcus aureus	NZ_CP033977.1	TRUE	99.467	99.73333333	375	WP_000838029.1	375
Staphylococcus aureus	NZ_CP012120.2	TRUE	100	100	375	WP_000838037.1	375
Staphylococcus aureus	NZ_CP016861.1	TRUE	100	100	375	WP_000838037.1	375
Staphylococcus aureus	NZ_AP018923.1	TRUE	99.467	99.73333333	375	WP_000838033.1	375
Staphylococcus aureus	NZ_CP047854.1	TRUE	99.467	99.73333333	375	WP_000838029.1	375
Staphylococcus aureus	NZ_LT992474.1	TRUE	98.933	99.73333333	375	WP_000838045.1	375
Staphylococcus aureus	NZ_CP026071.1	TRUE	97.6	98.93333333	375	WP_000838047.1	375
Staphylococcus aureus	NZ_CP017090.1	TRUE	99.467	99.73333333	375	WP_000838029.1	375
Staphylococcus aureus subsp. aureus ST772-MRSA-V	NZ_CP010526.1	TRUE	99.2	99.2	375	WP_000838041.1	375
Staphylococcus aureus	NZ_CP047805.1	TRUE	97.6	98.93333333	375	WP_000838047.1	375
Staphylococcus aureus	NZ_AP017320.1	TRUE	99.467	99.73333333	375	WP_000838029.1	375
Staphylococcus aureus	NZ_CP053070.1	TRUE	99.2	100	375	WP_000838027.1	375
Staphylococcus aureus	NZ_CP047859.1	TRUE	99.467	99.73333333	375	WP_000838029.1	375
Staphylococcus aureus	NZ_CP038021.1	TRUE	97.6	98.93333333	375	WP_000838047.1	375
Staphylococcus aureus	NZ_CP073012.1	TRUE	99.467	99.73333333	375	WP_000838039.1	375
Staphylococcus aureus	NZ_LR134271.1	TRUE	98.933	99.73333333	375	WP_000838020.1	375
Staphylococcus aureus	NZ_CP047778.1	TRUE	97.6	98.93333333	375	WP_000838047.1	375
Staphylococcus aureus	NZ_LT992456.1	TRUE	98.933	99.73333333	375	WP_000838045.1	375
Staphylococcus aureus	NZ_LT992463.1	TRUE	98.933	99.73333333	375	WP_000838045.1	375
Staphylococcus aureus	NZ_CP029655.1	TRUE	99.467	99.73333333	375	WP_000838029.1	375
Staphylococcus aureus	NZ_CP039448.1	TRUE	99.2	100	375	WP_000838027.1	375
Staphylococcus aureus	NZ_CP017684.1	TRUE	97.6	98.93333333	375	WP_000838047.1	375
Staphylococcus aureus	NZ_CP017094.1	TRUE	100	100	375	WP_000838037.1	375
Staphylococcus aureus	NZ_CP069470.1	TRUE	100	100	375	WP_000838037.1	375
Staphylococcus aureus	NZ_CP069565.1	TRUE	100	100	375	WP_000838037.1	375
Staphylococcus aureus	NZ_CP031838.1	TRUE	99.467	99.73333333	375	WP_000838039.1	375
Staphylococcus aureus	NZ_CP020467.1	TRUE	97.6	98.93333333	375	WP_000838047.1	375

Staphylococcus aureus	NZ_LT992470.1	TRUE	98.933	99.73333333	375	WP_000838045.1	375
Staphylococcus aureus	NZ_CP047861.1	TRUE	99.467	99.73333333	375	WP_000838029.1	375
Staphylococcus aureus	NZ_CP033114.1	TRUE	97.6	98.93333333	375	WP_000838047.1	375
Staphylococcus aureus	NZ_CP059156.1	TRUE	97.6	98.93333333	375	WP_000838047.1	375
Staphylococcus aureus	NZ_AP023034.1	TRUE	100	100	375	WP_000838037.1	375
Staphylococcus aureus	NZ_CP030136.1	TRUE	99.467	99.73333333	375	WP_000838039.1	375
Staphylococcus aureus CA-347	NC_021554.1	TRUE	98.4	99.46666667	375	WP_000838022.1	375
Staphylococcus aureus	NZ_LS483311.1	TRUE	99.467	99.73333333	375	WP_000838039.1	375
Staphylococcus aureus	NZ_CP011147.1	TRUE	99.14	92.53333333	349	0	0
Staphylococcus aureus	NZ_CP048643.1	TRUE	99.2	99.46666667	375	WP_063653151.1	375
Staphylococcus aureus	NZ_CP047796.1	TRUE	97.6	98.93333333	375	WP_000838047.1	375
Staphylococcus aureus	NZ_CP022290.1	TRUE	100	100	375	WP_000838037.1	375
Staphylococcus aureus	NZ_CP011685.1	TRUE	99.2	99.46666667	375	WP_063653151.1	375
Staphylococcus aureus	NZ_CP044106.1	TRUE	99.2	100	375	WP_000838027.1	375
Staphylococcus aureus	NZ_CP047788.1	TRUE	97.6	98.93333333	375	WP_000838047.1	375
Staphylococcus aureus	NZ_CP012756.1	TRUE	97.867	99.2	375	WP_000838021.1	375
Staphylococcus aureus	NZ_CP062471.1	TRUE	99.2	99.2	375	WP_000838041.1	375
Staphylococcus aureus	NZ_LR134085.1	TRUE	100	100	375	WP_000838037.1	375
Staphylococcus aureus	NZ_CP031673.1	TRUE	100	100	375	WP_000838037.1	375
Staphylococcus aureus subsp. aureus 11819-97	NC_017351.1	TRUE	99.733	100	375	WP_000838031.1	375
Staphylococcus aureus	NZ_CP007659.1	TRUE	98.933	99.46666667	375	WP_000838036.1	375
Staphylococcus aureus	NZ_CP029627.1	TRUE	99.2	99.73333333	375	WP_000838034.1	375
Staphylococcus aureus	NZ_LR134086.1	TRUE	98.933	99.46666667	375	WP_000838036.1	375
Staphylococcus aureus	NZ_LR134090.1	TRUE	98.933	99.73333333	375	WP_000838020.1	375
Staphylococcus aureus	NZ_CP053101.1	TRUE	98.933	99.46666667	375	WP_000838036.1	375
Staphylococcus aureus	NZ_LT992465.1	TRUE	98.933	99.73333333	375	WP_000838045.1	375
Staphylococcus aureus	NZ_CP053640.1	TRUE	99.467	99.73333333	375	WP_000838029.1	375
Staphylococcus aureus	NZ_CP060584.1	TRUE	100	100	375	WP_000838037.1	375
Staphylococcus aureus	NZ_CP047797.1	TRUE	99.467	100	375	WP_000838024.1	375
Staphylococcus aureus	NZ_CP007676.1	TRUE	99.733	99.73333333	375	WP_048520552.1	375
Staphylococcus aureus	NZ_CP038612.1	TRUE	98.933	99.73333333	375	WP_000838020.1	375
Staphylococcus aureus	NZ_CP035671.1	TRUE	98.933	99.46666667	375	WP_000838036.1	375
Staphylococcus aureus	NZ_CP053185.1	TRUE	99.2	100	375	WP_000838027.1	375
Staphylococcus aureus	NZ_CP007672.1	TRUE	100	100	375	WP_000838037.1	375
Staphylococcus aureus	NZ_CP065515.1	TRUE	98.933	99.2	375	WP_197669012.1	375

Staphylococcus aureus	NZ_CP042157.1	TRUE	99.467	99.73333333	375	WP_000838029.1	375
Staphylococcus aureus	NZ_CP047794.1	TRUE	99.467	100	375	WP_000838024.1	375
Staphylococcus aureus	NZ_CP026968.1	TRUE	99.2	99.46666667	375	WP_016170463.1	375
Staphylococcus aureus	NZ_CP031839.1	TRUE	99.467	99.73333333	375	WP_000838039.1	375
Staphylococcus aureus	NZ_CP065194.1	TRUE	98.933	99.73333333	375	WP_000838045.1	375
Staphylococcus aureus	NZ_CP007674.1	TRUE	100	100	375	WP_000838037.1	375
Staphylococcus aureus	NZ_AP024170.1	TRUE	99.467	99.73333333	375	WP_000838029.1	375
Staphylococcus aureus	NZ_LR134093.1	TRUE	97.6	98.93333333	375	WP_000838047.1	375
Staphylococcus aureus	NZ_LR134305.1	TRUE	98.933	99.73333333	375	WP_000838020.1	375
Staphylococcus aureus	NZ_CP018768.1	TRUE	99.467	99.73333333	375	WP_000838029.1	375
Staphylococcus aureus	NZ_LS483324.1	TRUE	99.2	99.73333333	375	WP_000838034.1	375
Staphylococcus aureus	NZ_LR134087.1	TRUE	99.2	99.73333333	375	WP_000838034.1	375
Staphylococcus aureus	NZ_CP026953.1	TRUE	99.2	99.46666667	375	WP_016170463.1	375
Staphylococcus aureus	NZ_CP029649.1	TRUE	99.2	99.46666667	375	WP_000838035.1	375
Staphylococcus aureus	NZ_CP029681.1	TRUE	99.2	99.46666667	375	WP_000838035.1	375
Staphylococcus aureus	NZ_CP026064.1	TRUE	98.933	99.46666667	375	WP_000838036.1	375
Staphylococcus aureus subsp. aureus SA268	NZ_CP006630.1	TRUE	98.933	99.73333333	375	WP_038412493.1	375
Staphylococcus aureus	NZ_CP017091.1	TRUE	98.933	99.73333333	375	WP_000838045.1	375
Staphylococcus aureus	NZ_CP062465.1	TRUE	99.467	99.73333333	375	WP_000838029.1	375
Staphylococcus aureus	NZ_CP042346.1	TRUE	100	100	375	WP_000838037.1	375
Staphylococcus aureus	NZ_CP042348.1	TRUE	100	100	375	WP_000838037.1	375
Staphylococcus aureus	NZ_CP020956.1	TRUE	100	100	375	WP_000838037.1	375
Staphylococcus aureus subsp. aureus ED133	NC_017337.1	TRUE	98.933	99.73333333	375	WP_000838020.1	375
Staphylococcus aureus	NZ_LR134193.1	TRUE	98.933	99.46666667	375	WP_000838036.1	375
Staphylococcus aureus subsp. aureus HO 5096 0412	NC_017763.1	TRUE	98.933	99.46666667	375	WP_000838036.1	375
Staphylococcus aureus	NZ_CP053636.1	TRUE	99.467	99.73333333	375	WP_000838029.1	375
Staphylococcus aureus	NZ_CP020741.1	TRUE	99.2	100	375	WP_000838027.1	375
Staphylococcus aureus	NZ_CP013218.1	TRUE	98.933	99.73333333	375	WP_000838045.1	375
Staphylococcus aureus	NZ_LT009690.1	TRUE	99.467	99.73333333	375	WP_000838029.1	375
Staphylococcus aureus	NZ_CP038850.1	TRUE	98.933	99.46666667	375	WP_000838036.1	375
Staphylococcus aureus	NZ_CP007657.1	TRUE	99.733	100	375	WP_000838038.1	375
Staphylococcus aureus	NZ_CP039164.1	TRUE	99.467	100	375	WP_000838016.1	375
Staphylococcus aureus subsp. aureus NCTC 8325	NZ_LS483365.1	TRUE	100	100	375	WP_000838037.1	375

Staphylococcus aureus	NZ_CP029030.1	TRUE	100	100	375	WP_000838037.1	375
Staphylococcus aureus	NZ_CP029673.1	TRUE	99.467	99.73333333	375	WP_000838029.1	375
Staphylococcus aureus	NZ_LS483350.1	TRUE	100	100	375	WP_000838037.1	375
Staphylococcus aureus	NZ_CP065857.1	TRUE	99.467	99.73333333	375	WP_000838029.1	375
Staphylococcus aureus	NZ_AP020313.1	TRUE	100	100	375	WP_000838037.1	375
Staphylococcus aureus	NZ_CP022897.1	TRUE	99.467	100	375	WP_000838024.1	375
Staphylococcus aureus	NZ_CP022898.1	TRUE	99.467	100	375	WP_000838024.1	375
Staphylococcus aureus subsp. aureus ED98	NC_013450.1	TRUE	99.467	99.73333333	375	WP_000838029.1	375
Staphylococcus aureus	NZ_CP047804.1	TRUE	99.467	99.73333333	375	WP_000838029.1	375
Staphylococcus aureus	NZ_CP031664.1	TRUE	100	100	375	WP_000838037.1	375
Staphylococcus aureus	NZ_CP031661.1	TRUE	100	100	375	WP_000838037.1	375
Staphylococcus aureus	NZ_LR130513.1	TRUE	98.933	99.46666667	375	WP_000838036.1	375
Staphylococcus aureus	NZ_CP047867.1	TRUE	99.467	99.73333333	375	WP_000838029.1	375
Staphylococcus aureus	NZ_CP045472.1	TRUE	99.2	100	375	WP_000838027.1	375
Staphylococcus aureus	NZ_AP020318.1	TRUE	99.733	99.73333333	375	WP_150014941.1	375
Staphylococcus aureus	NZ_CP047799.1	TRUE	97.867	98.93333333	375	WP_000838046.1	375
Staphylococcus aureus 04-02981	NC_017340.1	TRUE	99.467	99.73333333	375	WP_000838029.1	375
Staphylococcus aureus	NZ_CP066488.1	TRUE	99.467	99.73333333	375	WP_000838033.1	375
Staphylococcus aureus subsp. aureus Tager 104	NZ_CP012409.1	TRUE	99.2	100	375	WP_000838023.1	375
Staphylococcus aureus	NZ_CP026957.1	TRUE	99.467	99.73333333	375	WP_000838033.1	375
Staphylococcus aureus subsp. aureus MW2	NC_003923.1	TRUE	99.467	99.73333333	375	WP_000838033.1	375
Staphylococcus aureus	NZ_CP040623.1	TRUE	99.467	99.73333333	375	WP_000838029.1	375
Staphylococcus aureus	NZ_CP040665.1	TRUE	99.467	99.73333333	375	WP_000838029.1	375
Staphylococcus aureus	NZ_CP035791.1	TRUE	99.467	99.73333333	375	WP_000838029.1	375
Staphylococcus aureus	NZ_CP040560.1	TRUE	99.733	100	375	WP_000838038.1	375
Staphylococcus aureus	NZ_CP018205.1	TRUE	100	100	375	WP_000838037.1	375
Staphylococcus aureus	NZ_CP047863.1	TRUE	99.467	99.73333333	375	WP_000838029.1	375
Staphylococcus aureus	NZ_CP042003.1	TRUE	98.933	99.73333333	375	WP_000838045.1	375
Staphylococcus aureus	NZ_CP026073.1	TRUE	99.467	99.73333333	375	WP_000838033.1	375
Staphylococcus aureus	NZ_CP047800.1	TRUE	99.467	99.73333333	375	WP_000838029.1	375
Staphylococcus aureus	NZ_CP047793.1	TRUE	97.333	98.66666667	375	0	0
Staphylococcus aureus	NZ_LS483317.1	TRUE	99.2	100	375	WP_000838023.1	375
Staphylococcus aureus	NZ_CP033999.1	TRUE	99.467	99.73333333	375	WP_000838029.1	375
Staphylococcus aureus	NZ_CP034011.1	TRUE	99.467	99.73333333	375	WP_000838029.1	375

Staphylococcus aureus	NZ_CP034014.1	TRUE	99.467	99.73333333	375	WP_000838029.1	375
Staphylococcus aureus	NZ_CP034008.1	TRUE	99.467	99.73333333	375	WP_000838029.1	375
Staphylococcus aureus	NZ_CP034005.1	TRUE	99.467	99.73333333	375	WP_000838029.1	375
Staphylococcus aureus	NZ_CP034002.1	TRUE	99.467	99.73333333	375	WP_000838029.1	375
Staphylococcus aureus	NZ_CP026066.1	TRUE	97.867	98.93333333	375	WP_000838046.1	375
Staphylococcus aureus	NZ_CP033996.1	TRUE	99.467	99.73333333	375	WP_000838029.1	375
Staphylococcus aureus	NZ_CP033990.1	TRUE	99.467	99.73333333	375	WP_000838029.1	375
Staphylococcus aureus	NZ_CP034259.1	TRUE	99.467	99.73333333	375	WP_000838029.1	375
Staphylococcus aureus	NZ_CP047822.1	TRUE	99.467	99.73333333	375	WP_000838029.1	375
Staphylococcus aureus	NZ_CP010998.1	TRUE	99.2	99.46666667	375	WP_000838035.1	375
Staphylococcus aureus	NZ_CP069799.1	TRUE	99.467	99.73333333	375	WP_000838032.1	375
Staphylococcus aureus subsp. aureus N315	NC_002745.2	TRUE	99.467	99.73333333	375	WP_000838029.1	375
Staphylococcus aureus	NZ_CP026079.1	TRUE	99.467	99.73333333	375	WP_000838029.1	375
Staphylococcus aureus	NZ_CP022717.1	TRUE	99.467	100	375	WP_000838024.1	375
Staphylococcus aureus	NZ_CP022682.1	TRUE	99.467	100	375	WP_000838024.1	375
Staphylococcus aureus	NZ_CP047851.1	TRUE	97.6	98.93333333	375	WP_000838047.1	375
Staphylococcus aureus	NZ_CP022901.1	TRUE	97.6	98.93333333	375	WP_000838047.1	375
Staphylococcus aureus	NZ_CP022900.1	TRUE	97.6	98.93333333	375	WP_000838047.1	375
Staphylococcus aureus	NZ_CP022899.1	TRUE	97.6	98.93333333	375	WP_000838047.1	375
Staphylococcus aureus	NZ_CP019574.1	TRUE	99.467	99.73333333	375	WP_000838033.1	375
Staphylococcus aureus	NZ_CP026076.1	TRUE	100	100	375	WP_000838037.1	375
Staphylococcus aureus	NZ_LT992472.1	TRUE	98.933	99.73333333	375	WP_000838045.1	375
Staphylococcus aureus subsp. aureus JKD6159	NC_017338.1	TRUE	99.467	100	375	WP_000838043.1	375
Staphylococcus aureus	NZ_CP007539.2	TRUE	100	100	375	WP_000838037.1	375
Staphylococcus aureus	NZ_AP018349.1	TRUE	98.933	99.73333333	375	WP_020977111.1	375
Staphylococcus aureus subsp. aureus COL	NC_002951.2	TRUE	100	100	375	WP_000838037.1	375
Staphylococcus aureus	NZ_AP017891.1	TRUE	98.933	99.73333333	375	WP_020977111.1	375
Staphylococcus aureus	NZ_CP019563.1	TRUE	99.467	99.73333333	375	WP_000838029.1	375
Staphylococcus aureus	NZ_AP014921.1	TRUE	100	100	375	WP_000838037.1	375
Staphylococcus aureus	NZ_CP029629.1	TRUE	98.933	99.46666667	375	WP_070046585.1	375
Staphylococcus aureus	NZ_CP022910.1	TRUE	100	100	375	WP_000838037.1	375
Staphylococcus aureus	NZ_CP042107.1	TRUE	99.467	99.73333333	375	WP_000838029.1	375
Staphylococcus aureus	NZ_CP017115.1	TRUE	99.467	99.73333333	375	WP_000838033.1	375
Staphylococcus aureus	NZ_CP047777.1	TRUE	99.467	99.73333333	375	WP_000838029.1	375

Staphylococcus aureus	NZ_CP042043.1	TRUE	99.467	99.73333333	375	WP_000838029.1	375
Staphylococcus aureus	NZ_CP022895.1	TRUE	97.6	98.93333333	375	WP_000838047.1	375
Staphylococcus aureus	NZ_CP022896.1	TRUE	97.6	98.93333333	375	WP_000838047.1	375
Staphylococcus aureus	NZ_AP020311.1	TRUE	100	100	375	WP_000838037.1	375
Staphylococcus aureus	NZ_CP019117.1	TRUE	97.867	98.93333333	375	WP_000838046.1	375
Staphylococcus aureus	NZ_CP047803.1	TRUE	98.4	99.46666667	375	WP_000838022.1	375
Staphylococcus aureus	NZ_CP047789.1	TRUE	100	100	375	WP_000838037.1	375
Staphylococcus aureus	NZ_CP013137.1	TRUE	99.467	100	375	WP_000838024.1	375
Staphylococcus aureus	NZ_CP020019.1	TRUE	98.933	99.73333333	375	WP_000838045.1	375
Staphylococcus aureus subsp. aureus TCH60	NC_017342.1	TRUE	97.867	98.93333333	375	WP_000838046.1	375
Staphylococcus aureus	NZ_CP040233.2	TRUE	98.933	99.73333333	375	WP_000838045.1	375
Staphylococcus aureus	NZ_CP014064.2	TRUE	99.467	99.73333333	375	WP_000838029.1	375
Staphylococcus aureus	NZ_CP026964.1	TRUE	99.467	99.73333333	375	WP_000838033.1	375
Staphylococcus aureus	NZ_CP026960.1	TRUE	100	100	375	WP_000838037.1	375
Staphylococcus aureus subsp. aureus MSSA476	NC_002953.3	TRUE	99.467	99.73333333	375	WP_000838033.1	375
Staphylococcus aureus	NZ_CP012593.1	TRUE	99.467	99.73333333	375	WP_000838029.1	375
Staphylococcus aureus	NZ_CP047839.1	TRUE	97.867	98.93333333	375	WP_000838046.1	375
Staphylococcus aureus	NZ_CP015817.1	TRUE	99.467	99.73333333	375	WP_000838029.1	375
Staphylococcus aureus	NZ_LN831036.1	TRUE	99.733	100	375	WP_000838031.1	375
Staphylococcus aureus	NZ_CP040801.1	TRUE	98.933	99.73333333	375	WP_189912553.1	375
Staphylococcus aureus	NZ_CP015645.1	TRUE	99.467	99.73333333	375	WP_000838029.1	375
Staphylococcus aureus	NZ_CP035005.1	TRUE	98.933	99.73333333	375	WP_000838045.1	375
Staphylococcus aureus	NZ_CP043843.1	TRUE	99.2	99.73333333	375	WP_158173008.1	375
Staphylococcus aureus	NZ_CP053183.1	TRUE	99.2	100	375	WP_000838027.1	375
Staphylococcus aureus	NZ_CP020957.1	TRUE	100	100	375	WP_000838037.1	375
Staphylococcus aureus subsp. aureus M013	NC_016928.2	TRUE	99.2	100	375	WP_000838027.1	375
Staphylococcus aureus	NZ_LT671859.1	TRUE	100	100	375	WP_000838037.1	375
Staphylococcus aureus	NZ_CP038268.1	TRUE	99.467	100	375	WP_000838024.1	375
Staphylococcus aureus	NZ_CP066492.1	TRUE	99.467	99.73333333	375	WP_000838029.1	375
Staphylococcus aureus	NZ_CP029084.1	TRUE	98.4	99.46666667	375	WP_000838022.1	375
Staphylococcus aureus	NZ_CP019594.2	TRUE	98.933	99.73333333	375	WP_000838045.1	375
Staphylococcus aureus	NZ_CP010402.1	TRUE	99.733	100	375	WP_000838031.1	375
Staphylococcus aureus	NZ_CP021907.1	TRUE	97.867	98.93333333	375	WP_000838046.1	375
Staphylococcus aureus	NZ_CP061349.1	TRUE	99.467	99.73333333	375	WP_000838029.1	375

Staphylococcus aureus	NZ_CP047021.1	TRUE	99.2	99.46666667	375	WP_045178081.1	375
Staphylococcus aureus O46	NZ_CP025395.1	TRUE	99.467	100	375	WP_000838024.1	375
Staphylococcus aureus	NZ_CP028165.1	TRUE	98.667	99.73333333	375	WP_046597397.1	375
Staphylococcus aureus	NZ_CP047786.1	TRUE	99.467	99.73333333	375	WP_000838029.1	375
Staphylococcus aureus	NZ_CP030323.1	TRUE	99.2	100	375	WP_000838027.1	375
Staphylococcus aureus	NZ_CP019595.2	TRUE	98.933	99.73333333	375	WP_000838045.1	375
Staphylococcus aureus	NZ_CP019593.2	TRUE	98.933	99.73333333	375	WP_000838045.1	375
Staphylococcus aureus	NZ_LT992466.1	TRUE	98.933	99.73333333	375	WP_000838045.1	375
Staphylococcus aureus	NZ_CP054876.1	TRUE	99.733	100	375	WP_000838031.1	375
Staphylococcus aureus subsp. aureus SA957	NC_022442.1	TRUE	99.2	100	375	WP_000838027.1	375
Staphylococcus aureus	NZ_CP030137.1	TRUE	100	100	375	WP_000838037.1	375
Staphylococcus aureus	NZ_CP029080.1	TRUE	98.4	99.46666667	375	WP_000838022.1	375
Staphylococcus aureus	NZ_CP017682.1	TRUE	99.467	99.73333333	375	WP_000838029.1	375
Staphylococcus aureus	NZ_CP015173.1	TRUE	99.467	99.73333333	375	WP_000838029.1	375
Staphylococcus aureus	NZ_CP031131.1	TRUE	99.2	99.46666667	375	WP_000838035.1	375
Staphylococcus aureus	NZ_CP029652.1	TRUE	98.133	99.2	375	WP_110183618.1	375
Staphylococcus aureus	NZ_CP038183.1	TRUE	99.2	99.46666667	375	WP_000838040.1	375
Staphylococcus aureus	NZ_CP019592.1	TRUE	98.933	99.73333333	375	WP_000838045.1	375
Staphylococcus aureus	NZ_CP045468.1	TRUE	99.2	100	375	WP_000838027.1	375
Staphylococcus aureus	CP073065.1	TRUE	98.933	99.73333333	375	QUD76769.1	375
Staphylococcus aureus	NZ_CP010890.1	TRUE	99.467	99.73333333	375	WP_000838029.1	375
Staphylococcus aureus	NZ_CP040230.2	TRUE	98.933	99.73333333	375	WP_000838045.1	375
Staphylococcus aureus	NZ_CP031779.1	TRUE	99.467	99.73333333	375	WP_000838029.1	375
Staphylococcus aureus 08BA02176	NC_018608.1	TRUE	98.933	99.73333333	375	WP_000838045.1	375
Staphylococcus aureus	NZ_CP039160.1	TRUE	99.2	99.2	375	WP_000838041.1	375
Staphylococcus aureus	NZ_CP042153.1	TRUE	99.467	99.73333333	375	WP_000838029.1	375
Staphylococcus aureus	NZ_CP047847.1	TRUE	99.467	99.73333333	375	WP_000838039.1	375
Staphylococcus aureus	NZ_CP042046.1	TRUE	99.2	99.46666667	375	WP_000838040.1	375
Staphylococcus aureus	NZ_CP047843.1	TRUE	100	100	375	WP_000838037.1	375
Staphylococcus aureus	NZ_CP070986.1	TRUE	99.467	99.73333333	375	WP_000838033.1	375
Staphylococcus aureus	NZ_CP021905.1	TRUE	97.867	98.93333333	375	WP_000838046.1	375
Staphylococcus aureus	NZ_CP009361.1	TRUE	97.867	98.93333333	375	WP_000838046.1	375
Staphylococcus aureus	NZ_CP038270.1	TRUE	98.4	99.73333333	375	WP_000838019.1	375
Staphylococcus aureus	NZ_LN854556.1	TRUE	98.667	99.46666667	375	WP_054189328.1	375
Staphylococcus aureus	NZ_CP032481.1	TRUE	98.4	99.73333333	375	WP_000838019.1	375

Staphylococcus aureus	NZ_CP047833.1	TRUE	98.133	99.2	375	WP_061824077.1	375
Staphylococcus aureus	NZ_CP013616.1	TRUE	98.667	99.73333333	375	WP_031796848.1	375
Staphylococcus aureus	NZ_CP047785.1	TRUE	98.4	99.46666667	375	WP_000838022.1	375
Staphylococcus aureus	NZ_CP034486.1	TRUE	99.467	99.73333333	375	WP_000838033.1	375
Staphylococcus aureus	NZ_AP014942.1	TRUE	99.2	99.73333333	375	WP_000838034.1	375
Staphylococcus aureus	NZ_CP028468.1	TRUE	98.933	99.46666667	375	WP_000838036.1	375
Staphylococcus aureus	NZ_CP028470.1	TRUE	98.933	99.46666667	375	WP_000838036.1	375
Staphylococcus aureus	NZ_CP047835.1	TRUE	99.467	99.73333333	375	WP_000838032.1	375
Staphylococcus aureus	NZ_LS483314.1	TRUE	99.2	99.73333333	375	WP_000838034.1	375
Staphylococcus aureus	NZ_CP021171.1	TRUE	99.467	99.73333333	375	WP_000838029.1	375
Staphylococcus aureus	NZ_CP020020.1	TRUE	99.2	99.73333333	375	WP_000838034.1	375
Staphylococcus aureus	NZ_CP023561.1	TRUE	100	100	375	WP_000838037.1	375
Staphylococcus aureus	NZ_CP032051.1	TRUE	98.933	99.73333333	375	WP_000838020.1	375
Staphylococcus aureus	NZ_CP013182.1	TRUE	99.2	100	375	WP_000838027.1	375
Staphylococcus aureus	NZ_LT615218.1	TRUE	99.467	99.73333333	375	WP_000838029.1	375
Staphylococcus aureus	NZ_AP014652.1	TRUE	99.2	99.46666667	375	WP_000838035.1	375
Staphylococcus aureus	NZ_AP014653.1	TRUE	99.2	99.46666667	375	WP_000838035.1	375
Staphylococcus aureus	NZ_CP023500.1	TRUE	100	100	375	WP_000838037.1	375
Staphylococcus aureus	NZ_CP013619.1	TRUE	98.933	99.73333333	375	WP_000838045.1	375
Staphylococcus aureus	NZ_CP039162.1	TRUE	99.2	99.73333333	375	WP_103152247.1	375
Staphylococcus aureus	NZ_CP047852.1	TRUE	99.467	99.73333333	375	WP_000838032.1	375
Staphylococcus aureus	NZ_CP070983.1	TRUE	99.467	99.73333333	375	WP_000838029.1	375
Staphylococcus aureus	NZ_CP031130.1	TRUE	99.2	99.46666667	375	WP_000838035.1	375
Staphylococcus aureus	NZ_CP022892.1	TRUE	100	100	375	WP_000838037.1	375
Staphylococcus aureus	NZ_CP038460.1	TRUE	98.933	99.73333333	375	WP_000838020.1	375
Staphylococcus aureus	CP070991.1	TRUE	98.667	99.46666667	375	QTY43210.1	375
Staphylococcus aureus	NZ_AP019712.1	TRUE	98.933	99.73333333	375	WP_155978056.1	375
Staphylococcus aureus	NZ_CP047837.1	TRUE	98.4	99.46666667	375	WP_000838022.1	375
Staphylococcus aureus	NZ_CP026063.1	TRUE	99.467	99.73333333	375	WP_000838029.1	375
Staphylococcus aureus	NZ_CP007454.1	TRUE	99.467	99.73333333	375	WP_000838029.1	375
Staphylococcus aureus	NZ_CP038461.1	TRUE	97.6	98.93333333	375	WP_199878690.1	375
Staphylococcus aureus	NZ_CP047820.1	TRUE	100	100	375	WP_000838037.1	375
Staphylococcus aureus	NZ_CP024998.1	TRUE	98.667	99.46666667	375	WP_054189328.1	375
Staphylococcus aureus	NZ_CP022291.1	TRUE	98.667	99.2	375	WP_100185079.1	375
Staphylococcus aureus	NZ_AP020315.1	TRUE	94.933	98.13333333	375	WP_031787305.1	375
Staphylococcus aureus	NZ_CP038819.1	TRUE	99.467	100	375	WP_000838024.1	375

Staphylococcus aureus	NZ_LS483308.1	TRUE	99.733	100	375	WP_000838031.1	375
Staphylococcus aureus	NZ_CP029474.1	TRUE	99.467	99.73333333	375	WP_000838029.1	375
Staphylococcus aureus	NZ_CP016398.1	TRUE	99.2	99.46666667	375	WP_000838035.1	375
Staphylococcus aureus	NZ_CP047792.1	TRUE	98.933	99.73333333	375	WP_124781510.1	375
Staphylococcus aureus subsp. aureus ST228	NC_020532.1	TRUE	99.467	99.73333333	375	WP_000838029.1	375
Staphylococcus aureus	NZ_LR134139.1	TRUE	99.2	99.73333333	375	WP_000838034.1	375
Staphylococcus aureus subsp. aureus ST228	NC_020533.1	TRUE	99.467	99.73333333	375	WP_000838029.1	375
Staphylococcus aureus subsp. aureus ST228	NC_020564.1	TRUE	99.467	99.73333333	375	WP_000838029.1	375
Staphylococcus aureus subsp. aureus ST228	NC_020529.1	TRUE	99.467	99.73333333	375	WP_000838029.1	375
Staphylococcus aureus subsp. aureus ST228	NC_020536.1	TRUE	99.467	99.73333333	375	WP_000838029.1	375
Staphylococcus aureus subsp. aureus ST228	NC_020566.1	TRUE	99.467	99.73333333	375	WP_000838029.1	375
Staphylococcus aureus subsp. aureus ST228	NC_020568.1	TRUE	99.467	99.73333333	375	WP_000838029.1	375
Staphylococcus aureus subsp. aureus ST228	NC_020537.1	TRUE	99.467	99.73333333	375	WP_000838029.1	375
Staphylococcus aureus	NZ_CP017679.1	TRUE	99.2	100	375	WP_000838027.1	375
Staphylococcus aureus	NZ_CP047811.1	TRUE	98.4	99.46666667	375	WP_000838022.1	375
Staphylococcus aureus	NZ_CP040229.2	TRUE	98.667	99.46666667	375	WP_138766948.1	375
Staphylococcus aureus	NZ_CP020959.1	TRUE	100	100	375	WP_000838037.1	375
Staphylococcus aureus subsp. aureus 55/2053	NC_022113.1	TRUE	97.867	98.93333333	375	WP_000838046.1	375
Staphylococcus aureus	NZ_CP068143.1	TRUE	99.2	99.46666667	375	WP_045178081.1	375
Staphylococcus aureus	NZ_CP035003.1	TRUE	98.933	99.73333333	375	WP_000838045.1	375
Staphylococcus aureus	CP071100.1	TRUE	99.467	99.73333333	375	QTY45566.1	375
Staphylococcus aureus	NZ_CP065199.1	TRUE	99.467	99.73333333	375	WP_000838039.1	375
Staphylococcus aureus	NZ_CP047849.1	TRUE	100	100	375	WP_000838037.1	375
Staphylococcus aureus	NZ_CP041037.1	TRUE	99.733	100	375	WP_000838031.1	375
Staphylococcus aureus	NZ_CP047798.1	TRUE	98.4	99.46666667	375	WP_000838022.1	375
Staphylococcus aureus	NZ_CP048431.1	TRUE	99.2	99.73333333	375	WP_000838034.1	375
Staphylococcus aureus	NZ_AP020316.1	TRUE	99.467	99.73333333	375	WP_000838029.1	375
Staphylococcus aureus subsp. aureus CN1	NC_022226.1	TRUE	99.2	99.46666667	375	WP_000838035.1	375

Staphylococcus aureus	NZ_LS483302.1	TRUE	98.667	99.46666667	375		WP_111680192.1	375
Staphylococcus aureus subsp. aureus LGA251	NC_017349.1	TRUE	99.467	100	375		WP_000838024.1	375
Staphylococcus aureus	NZ_LR134091.1	TRUE	99.2	99.73333333	375		WP_000838034.1	375
Staphylococcus aureus	NZ_CP029086.1	TRUE	99.467	99.73333333	375		WP_000838029.1	375
Staphylococcus aureus	NZ_LR133917.1	TRUE	99.467	99.73333333	375		WP_000838032.1	375
Staphylococcus aureus	NZ_CP012974.1	TRUE	99.467	99.73333333	375		WP_000838032.1	375
Staphylococcus aureus	NZ_CP012976.1	TRUE	99.467	99.73333333	375		WP_000838032.1	375
Staphylococcus aureus	NZ_CP031886.1	TRUE	98.4	99.46666667	375		WP_000838022.1	375
Staphylococcus aureus	NZ_CP017680.1	TRUE	99.467	99.73333333	375		WP_000838033.1	375
Staphylococcus aureus	NZ_CP016856.1	TRUE	99.2	99.46666667	375		WP_000838035.1	375
Staphylococcus aureus	NZ_CP047828.1	TRUE	98.4	99.46666667	375		WP_000838022.1	375
Staphylococcus aureus	NZ_CP047790.1	TRUE	98.933	99.46666667	375		WP_160195016.1	375
Staphylococcus aureus	NZ_CP039992.1	TRUE	99.2	99.2	375		WP_000838041.1	375
Staphylococcus aureus	NZ_CP047780.1	TRUE	99.467	99.73333333	375		WP_000838029.1	375
Staphylococcus aureus	NZ_CP038229.1	TRUE	99.2	99.46666667	375		WP_045178081.1	375
Staphylococcus aureus	NZ_CP021105.1	TRUE	99.467	99.73333333	375		WP_000838029.1	375
Staphylococcus aureus	NZ_CP047826.1	TRUE	99.2	99.46666667	375		WP_000838040.1	375
Staphylococcus aureus	NZ_CP050691.1	TRUE	99.2	99.46666667	375		WP_167780421.1	375
Staphylococcus aureus	NZ_CP015646.1	TRUE	99.2	99.46666667	375		WP_000838040.1	375
Staphylococcus aureus RF122	NC_007622.1	TRUE	99.2	100	375		WP_000838023.1	375
Staphylococcus aureus	NZ_CP050690.1	TRUE	100	100	375		WP_000838037.1	375
Staphylococcus aureus	NZ_CP047817.1	TRUE	99.467	99.73333333	375		WP_000838029.1	375
Staphylococcus aureus	NZ_CP026070.1	TRUE	100	100	375		WP_000838037.1	375
Staphylococcus aureus	NZ_CP047841.1	TRUE	99.467	99.73333333	375		WP_000838039.1	375
Staphylococcus aureus	NZ_CP031265.1	TRUE	99.467	99.73333333	375		WP_000838033.1	375
Staphylococcus aureus subsp. aureus 6850	NC_022222.1	TRUE	98.933	99.73333333	375		WP_020977111.1	375
Staphylococcus aureus	NZ_CP047783.1	TRUE	99.2	100	375		WP_000838027.1	375
Staphylococcus aureus	NZ_AP018922.1	TRUE	99.467	99.73333333	375		WP_000838029.1	375
Staphylococcus aureus	NZ_LR130511.1	TRUE	99.467	99.73333333	375		WP_000838029.1	375
Staphylococcus aureus	NZ_CP047813.1	TRUE	99.467	99.73333333	375		WP_000838033.1	375
Staphylococcus aureus	NZ_CP034098.1	TRUE	97.867	98.93333333	375		WP_000838046.1	375
Staphylococcus aureus	NZ_CP020714.1	TRUE	99.467	99.73333333	375		WP_000838039.1	375
Staphylococcus aureus	NZ_CP022607.1	TRUE	99.2	99.46666667	375		WP_000838035.1	375
Staphylococcus aureus	NZ_CP022718.1	TRUE	100	100	375		WP_000838037.1	375

Staphylococcus aureus	NZ_CP022720.1	TRUE	100	100	375	WP_000838037.1	375
Staphylococcus aureus	NZ_LT996891.1	TRUE	100	100	375	WP_000838037.1	375
Staphylococcus aureus	NZ_LT996890.1	TRUE	100	100	375	WP_000838037.1	375
Staphylococcus aureus	NZ_LT996889.1	TRUE	100	100	375	WP_000838037.1	375
Staphylococcus aureus	NZ_CP046476.1	TRUE	99.2	99.73333333	375	WP_000838034.1	375
Staphylococcus aureus	NZ_CP047830.1	TRUE	100	100	375	WP_000838037.1	375
Staphylococcus aureus	NZ_CP017677.1	TRUE	99.467	99.73333333	375	WP_000838029.1	375
Staphylococcus aureus	NZ_LT992436.1	TRUE	98.4	99.46666667	375	WP_000838022.1	375
Staphylococcus aureus	NZ_LT992435.1	TRUE	98.4	99.46666667	375	WP_000838022.1	375
Staphylococcus aureus	NZ_LT992434.1	TRUE	98.4	99.46666667	375	WP_000838022.1	375
Staphylococcus aureus subsp. aureus ECT-R 2	NC_017343.1	TRUE	99.467	99.73333333	375	WP_000838029.1	375
Staphylococcus aureus	NZ_CP020713.1	TRUE	99.467	99.73333333	375	WP_000838039.1	375
Staphylococcus aureus	NZ_AP017922.1	TRUE	99.2	99.73333333	375	WP_029549772.1	375
Staphylococcus aureus subsp. aureus SA40	NC_022443.1	TRUE	99.2	100	375	WP_000838027.1	375
Staphylococcus aureus	NZ_CP045866.1	TRUE	99.467	99.73333333	375	WP_000838029.1	375
Staphylococcus aureus	NZ_CP012972.1	TRUE	100	100	375	WP_000838037.1	375
Staphylococcus aureus	NZ_CP012970.1	TRUE	100	100	375	WP_000838037.1	375
Staphylococcus aureus	NZ_CP011528.1	TRUE	99.467	99.73333333	375	WP_000838039.1	375
Staphylococcus aureus	NZ_LR130509.1	TRUE	99.467	99.73333333	375	WP_000838033.1	375
Staphylococcus aureus	NZ_CP019591.2	TRUE	98.933	99.73333333	375	WP_000838045.1	375
Staphylococcus aureus	NZ_CP047845.1	TRUE	98.4	99.46666667	375	WP_000838022.1	375
Staphylococcus aureus	NZ_CP028189.1	TRUE	99.467	99.73333333	375	WP_000838039.1	375
Staphylococcus aureus	CP071102.1	TRUE	99.733	100	375	QTY48245.1	375
Staphylococcus aureus	NZ_CP013132.1	TRUE	99.467	99.73333333	375	WP_000838033.1	375
Staphylococcus aureus	NZ_CP020656.1	TRUE	99.467	99.73333333	375	WP_000838039.1	375
Staphylococcus aureus	NZ_LS483310.1	TRUE	99.2	99.73333333	375	WP_000838034.1	375
Staphylococcus aureus	NZ_CP034349.1	TRUE	97.867	98.93333333	375	WP_000838046.1	375
Staphylococcus aureus	NZ_CP009828.1	TRUE	99.2	100	375	WP_000838027.1	375
Staphylococcus aureus	NZ_CP047815.1	TRUE	99.467	99.73333333	375	WP_000838029.1	375
Staphylococcus aureus	NZ_CP053634.1	TRUE	99.467	99.73333333	375	WP_000838029.1	375
Staphylococcus aureus	NZ_CP020354.1	TRUE	99.467	99.73333333	375	WP_000838029.1	375
Staphylococcus aureus	NZ_CP047795.1	TRUE	98.4	99.46666667	375	WP_000838022.1	375
Staphylococcus aureus	NZ_LS483300.1	TRUE	98.933	100	375	WP_111725095.1	375
Staphylococcus aureus	NZ_CP047807.1	TRUE	99.467	99.73333333	375	WP_000838039.1	375

Staphylococcus aureus	NZ_CP026962.1	TRUE	100	100	375	WP_000838037.1	375
Staphylococcus aureus	NZ_CP026080.1	TRUE	100	100	375	WP_000838037.1	375
Staphylococcus aureus	NZ_CP026068.1	TRUE	100	100	375	WP_000838037.1	375
Staphylococcus aureus	NZ_CP026077.1	TRUE	100	100	375	WP_000838037.1	375
Staphylococcus aureus	NZ_CP038269.1	TRUE	99.2	100	375	WP_000838027.1	375
Staphylococcus aureus subsp. aureus VC40	NC_016912.1	TRUE	100	100	375	WP_000838037.1	375
Staphylococcus aureus	NZ_CP029671.1	TRUE	99.467	99.73333333	375	WP_000838029.1	375
Staphylococcus aureus	NZ_CP047784.1	TRUE	99.467	99.73333333	375	WP_000838033.1	375
Staphylococcus aureus	NZ_CP012979.1	TRUE	100	100	375	WP_000838037.1	375
Staphylococcus aureus	NZ_CP012978.1	TRUE	100	100	375	WP_000838037.1	375
Staphylococcus aureus	NZ_CP017685.1	TRUE	98.4	99.46666667	375	WP_000838022.1	375
Staphylococcus aureus	NZ_CP047787.1	TRUE	98.4	99.46666667	375	WP_000838022.1	375
Staphylococcus aureus	CP071104.1	TRUE	97.6	98.93333333	375	QTY50500.1	375
Staphylococcus aureus	NZ_CP014791.1	TRUE	98.4	99.46666667	375	WP_000838022.1	375
Staphylococcus aureus	NZ_CP043300.1	TRUE	99.2	99.46666667	375	WP_000838042.1	375
Staphylococcus aureus	NZ_CP060629.1	TRUE	98.933	99.46666667	375	WP_000838036.1	375
Staphylococcus aureus	NZ_CP060631.1	TRUE	100	100	375	WP_000838037.1	375
Staphylococcus aureus	NZ_CP021352.1	TRUE	97.6	98.93333333	375	WP_000838047.1	375
Staphylococcus aureus	NZ_CP035415.1	TRUE	99.467	99.73333333	375	WP_000838029.1	375
Staphylococcus aureus	NZ_CP071374.1	TRUE	100	100	375	WP_000838037.1	375
Staphylococcus aureus	CP033086.1	TRUE	99.2	100	375	QHG79335.1	375
Staphylococcus aureus	NZ_CP060627.1	TRUE	98.667	99.2	375	WP_187417727.1	375
Staphylococcus aureus	NZ_CP071355.1	TRUE	100	100	375	WP_000838037.1	375
Staphylococcus aureus	NZ_CP060621.1	TRUE	99.467	99.73333333	375	WP_000838029.1	375
Staphylococcus aureus	CP033085.1	TRUE	99.2	100	375	QHG76317.1	375
Staphylococcus aureus	NZ_CP021353.1	TRUE	99.467	99.73333333	375	WP_000838029.1	375
Staphylococcus aureus	NZ_CP060614.1	TRUE	98.933	99.46666667	375	WP_000838036.1	375
Staphylococcus aureus	NZ_CP060615.1	TRUE	98.933	99.46666667	375	WP_000838036.1	375
Staphylococcus aureus	NZ_CP060623.1	TRUE	99.462	98.93333333	372	WP_025175628.1	375
Staphylococcus aureus	NZ_CP060625.1	TRUE	99.462	98.93333333	372	WP_025175628.1	375
Staphylococcus aureus	NZ_CP060626.1	TRUE	99.462	98.93333333	372	WP_025175628.1	375
Staphylococcus aureus	NZ_CP071348.1	TRUE	100	100	375	WP_000838037.1	375
Staphylococcus aureus	NZ_CP060624.1	TRUE	99.462	98.93333333	372	WP_025175628.1	375
Staphylococcus aureus	CP071347.1	TRUE	100	100	375	QSU32015.1	375
Staphylococcus aureus	CP071342.1	TRUE	100	100	375	QSU18981.1	375

Staphylococcus aureus	CP071343.1	TRUE	100	100	375	QSU21645.1	375
Staphylococcus aureus	NZ_CP071340.1	TRUE	100	100	375	WP_000838037.1	375
Staphylococcus aureus	CP071353.1	TRUE	100	100	375	QSU47665.1	375
Staphylococcus aureus	NZ_CP033681.1	TRUE	100	100	375	WP_000838037.1	375
Staphylococcus aureus	NZ_CP021350.1	TRUE	99.467	99.73333333	375	WP_000838029.1	375
Staphylococcus aureus	NZ_CP060616.1	TRUE	98.933	99.46666667	375	WP_000838036.1	375
Staphylococcus aureus	NZ_CP060617.1	TRUE	98.933	99.46666667	375	WP_000838036.1	375
Staphylococcus aureus	NZ_CP060628.1	TRUE	98.667	99.2	375	WP_187417727.1	375
Staphylococcus aureus	NZ_CP071352.1	TRUE	100	100	375	WP_000838037.1	375
Staphylococcus aureus	NZ_CP060622.1	TRUE	99.467	99.73333333	375	WP_000838029.1	375
Staphylococcus aureus	NZ_CP021349.1	TRUE	99.467	99.73333333	375	WP_000838029.1	375
Staphylococcus aureus	NZ_CP021351.1	TRUE	99.467	99.73333333	375	WP_000838029.1	375
Staphylococcus aureus	CP071364.1	TRUE	100	100	375	QSU80603.1	375
Staphylococcus aureus	CP071371.1	TRUE	100	100	375	QSU98618.1	375
Staphylococcus aureus	NZ_CP071361.1	TRUE	100	100	375	WP_000838037.1	375
Staphylococcus aureus	NZ_CP060613.1	TRUE	98.4	99.46666667	375	WP_000838022.1	375
Staphylococcus aureus	CP071363.1	TRUE	100	100	375	QSU77927.1	375
Staphylococcus aureus	CP071332.1	TRUE	100	100	375	QST93088.1	375
Staphylococcus aureus	CP071366.1	TRUE	100	100	375	QSU88327.1	375
Staphylococcus aureus	CP071360.1	TRUE	100	100	375	QSU65887.1	375
Staphylococcus aureus	NZ_CP071336.1	TRUE	100	100	375	WP_000838037.1	375
Staphylococcus aureus	NZ_CP071362.1	TRUE	100	100	375	WP_000838037.1	375
Staphylococcus aureus	NZ_CP071356.1	TRUE	100	100	375	WP_000838037.1	375
Staphylococcus aureus	NZ_CP071354.1	TRUE	100	100	375	WP_000838037.1	375
Staphylococcus aureus	CP071358.1	TRUE	100	100	375	QSU60755.1	375
Staphylococcus aureus	CP071367.1	TRUE	100	100	375	QSU85730.1	375
Staphylococcus aureus	CP071368.1	TRUE	100	100	375	QSU90917.1	375
Staphylococcus aureus	NZ_CP071370.1	TRUE	100	100	375	WP_000838037.1	375
Staphylococcus aureus	NZ_CP039759.1	TRUE	99.467	99.73333333	375	WP_000838039.1	375
Staphylococcus aureus	CP071333.1	TRUE	100	100	375	QST95692.1	375
Staphylococcus aureus	CP071365.1	TRUE	100	100	375	QSU83243.1	375
Staphylococcus aureus	CP071334.1	TRUE	100	100	375	QST98297.1	375
Staphylococcus aureus	CP071345.1	TRUE	100	100	375	QSU26755.1	375
Staphylococcus aureus	NZ_CP071341.1	TRUE	100	100	375	WP_000838037.1	375
Staphylococcus aureus	CP071339.1	TRUE	100	100	375	QSU11144.1	375
Staphylococcus aureus	CP071373.1	TRUE	100	100	375	QSV03759.1	375

Staphylococcus aureus	CP071351.1	TRUE	100	100	375	QSU37257.1	375
Staphylococcus aureus	CP071346.1	TRUE	100	100	375	QSU29354.1	375
Staphylococcus aureus	CP071372.1	TRUE	100	100	375	QSV01164.1	375
Staphylococcus aureus	NZ_CP071337.1	TRUE	99.733	99.73333333	375	0	0
Staphylococcus aureus	CP071349.1	TRUE	100	100	375	QSU39872.1	375
Staphylococcus aureus	CP071369.1	TRUE	100	100	375	QSU92433.1	375
Staphylococcus aureus	CP071357.1	TRUE	100	100	375	QSU58214.1	375
Staphylococcus aureus	CP071359.1	TRUE	100	100	375	QSU63286.1	375
Staphylococcus aureus	NZ_CP071350.1	TRUE	100	100	375	WP_000838037.1	375
Staphylococcus aureus	NZ_CP071338.1	TRUE	100	100	375	WP_000838037.1	375
Staphylococcus aureus	NZ_CP071335.1	TRUE	100	100	375	WP_000838037.1	375
Staphylococcus aureus	CP071344.1	TRUE	100	100	375	QSU24203.1	375
Staphylococcus aureus	NZ_AP015012.1	TRUE	99.467	99.73333333	375	WP_000838033.1	375
Staphylococcus aureus subsp. aureus 71193	NC_017673.1	TRUE	98.933	99.73333333	375	WP_000838045.1	375
Staphylococcus aureus	CP033117.1	TRUE	99.467	99.73333333	375	QFZ04281.1	375
Staphylococcus aureus	NZ_CP023560.1	TRUE	100	100	375	WP_000838037.1	375
Staphylococcus aureus	NZ_LT963437.1	TRUE	100	100	375	WP_000838037.1	375
Staphylococcus aureus	NZ_CP017804.1	TRUE	99.467	99.73333333	375	WP_000838029.1	375
Staphylococcus aureus	NZ_CP017807.1	TRUE	99.467	99.73333333	375	WP_000838029.1	375
Staphylococcus aureus	NZ_CP063802.1	TRUE	98.667	99.2	375	WP_124374963.1	375
Staphylococcus aureus	NZ_CP020553.1	TRUE	99.467	99.73333333	375	WP_000838029.1	375
Staphylococcus aureus	CP043917.1	TRUE	98.667	99.46666667	375	QER25112.1	375
Staphylococcus aureus	NZ_CP020544.1	TRUE	99.467	99.73333333	375	WP_000838029.1	375
Staphylococcus aureus	NZ_CP043918.1	TRUE	98.667	99.46666667	375	WP_054189328.1	375
Staphylococcus aureus	NZ_CP043921.1	TRUE	98.667	99.46666667	375	WP_054189328.1	375
Staphylococcus aureus	NZ_CP063686.1	TRUE	99.733	100	375	WP_000838031.1	375
Staphylococcus aureus	NZ_CP063801.1	TRUE	99.733	100	375	WP_000838031.1	375
Staphylococcus aureus	CP043915.1	TRUE	98.667	99.46666667	375	QER15468.1	375
Staphylococcus aureus	NZ_CP043914.1	TRUE	100	100	375	WP_000838037.1	375
Staphylococcus aureus	CP043911.1	TRUE	98.667	99.46666667	375	QER02814.1	375
Staphylococcus aureus subsp. aureus USA300	NZ_CP027476.1	TRUE	100	100	375	WP_000838037.1	375
Staphylococcus aureus M1	NC_021059.1	TRUE	100	100	375	WP_000838037.1	375
Staphylococcus aureus	NZ_CP043922.1	TRUE	99.467	99.73333333	375	WP_000838029.1	375
Staphylococcus aureus	NZ_CP043923.1	TRUE	99.467	99.73333333	375	WP_000838029.1	375

Staphylococcus aureus	NZ_CP043916.1	TRUE	99.467	99.73333333	375	WP_000838029.1	375
Staphylococcus aureus	AP018376.1	TRUE	99.2	99.73333333	375	BBD10937.1	375
Staphylococcus aureus	NZ_CP032161.1	TRUE	98.933	99.46666667	375	WP_000838036.1	375
Staphylococcus aureus	NZ_CP043913.1	TRUE	97.867	98.93333333	375	WP_000838046.1	375
Staphylococcus aureus	NZ_CP039848.1	TRUE	99.2	99.73333333	375	WP_000838034.1	375
Staphylococcus aureus	NZ_CP021141.1	TRUE	97.867	98.93333333	375	WP_000838046.1	375
Staphylococcus aureus	NZ_CP033390.1	TRUE	98.933	99.46666667	375	WP_072353872.1	375
Staphylococcus aureus	NZ_CP060608.1	TRUE	99.2	99.46666667	375	WP_045178081.1	375
Staphylococcus aureus	NZ_CP060598.1	TRUE	99.2	99.46666667	375	WP_000838040.1	375
Staphylococcus aureus	NZ_CP043912.1	TRUE	98.667	99.46666667	375	WP_054189328.1	375
Staphylococcus aureus	NZ_CP060609.1	TRUE	99.2	99.46666667	375	WP_045178081.1	375
Staphylococcus aureus	NZ_CP060599.1	TRUE	99.2	99.46666667	375	WP_000838040.1	375
Staphylococcus aureus	NZ_CP060603.1	TRUE	100	100	375	WP_000838037.1	375
Staphylococcus aureus	NZ_CP060604.1	TRUE	100	100	375	WP_000838037.1	375
Staphylococcus aureus	NZ_CP060610.1	TRUE	99.2	99.46666667	375	WP_045178081.1	375
Staphylococcus aureus	NZ_CP060597.1	TRUE	99.2	99.73333333	375	WP_029549772.1	375
Staphylococcus aureus	NZ_CP060602.1	TRUE	100	100	375	WP_000838037.1	375
Staphylococcus aureus	NZ_CP060607.1	TRUE	99.2	99.46666667	375	WP_045178081.1	375
Staphylococcus aureus	NZ_CP060606.1	TRUE	99.2	99.46666667	375	WP_045178081.1	375
Staphylococcus aureus	NZ_CP060605.1	TRUE	99.467	99.46666667	375	WP_061838860.1	375
Staphylococcus aureus	NZ_CP043920.1	TRUE	99.467	99.73333333	375	WP_000838033.1	375
Staphylococcus aureus	NZ_CP060596.1	TRUE	99.2	99.73333333	375	WP_029549772.1	375
Staphylococcus aureus	NZ_CP060601.1	TRUE	98.4	99.46666667	375	WP_000838022.1	375
Staphylococcus aureus	NZ_CP060600.1	TRUE	98.4	99.46666667	375	WP_000838022.1	375
Staphylococcus aureus	NZ_CP060611.1	TRUE	97.6	98.93333333	375	WP_000838047.1	375
Staphylococcus aureus	NZ_CP043919.1	TRUE	100	100	375	WP_000838037.1	375
Staphylococcus aureus	NZ_CP027101.1	TRUE	100	100	375	WP_000838037.1	375
Staphylococcus aureus	CP042110.1	TRUE	98.933	99.73333333	375	QDS62728.1	375
Staphylococcus aureus	NZ_CP042008.1	TRUE	99.2	99.46666667	375	WP_000838040.1	375
Staphylococcus aureus	NZ_CP042048.1	TRUE	99.2	99.46666667	375	WP_000838040.1	375
Staphylococcus aureus	NZ_CP042081.1	TRUE	99.467	99.73333333	375	WP_000838039.1	375
Staphylococcus aureus subsp. aureus MN8	NZ_CM000952.1	TRUE	97.6	98.93333333	375	WP_000838047.1	375
Staphylococcus aureus	CP062279.1	TRUE	98.667	99.2	375	QOJ76359.1	374
Staphylococcus aureus	CP013959.1	TRUE	99.733	100	375	AND44192.1	375
Staphylococcus aureus	CP007447.1	TRUE	100	100	375	AID39392.1	375

Staphylococcus aureus	CP045442.1	TRUE	99.467	99.73333333	375	QLI02783.1	375
Staphylococcus aureus	CP045439.1	TRUE	99.467	99.73333333	375	QLH97569.1	375
Staphylococcus aureus	CP051191.1	TRUE	99.2	99.46666667	375	QNU46716.1	375
Staphylococcus aureus	CP035670.1	TRUE	99.2	99.2	375	QBB15788.1	375
Staphylococcus aureus	CP051482.1	TRUE	99.467	99.73333333	375	QNU53987.1	375
Staphylococcus aureus	LT699704.1	TRUE	98.933	99.73333333	375	#N/A	#N/A
Staphylococcus aureus	CP063990.1	TRUE	99.467	99.73333333	375	#N/A	#N/A
Staphylococcus aureus	CP045474.1	TRUE	99.467	99.73333333	375	QLH59948.1	375
Staphylococcus aureus	CP026646.1	TRUE	99.467	99.73333333	375	QBP95739.1	375
Staphylococcus aureus	CP045441.1	TRUE	99.467	99.73333333	375	QLI00237.1	375
Staphylococcus aureus	CP051483.1	TRUE	99.467	99.73333333	375	QNU56188.1	375
Staphylococcus aureus	CP051479.1	TRUE	99.2	99.46666667	375	QNU49196.1	375
Staphylococcus aureus	LR134268.1	TRUE	97.867	99.2	375	VED72655.1	375
Staphylococcus aureus	CP033506.1	TRUE	99.2	99.73333333	375	AYU99176.1	375
Staphylococcus aureus	CP033505.1	TRUE	99.2	99.73333333	375	AYV01597.1	375
Staphylococcus aureus	CP070989.1	TRUE	100	100	375	QSF66864.1	375
Staphylococcus aureus	CP051484.1	TRUE	98.667	99.46666667	375	QNU58939.1	375
Staphylococcus aureus	CP041746.1	TRUE	99.2	99.73333333	375	QHG58930.1	375
Staphylococcus aureus	CP051165.1	TRUE	98.667	99.46666667	375	QNU51541.1	375
Staphylococcus aureus O11	CP024649.1	TRUE	98.4	99.73333333	375	ATV03674.1	375
Staphylococcus aureus	CP027486.1	TRUE	99.467	99.73333333	375	AVO68414.1	375
Staphylococcus aureus	LR134084.1	TRUE	99.467	100	375	VDZ11723.1	375
Staphylococcus aureus	CP041633.1	TRUE	98.933	99.73333333	375	QOG15275.1	375
Staphylococcus aureus	LR134088.1	TRUE	99.2	100	375	VDZ34732.1	375
Staphylococcus aureus	CP060630.1	TRUE	100	100	375	#N/A	#N/A
Staphylococcus aureus	CP060619.1	TRUE	99.2	99.2	375	#N/A	#N/A
Staphylococcus aureus	CP060618.1	TRUE	99.2	99.2	375	#N/A	#N/A
Staphylococcus aureus	CP060620.1	TRUE	99.2	99.2	375	#N/A	#N/A
Staphylococcus aureus	CP060612.1	TRUE	99.363	83.46666667	314	#N/A	#N/A
Staphylococcus aureus	CP032160.1	TRUE	98.4	99.46666667	375	QOE85873.1	375
Staphylococcus aureus	CP032468.1	TRUE	100	100	375	QOE20949.1	375
Staphylococcus aureus	CP010943.1	TRUE	99.2	99.2	375	AJP26698.1	375
Staphylococcus aureus	CP010941.1	TRUE	98.667	99.46666667	375	AJP22103.1	375
Staphylococcus aureus	CP010944.1	TRUE	99.2	99.2	375	AJP29346.1	375
Staphylococcus aureus	CP010952.1	TRUE	99.467	100	375	AJP65343.1	375
Staphylococcus aureus	CP010940.1	TRUE	98.933	99.46666667	375	AJP19349.1	375

Table S4. The distribution of OfrA orthologues in Firmicutes.

**Organism Name**	**Chromosome**	**presence**	**aa_identity**	**aa_similarity**	**alignment_length**	**protein_id**	**protein_length**
Staphylococcus aureus subsp. aureus NCTC 8325	NC_007795.1	TRUE	100	100	375	YP_499446.1	375
Staphylococcus epidermidis	NZ_CP035288.1	TRUE	81.333	92.26666667	375	WP_002439060.1	375
Lactococcus lactis	NZ_CP066300.1	TRUE	38.259	56	379	WP_098393323.1	391
Bacillus cereus B4264	NC_011725.1	FALSE	NA	NA	NA	NA	NA
Clostridium perfringens ATCC 13124	NC_008261.1	FALSE	NA	NA	NA	NA	NA
Listeria monocytogenes EGD-e	NC_003210.1	FALSE	NA	NA	NA	NA	NA
Streptococcus pyogenes	NZ_CP010450.1	FALSE	NA	NA	NA	NA	NA
Bacillus anthracis str. 'Ames Ancestor'	NC_007530.2	FALSE	NA	NA	NA	NA	NA
Streptococcus agalactiae	NZ_LR134512.1	FALSE	NA	NA	NA	NA	NA
Streptococcus suis SC84	NC_012924.1	FALSE	NA	NA	NA	NA	NA
Bacillus licheniformis DSM 13 = ATCC 14580	NC_006270.3	TRUE	44.058	57.6	345	WP_003185542.1	364
Streptococcus thermophilus JIM 8232	NC_017581.1	FALSE	NA	NA	NA	NA	NA
Limosilactobacillus reuteri	NZ_CP045605.1	FALSE	NA	NA	NA	NA	NA
Bacillus thuringiensis Bt407	NZ_CM000747.1	FALSE	NA	NA	NA	NA	NA
Lactobacillus delbrueckii subsp. delbrueckii	NZ_AP019750.1	FALSE	NA	NA	NA	NA	NA
Clostridioides difficile	NZ_CP019870.1	FALSE	NA	NA	NA	NA	NA
Desulfitobacterium hafniense DCB-2	NC_011830.1	FALSE	NA	NA	NA	NA	NA
Oenococcus oeni	NZ_CP014324.1	TRUE	41.667	58.13333333	360	WP_032805434.1	372
Lactobacillus casei DSM 20011 = JCM 1134 = ATCC 393	NZ_AP012544.1	TRUE	37.143	51.2	350	WP_025013720.1	381
Bacillus subtilis subsp. subtilis str. 168	NC_000964.3	TRUE	47.467	65.86666667	375	NP_390301.1	372
Lactobacillus helveticus	NZ_CP015444.1	FALSE	NA	NA	NA	NA	NA
Faecalibacterium prausnitzii	NZ_CP030777.1	FALSE	NA	NA	NA	NA	NA
Lactococcus garvieae	NZ_CP065637.1	FALSE	NA	NA	NA	NA	NA
Clostridium kluyveri NBRC 12016	NC_011837.1	FALSE	NA	NA	NA	NA	NA
Clostridium botulinum	NZ_CP028842.1	FALSE	NA	NA	NA	NA	NA
Streptococcus dysgalactiae	NZ_LR594046.1	TRUE	35.127	52.26666667	353	WP_138125660.1	277
Hungateiclostridium thermocellum DSM 2360	NZ_CP016502.1	FALSE	NA	NA	NA	NA	NA
Bacillus amyloliquefaciens	NZ_CP053376.1	TRUE	47.09	67.2	378	WP_024085589.1	372
Streptococcus mutans	NZ_CP013237.1	FALSE	NA	NA	NA	NA	NA

Enterococcus faecium ATCC 8459 = NRRL B-2354	NC_020207.1	TRUE	41.228	56.26666667	342	WP_002294672.1	363
Bacillus mycoides	NZ_CM000719.1	FALSE	NA	NA	NA	NA	NA
Streptococcus equi subsp. zooepidemicus	NZ_CP065061.1	FALSE	NA	NA	NA	NA	NA
Enterococcus casseliflavus EC20	NC_020995.1	TRUE	38.525	57.06666667	366	WP_015509351.1	370
Erysipelatoclostridium ramosum	NZ_CP068170.1	FALSE	NA	NA	NA	NA	NA
Coprococcus comes	NZ_CP070062.1	FALSE	NA	NA	NA	NA	NA
Clostridium sporogenes	NZ_CP011663.1	FALSE	NA	NA	NA	NA	NA
Parvimonas micra	NZ_CP009761.1	FALSE	NA	NA	NA	NA	NA
[Clostridium] scindens ATCC 35704	NZ_CP036170.1	FALSE	NA	NA	NA	NA	NA
[Ruminococcus] gnavus ATCC 29149	NZ_CP027002.1	FALSE	NA	NA	NA	NA	NA
Anaerostipes caccae	NZ_CP036345.1	FALSE	NA	NA	NA	NA	NA
Enterocloster bolteae	NZ_CP022464.2	FALSE	NA	NA	NA	NA	NA
Ruminococcus albus 7 = DSM 20455	NC_014833.1	FALSE	NA	NA	NA	NA	NA
Streptococcus gordonii	NZ_LR594049.1	FALSE	NA	NA	NA	NA	NA
Clostridium acetobutylicum DSM 1731	NC_015687.1	FALSE	NA	NA	NA	NA	NA
Lactobacillus gasseri ATCC 33323 = JCM 1131	NC_008530.1	FALSE	NA	NA	NA	NA	NA
Bacillus halodurans C-125	NC_002570.2	FALSE	NA	NA	NA	NA	NA
Carboxydothermus hydrogenoformans Z-2901	NC_007503.1	FALSE	NA	NA	NA	NA	NA
Oceanobacillus iheyensis HTE831	NC_004193.1	TRUE	44.149	63.73333333	376	WP_011067711.1	373
Leuconostoc mesenteroides	NZ_CP028251.1	TRUE	41.57	54.93333333	344	WP_160215213.1	396
Lactobacillus acidophilus La-14	NC_021181.2	TRUE	38	44.8	300	WP_003548097.1	306
Lactiplantibacillus plantarum	NZ_CP030105.1	TRUE	36.443	54.66666667	343	WP_022638078.1	369
Pediococcus pentosaceus ATCC 25745	NC_008525.1	FALSE	NA	NA	NA	NA	NA
Levilactobacillus brevis	NZ_LS483405.1	TRUE	39.833	55.2	359	WP_021742049.1	396
Moorella thermoacetica	NZ_CP017237.1	FALSE	NA	NA	NA	NA	NA
Exiguobacterium sibiricum 255-15	NC_010556.1	TRUE	39.894	60.8	376	WP_012371710.1	371
Staphylococcus haemolyticus	NZ_CP013911.1	TRUE	77.867	90.13333333	375	WP_011276261.1	375
Syntrophomonas wolfei subsp. wolfei str. Goettingen G311	NC_008346.1	FALSE	NA	NA	NA	NA	NA
[Eubacterium] eligens ATCC 27750	NC_012778.1	FALSE	NA	NA	NA	NA	NA
Lactobacillus salivarius str. Ren	NZ_CP011403.1	TRUE	36.508	52	378	WP_047035862.1	382

Desulfotomaculum reducens MI-1	NC_009253.1	FALSE	NA	NA	NA	NA	NA
Heliobacterium modesticaldum Ice1	NC_010337.2	FALSE	NA	NA	NA	NA	NA
Listeria ivanovii subsp. ivanovii	NZ_CP009577.1	FALSE	NA	NA	NA	NA	NA
Listeria seeligeri serovar 1/2b str. SLCC3954	NC_013891.1	FALSE	NA	NA	NA	NA	NA
Listeria welshimeri	NZ_LT906444.1	FALSE	NA	NA	NA	NA	NA
Caldicellulosiruptor saccharolyticus DSM 8903	NC_009437.1	FALSE	NA	NA	NA	NA	NA
Clostridium ljungdahlii DSM 13528	NC_014328.1	FALSE	NA	NA	NA	NA	NA
Staphylococcus saprophyticus	NZ_LR134089.1	TRUE	70.4	86.4	375	WP_002483796.1	377
Bacillus coagulans DSM 1 = ATCC 7050	NZ_CP009709.1	TRUE	46.524	62.93333333	374	WP_029142993.1	372
Paenibacillus polymyxa	NZ_CP009909.1	TRUE	41.257	60.53333333	366	WP_019686018.1	374
Leuconostoc citreum	NZ_CP042410.1	TRUE	35.775	55.46666667	355	WP_085700007.1	411
Alkaliphilus oremlandii OhILAs	NC_009922.1	FALSE	NA	NA	NA	NA	NA
Lachnoclostridium phytofermentans ISDg	NC_010001.1	FALSE	NA	NA	NA	NA	NA
Halothermothrix orenii H 168	NC_011899.1	FALSE	NA	NA	NA	NA	NA
Clostridium novyi NT	NC_008593.1	FALSE	NA	NA	NA	NA	NA
Ruminiclostridium cellulolyticum H10	NC_011898.1	FALSE	NA	NA	NA	NA	NA
Caldanaerobacter subterraneus subsp. tengcongensis MB4	NC_003869.1	TRUE	35.897	51.46666667	351	WP_011026232.1	647
Lysinibacillus sphaericus	NZ_CP019980.1	TRUE	44.898	59.73333333	343	WP_024363247.1	364
Clostridium butyricum	NZ_CP030775.1	FALSE	NA	NA	NA	NA	NA
Natranaerobius thermophilus JW/NM-WN-LF	NC_010718.1	FALSE	NA	NA	NA	NA	NA
Macrococcus caseolyticus	NZ_CP065729.1	FALSE	NA	NA	NA	NA	NA
Ammonifex degensii KC4	NC_013385.1	FALSE	NA	NA	NA	NA	NA
Symbiobacterium thermophilum IAM 14863	NC_006177.1	FALSE	NA	NA	NA	NA	NA
Alkaliphilus metalliredigens QYMF	NC_009633.1	FALSE	NA	NA	NA	NA	NA
Geobacillus kaustophilus HTA426	NC_006510.1	FALSE	NA	NA	NA	NA	NA
Latilactobacillus sakei	NZ_CP046037.1	FALSE	NA	NA	NA	NA	NA
Listeria grayi	NZ_LR134483.1	FALSE	NA	NA	NA	NA	NA
Paenibacillus larvae subsp. larvae	NZ_CP019659.1	FALSE	NA	NA	NA	NA	NA
Acetoanaerobium sticklandii	NC_014614.1	FALSE	NA	NA	NA	NA	NA
Bacillus pseudofirmus OF4	NC_013791.2	TRUE	39.153	59.2	378	0	0

Brevibacillus brevis	NZ_LR134338.1	FALSE	NA	NA	NA	NA	NA
Butyrivibrio proteoclasticus B316	NC_014387.1	FALSE	NA	NA	NA	NA	NA
Caldicellulosiruptor bescii DSM 6725	NC_012034.1	FALSE	NA	NA	NA	NA	NA
Clostridium carboxidivorans P7	NZ_CP011803.1	FALSE	NA	NA	NA	NA	NA
Bacillus pseudomycoides DSM 12442	NZ_CM000745.1	FALSE	NA	NA	NA	NA	NA
Alicyclobacillus acidocaldarius subsp. acidocaldarius DSM 446	NC_013205.1	FALSE	NA	NA	NA	NA	NA
Thermosediminibacter oceani DSM 16646	NC_014377.1	FALSE	NA	NA	NA	NA	NA
Bacillus vallismortis	NZ_CP033052.1	TRUE	47.09	65.86666667	378	WP_121642871.1	372
Kyrpidia tusciae DSM 2912	NC_014098.1	FALSE	NA	NA	NA	NA	NA
Thermoanaerobacter brockii subsp. finnii Ako-1	NC_014964.1	FALSE	NA	NA	NA	NA	NA
Halanaerobium praevalens DSM 2228	NC_017455.1	FALSE	NA	NA	NA	NA	NA
Acetohalobium arabaticum DSM 5501	NC_014378.1	FALSE	NA	NA	NA	NA	NA
Clostridium cellulovorans 743B	NC_014393.1	TRUE	35.144	44.8	313	WP_010077562.1	332
Thermoanaerobacter italicus Ab9	NC_013921.1	FALSE	NA	NA	NA	NA	NA
Thermoanaerobacterium thermosaccharolyticum DSM 571	NC_014410.1	FALSE	NA	NA	NA	NA	NA
Thermoanaerobacter mathranii subsp. mathranii str. A3	NC_014209.1	FALSE	NA	NA	NA	NA	NA
Streptococcus intermedius	NZ_LS483436.1	TRUE	39.323	57.06666667	384	WP_003075855.1	395
Acidaminococcus fermentans DSM 20731	NC_013740.1	FALSE	NA	NA	NA	NA	NA
Lactobacillus jensenii	NZ_CP018809.1	FALSE	NA	NA	NA	NA	NA
Lactobacillus ultunensis	NZ_CP059829.1	TRUE	36.986	53.33333333	365	WP_007125708.1	374
Abiotrophia defectiva	NZ_CP053988.1	FALSE	NA	NA	NA	NA	NA
Staphylococcus caprae	NZ_AP018587.1	TRUE	80.267	90.13333333	375	WP_002441797.1	375
Weissella paramesenteroides	NZ_CP023501.1	FALSE	NA	NA	NA	NA	NA
Filifactor alocis ATCC 35896	NC_016630.1	FALSE	NA	NA	NA	NA	NA
Staphylococcus hominis	NZ_CP033732.1	TRUE	76.8	89.6	375	WP_002455544.1	375
Lentilactobacillus hilgardii	NZ_CP047121.1	TRUE	37.912	56.53333333	364	WP_003553444.1	380
Lacticaseibacillus paracasei	NC_014334.2	TRUE	37.861	51.46666667	346	WP_013246060.1	387
Peptacetobacter hiranonis	NZ_CP036523.1	FALSE	NA	NA	NA	NA	NA
Roseburia intestinalis L1-82	NZ_LR027880.1	FALSE	NA	NA	NA	NA	NA
Blautia hansenii DSM 20583	NZ_CP022413.2	FALSE	NA	NA	NA	NA	NA
Staphylococcus capitis subsp. capitis	NZ_CP007601.1	TRUE	78.4	90.93333333	375	WP_030065046.1	375

Gemella haemolysans	NZ_LR134484.1	FALSE	NA	NA	NA	NA	NA
Selenomonas sputigena ATCC 35185	NC_015437.1	FALSE	NA	NA	NA	NA	NA
Veillonella dispar	NZ_LR134375.1	FALSE	NA	NA	NA	NA	NA
Staphylococcus warneri	NZ_LR134242.1	TRUE	81.333	90.13333333	375	WP_126510455.1	375
Limosilactobacillus vaginalis	NZ_CP045240.1	TRUE	36.828	54.66666667	372	WP_081446184.1	405
Erysipelothrix rhusiopathiae	NZ_LR134439.1	FALSE	NA	NA	NA	NA	NA
Lactobacillus amylolyticus	NZ_CP031835.1	FALSE	NA	NA	NA	NA	NA
Bacillus atrophaeus subsp. globigii	NZ_CP007640.1	TRUE	47.34	65.86666667	376	WP_003325486.1	372
Syntrophothermus lipocalidus DSM 12680	NC_014220.1	FALSE	NA	NA	NA	NA	NA
Thermaerobacter marianensis DSM 12885	NC_014831.1	FALSE	NA	NA	NA	NA	NA
Syntrophobotulus glycolicus DSM 8271	NC_015172.1	FALSE	NA	NA	NA	NA	NA
Bacillus cellulosilyticus DSM 2522	NC_014829.1	FALSE	NA	NA	NA	NA	NA
Anaerococcus vaginalis	NZ_CP066014.1	FALSE	NA	NA	NA	NA	NA
Aerococcus viridans	NZ_CP014164.1	TRUE	39.842	56	379	WP_003141161.1	394
Streptococcus oralis ATCC 35037	NZ_LR134336.1	FALSE	NA	NA	NA	NA	NA
Gemella morbillorum	NZ_CP046314.1	FALSE	NA	NA	NA	NA	NA
Megamonas hypermegale	NZ_LT906446.1	FALSE	NA	NA	NA	NA	NA
Ruminococcus champanellensis 18P13 = JCM 17042	NC_021039.1	FALSE	NA	NA	NA	NA	NA
Ethanoligenens harbinense YUAN-3	NZ_CP025286.1	FALSE	NA	NA	NA	NA	NA
[Clostridium] innocuum	NZ_CP048838.1	FALSE	NA	NA	NA	NA	NA
Bacillus smithii	NZ_CP012024.1	FALSE	NA	NA	NA	NA	NA
Caldicellulosiruptor lactoaceticus 6A	NC_015949.1	FALSE	NA	NA	NA	NA	NA
Caldicellulosiruptor obsidiansis OB47	NC_014392.1	FALSE	NA	NA	NA	NA	NA
Pediococcus acidilactici	NZ_CP053421.1	FALSE	NA	NA	NA	NA	NA
Parageobacillus thermoglucosidasius	NZ_CP016622.1	FALSE	NA	NA	NA	NA	NA
Caldicellulosiruptor hydrothermalis 108	NC_014652.1	FALSE	NA	NA	NA	NA	NA
Caldicellulosiruptor owensensis OL	NC_014657.1	FALSE	NA	NA	NA	NA	NA
Leuconostoc kimchii IMSNU 11154	NC_014136.1	FALSE	NA	NA	NA	NA	NA
Cellulosilyticum lentocellum DSM 5427	NC_015275.1	FALSE	NA	NA	NA	NA	NA
Veillonella parvula	NZ_LR778174.1	FALSE	NA	NA	NA	NA	NA
Bacillus cytotoxicus	NZ_CP024109.1	FALSE	NA	NA	NA	NA	NA

[Clostridium] saccharolyticum WM1	NC_014376.1	FALSE	NA	NA	NA	NA	NA
Mahella australiensis 50-1 BON	NC_015520.1	FALSE	NA	NA	NA	NA	NA
Thermoanaerobacter wiegelii Rt8.B1	NC_015958.1	FALSE	NA	NA	NA	NA	NA
Staphylococcus lugdunensis	NZ_CP014022.1	TRUE	73.867	87.2	375	WP_002478443.1	376
Caldicellulosiruptor kristjanssonii I77R1B	NC_014721.1	FALSE	NA	NA	NA	NA	NA
Turicibacter sanguinis	NZ_CP053187.1	FALSE	NA	NA	NA	NA	NA
Halanaerobium hydrogeniformans	NC_014654.1	FALSE	NA	NA	NA	NA	NA
Streptococcus anginosus	NZ_CP012805.1	FALSE	NA	NA	NA	NA	NA
Fructilactobacillus sanfranciscensis	NZ_CP045563.1	FALSE	NA	NA	NA	NA	NA
Lysinibacillus fusiformis	NZ_CP010820.1	FALSE	NA	NA	NA	NA	NA
Streptococcus gallolyticus	NZ_CP054015.1	TRUE	39.402	56.26666667	368	WP_009853952.1	395
Roseburia hominis	NZ_LR699011.1	FALSE	NA	NA	NA	NA	NA
[Eubacterium] cellulosolvens 6	NZ_CM001487.1	FALSE	NA	NA	NA	NA	NA
Thermodesulfobium narugense DSM 14796	NC_015499.1	FALSE	NA	NA	NA	NA	NA
Desulfotomaculum ruminis DSM 2154	NC_015589.1	FALSE	NA	NA	NA	NA	NA
Thermoanaerobacterium xylanolyticum LX-11	NC_015555.1	FALSE	NA	NA	NA	NA	NA
Aerococcus urinae	NZ_CP014161.1	TRUE	35.714	52	364	WP_060778640.1	407
Streptococcus pseudoporcinus	NZ_LR134341.1	FALSE	NA	NA	NA	NA	NA
Dolosigranulum pigrum	NZ_CP040938.1	FALSE	NA	NA	NA	NA	NA
Eubacterium limosum	NZ_CP019962.1	FALSE	NA	NA	NA	NA	NA
Streptococcus parauberis NCFD 2020	NZ_AEUT02000001.1	TRUE	39.362	55.46666667	376	WP_003104137.1	382
Streptococcus porcinus	NZ_LR594050.1	TRUE	37.433	56	374	WP_093958930.1	968
Streptococcus cristatus ATCC 51100	NZ_LS483383.1	FALSE	NA	NA	NA	NA	NA
Streptococcus pseudopneumoniae IS7493	NC_015875.1	FALSE	NA	NA	NA	NA	NA
Streptococcus vestibularis	NZ_LR134275.1	FALSE	NA	NA	NA	NA	NA
Caldicellulosiruptor kronotskyensis 2002	NC_014720.1	FALSE	NA	NA	NA	NA	NA
Peptoniphilus harei	NZ_LR134524.1	FALSE	NA	NA	NA	NA	NA
Thermobacillus composti KWC4	NC_019897.1	TRUE	42.593	61.6	378	WP_015253723.1	370
Desulfallas gibsoniae DSM 7213	NC_021184.1	FALSE	NA	NA	NA	NA	NA

Lactobacillus coryniformis subsp. coryniformis KCTC 3167 = DSM 20001	NZ_CP017713.1	TRUE	36.888	53.06666667	347	WP_056943328.1	363
Leuconostoc gelidum JB7	NC_018631.1	FALSE	NA	NA	NA	NA	NA
Ligilactobacillus animalis	NZ_CP047141.1	FALSE	NA	NA	NA	NA	NA
Desulfofarcimen acetoxidans DSM 771	NC_013216.1	FALSE	NA	NA	NA	NA	NA
Leuconostoc pseudomesenteroides	NZ_CP065993.1	TRUE	35.976	51.73333333	328	WP_010291608.1	411
Desulfitobacterium dehalogenans ATCC 51507	NC_018017.1	FALSE	NA	NA	NA	NA	NA
Lactobacillus farciminis KCTC 3681 = DSM 20184	NZ_CP017702.1	FALSE	NA	NA	NA	NA	NA
Staphylococcus pseudintermedius HKU10-03	NC_014925.1	TRUE	58.981	74.93333333	373	WP_015728961.1	375
Desulfitobacterium metallireducens DSM 15288	NZ_CP007032.1	FALSE	NA	NA	NA	NA	NA
Planococcus donghaensis	NZ_CP016543.2	TRUE	42.507	59.73333333	367	WP_065527221.1	374
Fructilactobacillus fructivorans	NZ_CP045562.1	FALSE	NA	NA	NA	NA	NA
Desulfitobacterium dichloroeliminans LMG P-21439	NC_019903.1	FALSE	NA	NA	NA	NA	NA
Desulfosporosinus acidiphilus SJ4	NC_018068.1	FALSE	NA	NA	NA	NA	NA
Paenibacillus mucilaginosus K02	NC_017672.3	TRUE	45.213	63.2	376	WP_014368470.1	374
Melissococcus plutonius	NZ_AP018492.1	FALSE	NA	NA	NA	NA	NA
Lactobacillus kefiranofaciens	NZ_CP061341.1	FALSE	NA	NA	NA	NA	NA
Streptococcus pasteurianus	NZ_CP039457.1	FALSE	NA	NA	NA	NA	NA
Brevibacillus laterosporus DSM 25	NZ_CP017705.1	FALSE	NA	NA	NA	NA	NA
Paenibacillus riograndensis SBR5	NZ_LN831776.1	TRUE	41.444	59.2	374	WP_020425930.1	378
Desulfosporosinus meridiei DSM 13257	NC_018515.1	FALSE	NA	NA	NA	NA	NA
Tetragenococcus halophilus	NZ_CP012047.1	TRUE	42.815	56.8	341	WP_061841088.1	395
Enterococcus hirae	NZ_CP023011.2	TRUE	42.012	55.46666667	338	WP_010737022.1	368
Lactiplantibacillus pentosus	NZ_CP032757.1	TRUE	37.463	54.13333333	339	WP_056952577.1	369
Lactobacillus curvatus JCM 1096 = DSM 20019	NZ_CP026116.1	FALSE	NA	NA	NA	NA	NA
Mammaliicoccus sciuri	NZ_CP022046.2	TRUE	65.333	80.26666667	375	WP_078355077.1	380
Pediococcus damnosus	NZ_CP012294.1	TRUE	38.746	53.86666667	351	0	0
Streptococcus sobrinus	NZ_CP029491.1	FALSE	NA	NA	NA	NA	NA
Clostridium pasteurianum	NZ_CP013019.1	FALSE	NA	NA	NA	NA	NA

Staphylococcus xylosus	NZ_CP008724.1	TRUE	70.133	86.93333333	375	WP_042363012.1	377
Lederbergia lentus	NZ_LS483476.1	FALSE	NA	NA	NA	NA	NA
Streptococcus canis	NZ_LR134293.1	FALSE	NA	NA	NA	NA	NA
Acidaminococcus intestini	NZ_LR698962.1	FALSE	NA	NA	NA	NA	NA
Aerococcus christensenii	NZ_CP014159.1	TRUE	35.933	52.8	359	WP_060776880.1	407
Flavonifractor plautii	NZ_CP048436.1	FALSE	NA	NA	NA	NA	NA
Peribacillus simplex NBRC 15720 = DSM 1321	NZ_CP017704.1	TRUE	45.867	65.6	375	WP_063234655.1	374
Thermanaeromonas toyohensis ToBE	NZ_LT838272.1	TRUE	36.036	49.6	333	WP_084666563.1	642
Amphibacillus xylanus NBRC 15112	NC_018704.1	TRUE	38.947	59.2	380	WP_015011023.1	374
Streptococcus ratti	NZ_CP043405.1	TRUE	38.298	56.26666667	376	WP_003089044.1	399
Pediococcus claussenii ATCC BAA-344	NC_016605.1	TRUE	40.054	57.86666667	372	WP_050899587.1	390
Oscillibacter valericigenes Sjm18-20	NC_016048.1	FALSE	NA	NA	NA	NA	NA
Halobacteroides halobius DSM 5150	NC_019978.1	FALSE	NA	NA	NA	NA	NA
Halobacillus halophilus DSM 2266	NC_017668.1	FALSE	NA	NA	NA	NA	NA
Staphylococcus simiae	NZ_LT906460.1	TRUE	79.467	89.86666667	375	WP_002465334.1	375
Desulfosporosinus youngiae DSM 17734	NZ_CM001441.1	FALSE	NA	NA	NA	NA	NA
Clostridium saccharoperbutylacetonicum N1-4(HMT)	NC_020291.1	TRUE	40.526	62.4	380	WP_015394071.1	372
Acetobacterium woodii DSM 1030	NC_016894.1	FALSE	NA	NA	NA	NA	NA
Desulfosporosinus orientis DSM 765	NC_016584.1	FALSE	NA	NA	NA	NA	NA
Dehalobacter restrictus	NZ_CP046996.1	FALSE	NA	NA	NA	NA	NA
Oenococcus kitaharae DSM 17330	NZ_CM001398.1	TRUE	38.136	54.4	354	WP_007745364.1	393
Leuconostoc carnosum	NZ_CP042374.1	TRUE	35.404	50.4	322	WP_041773121.1	414
Secundilactobacillus malefermentans	NZ_CP042371.1	TRUE	37.565	55.73333333	386	WP_147657139.1	380
Lacticaseibacillus zeae	NZ_LS991421.1	TRUE	38.506	52.26666667	348	WP_070650612.1	381
Enterococcus mundtii	NZ_CP018061.1	TRUE	39.349	54.13333333	338	WP_071866577.1	368
Bacillus mojavensis	NZ_CP051464.1	TRUE	46.825	66.13333333	378	WP_168748208.1	372
Streptococcus lutetiensis	NZ_LS483403.1	FALSE	NA	NA	NA	NA	NA
Hungateiclostridium clariflavum DSM 19732	NC_016627.1	FALSE	NA	NA	NA	NA	NA
Staphylococcus hyicus	NZ_CP008747.1	TRUE	58.556	76.8	374	WP_039646290.1	379
Furfurilactobacillus rossiae	NZ_CP040586.1	TRUE	38.504	53.86666667	361	WP_178914840.1	395
Weissella confusa	NZ_CP027563.1	TRUE	37.353	50.66666667	340	WP_003608801.1	386

Gottschalkia acidurici 9a	NC_018664.1	FALSE	NA	NA	NA	NA	NA
Limosilactobacillus mucosae	NZ_CP062966.1	FALSE	NA	NA	NA	NA	NA
Paenibacillus terrae HPL-003	NC_016641.1	TRUE	42.287	63.2	376	WP_014281008.1	375
Megasphaera elsdenii 14-14	NZ_CP009240.1	FALSE	NA	NA	NA	NA	NA
Thermacetogenium phaeum DSM 12270	NC_018870.1	FALSE	NA	NA	NA	NA	NA
Enterococcus cecorum	NZ_LS483306.1	FALSE	NA	NA	NA	NA	NA
Geobacillus thermoleovorans	NZ_CP061472.1	FALSE	NA	NA	NA	NA	NA
Anaerotignum propionicum DSM 1682	NZ_CP014223.1	FALSE	NA	NA	NA	NA	NA
Thermoanaerobacter siderophilus SR4	NZ_CM001486.1	FALSE	NA	NA	NA	NA	NA
Staphylococcus equorum	NZ_CP013114.1	TRUE	71.2	86.66666667	375	WP_056935412.1	377
Lactobacillus oligofermentans DSM 15707 = LMG 22743	NZ_LN898144.1	TRUE	38.053	53.6	339	WP_057890147.1	364
Mammaliicoccus vitulinus	NZ_CP068061.1	TRUE	65.333	80.53333333	375	WP_107537127.1	380
Paeniclostridium sordellii	NZ_CP014150.1	FALSE	NA	NA	NA	NA	NA
Paenibacillus sonchi	NZ_CP068595.1	FALSE	NA	NA	NA	NA	NA
Peribacillus psychrosaccharolyticus	NZ_CP068053.1	TRUE	46.277	66.4	376	WP_040372781.1	374
Bacillus altitudinis	NZ_CP011150.1	TRUE	44.415	66.13333333	376	WP_034647339.1	375
Bacillus safensis	NZ_CP043404.1	TRUE	44.681	65.86666667	376	WP_029708077.1	375
Planococcus antarcticus DSM 14505	NZ_CP016534.2	TRUE	42.424	57.86666667	363	WP_065537298.1	374
Virgibacillus halodenitrificans	NZ_CP017962.1	FALSE	NA	NA	NA	NA	NA
Aeribacillus pallidus	NZ_CP017703.1	FALSE	NA	NA	NA	NA	NA
Erysipelothrix inopinata	NZ_CP060715.1	FALSE	NA	NA	NA	NA	NA
Streptococcus ferus	NZ_LS483343.1	FALSE	NA	NA	NA	NA	NA
Streptococcus merionis	NZ_LT906439.1	TRUE	38.855	52	332	WP_018373500.1	362
Blautia producta	NZ_CP039126.1	FALSE	NA	NA	NA	NA	NA
Clostridium tyrobutyricum	NZ_CP014170.1	FALSE	NA	NA	NA	NA	NA
Planococcus halocryophilus	NZ_CP016537.2	TRUE	42.779	59.73333333	367	WP_008497359.1	374
Paenibacillus chitinolyticus	NZ_CP026520.1	TRUE	45.503	66.93333333	378	WP_042235431.1	372
Geobacillus zalihae	NZ_CP061470.1	FALSE	NA	NA	NA	NA	NA
Parageobacillus toebii	NZ_CP070511.1	FALSE	NA	NA	NA	NA	NA
Oceanobacillus kimchii X50	NZ_CM001792.1	TRUE	44.947	63.46666667	376	WP_017798579.1	373
Staphylococcus pettenkoferi	NZ_CP022096.2	TRUE	68.533	84.53333333	375	WP_002472223.1	377
Brevibacillus agri	NZ_CP026363.1	FALSE	NA	NA	NA	NA	NA
Thermoclostridium stercorarium subsp. stercorarium DSM 8532	NC_020134.1	FALSE	NA	NA	NA	NA	NA

Lactococcus raffinolactis	NZ_CP023392.1	FALSE	NA	NA	NA	NA	NA
Tepidanaerobacter acetatoxydans Re1	NC_015519.1	FALSE	NA	NA	NA	NA	NA
Brochothrix thermosphacta	NZ_CP023643.1	FALSE	NA	NA	NA	NA	NA
Companilactobacillus crustorum	NZ_CP017996.1	TRUE	37.466	54.66666667	363	WP_075887361.1	371
Staphylococcus pasteuri SP1	NC_022737.1	TRUE	81.6	90.66666667	375	WP_023374986.1	375
Sporosarcina ureae	NZ_CP015108.1	FALSE	NA	NA	NA	NA	NA
Aerococcus urinaeequi	NZ_CP013988.1	TRUE	38.786	54.13333333	379	WP_059348484.1	394
Schleiferilactobacillus harbinensis	NZ_CP041364.1	FALSE	NA	NA	NA	NA	NA
Paenibacillus uliginis N3/975	LT840184.1	FALSE	NA	NA	NA	NA	NA
Bacillus cohnii	NZ_CP018866.1	TRUE	41.91	64.26666667	377	WP_066416116.1	376
Clostridium diolis	NZ_CP043998.1	FALSE	NA	NA	NA	NA	NA
Anaerococcus obesiensis	NZ_CP067016.1	FALSE	NA	NA	NA	NA	NA
Paenibacillus durus	NZ_CP009288.1	FALSE	NA	NA	NA	NA	NA
Paenibacillus graminis	NZ_CP009287.1	TRUE	41.176	57.86666667	374	WP_025706360.1	378
Clostridium autoethanogenum DSM 10061	NZ_CP012395.1	FALSE	NA	NA	NA	NA	NA
Clostridium saccharobutylicum DSM 13864	NC_022571.1	FALSE	NA	NA	NA	NA	NA
Clostridium chauvoei	NZ_CP027286.1	FALSE	NA	NA	NA	NA	NA
Phascolarctobacterium faecium	NZ_AP019004.1	FALSE	NA	NA	NA	NA	NA
Bacillus infantis NRRL B-14911	NC_022524.1	TRUE	42.933	64.53333333	375	WP_009793161.1	373
Bacillus toyonensis	NZ_CP064875.1	FALSE	NA	NA	NA	NA	NA
Sporosarcina pasteurii	NZ_CP038012.1	FALSE	NA	NA	NA	NA	NA
Bacillus tequilensis	NZ_CP048852.1	TRUE	49.206	66.13333333	378	WP_167872849.1	372
Thermoanaerobacterium aotearoense	NZ_CP047602.1	FALSE	NA	NA	NA	NA	NA
Bacillus velezensis	NZ_CP011937.1	TRUE	46.825	67.2	378	WP_032874152.1	372
Listeria weihenstephanensis	NZ_CP011102.1	FALSE	NA	NA	NA	NA	NA
Peptoclostridium acidaminophilum DSM 3953	NZ_CP007452.1	FALSE	NA	NA	NA	NA	NA
Paenibacillus sabinae T27	NZ_CP004078.1	FALSE	NA	NA	NA	NA	NA
Latilactobacillus fuchuensis	NZ_LT984417.1	FALSE	NA	NA	NA	NA	NA
Geobacillus thermocatenulatus	NZ_CP018058.1	FALSE	NA	NA	NA	NA	NA
Clostridium scatologenes	NZ_CP009933.1	FALSE	NA	NA	NA	NA	NA
Clostridium drakei	NZ_CP020953.1	FALSE	NA	NA	NA	NA	NA
Sporolactobacillus terrae	NZ_AP021853.1	FALSE	NA	NA	NA	NA	NA
Staphylococcus agnetis	NZ_CP045927.1	TRUE	56.417	74.93333333	374	WP_107368929.1	374
Carnobacterium divergens	NZ_CP016843.1	FALSE	NA	NA	NA	NA	NA

Geobacillus subterraneus	NZ_CP014342.1	FALSE	NA	NA	NA	NA	NA
Ruminococcus bicirculans	NZ_HF545616.1	FALSE	NA	NA	NA	NA	NA
Caldibacillus thermoamylovorans	NZ_CP023704.1	FALSE	NA	NA	NA	NA	NA
Lactiplantibacillus paraplantarum	CP028423.1	TRUE	37.026	54.66666667	343	AVW09150.1	369
Lysinibacillus varians	NZ_CP006837.1	TRUE	44.606	59.73333333	343	WP_025220216.1	364
Paenibacillus borealis	NZ_CP009285.1	TRUE	43.085	62.66666667	376	WP_042218324.1	378
Paenibacillus stellifer	NZ_CP009286.1	TRUE	45.429	58.93333333	361	WP_038699593.1	379
Paenibacillus odorifer	NZ_CP009428.1	TRUE	39.474	54.66666667	342	WP_038574344.1	367
Exiguobacterium mexicanum	NZ_CP040676.1	TRUE	41.534	60.53333333	378	WP_138858758.1	371
Thermoanaerobacter kivui	NZ_CP009170.1	TRUE	36.752	51.46666667	351	WP_084574235.1	647
Anoxybacillus gonensis	NZ_CP012152.1	FALSE	NA	NA	NA	NA	NA
Priestia aryabhattai	NZ_CP024035.1	TRUE	45.576	65.6	373	WP_033579898.1	375
Lentilactobacillus curieae	NZ_CP018906.1	TRUE	42.553	53.06666667	329	WP_035165778.1	379
Bacillus xiamenensis	NZ_CP017786.1	TRUE	45.052	66.66666667	384	WP_071169025.1	382
Clostridium baratii	NZ_CP014204.2	FALSE	NA	NA	NA	NA	NA
Clostridium argentinense	NZ_CP014176.1	FALSE	NA	NA	NA	NA	NA
Lactobacillus hokkaidonensis JCM 18461	NZ_AP014680.1	TRUE	41.994	52.8	331	WP_041092457.1	390
Companilactobacillus heilongjiangensis	NZ_CP012559.1	TRUE	41.449	56.53333333	345	WP_041499143.1	391
Clostridium aceticum	NZ_CP009687.1	FALSE	NA	NA	NA	NA	NA
Paenibacillus beijingensis	NZ_CP011058.1	FALSE	NA	NA	NA	NA	NA
Mageeibacillus indolicus UPII9-5	NC_013895.2	FALSE	NA	NA	NA	NA	NA
Lactobacillus kullabergensis	NZ_CP029477.1	FALSE	NA	NA	NA	NA	NA
Lactobacillus apis	NZ_CP029476.1	FALSE	NA	NA	NA	NA	NA
Lactobacillus helsingborgensis	NZ_CP029544.1	FALSE	NA	NA	NA	NA	NA
Salimicrobium jeotgali	NZ_CP011361.2	TRUE	41.842	62.66666667	380	WP_008588370.1	366
Clostridium bornimense	NZ_HG917868.1	FALSE	NA	NA	NA	NA	NA
Salinicoccus halodurans	NZ_CP011366.1	TRUE	63.032	81.06666667	376	WP_046789017.1	377
Staphylococcus argenteus	NZ_CP015758.1	TRUE	94.4	98.13333333	375	WP_047556898.1	375
Staphylococcus succinus	NZ_CP018199.1	TRUE	70.213	85.06666667	376	WP_073504776.1	377
Staphylococcus condimenti	NZ_CP018776.1	TRUE	70.856	85.33333333	374	WP_047131649.1	375
Bacillus circulans	NZ_CP053989.1	TRUE	43.36	62.66666667	369	WP_061799980.1	371
Lactobacillus acetotolerans	NZ_CP044496.1	FALSE	NA	NA	NA	NA	NA
Levilactobacillus koreensis	NZ_CP012033.1	TRUE	38.503	56.8	374	WP_048733235.1	101
Companilactobacillus ginsenosidimutans	NZ_CP012034.1	FALSE	NA	NA	NA	NA	NA

Jeotgalibaca dankookensis	NZ_CP019728.1	TRUE	39.42	56.26666667	345	WP_062469175.1	370	
Peribacillus butanolivorans	NZ_CP030926.1	TRUE	45.867	65.6	375	WP_098174817.1	374	
Thermoactinomyces vulgaris	NZ_CP039710.1	FALSE	NA	NA	NA	NA	NA	
Staphylococcus schweitzeri	NZ_LR134304.1	TRUE	92.8	95.73333333	375	WP_047531902.1	375	
Fructilactobacillus lindneri	NZ_CP014872.1	FALSE	NA	NA	NA	NA	NA	
Secundilactobacillus paracollinoides	NZ_CP014912.1	TRUE	41.226	57.6	359	WP_065901944.1	391	
Amylolactobacillus amylophilus DSM 20533 = JCM 1125	NZ_CP018888.1	TRUE	38.15	53.33333333	346	WP_056945804.1	372	
Lentilactobacillus kefiri	NZ_CP029971.1	TRUE	42	60.26666667	350	WP_056982831.1	393	
Moorella glycerini	NZ_CP046244.1	FALSE	NA	NA	NA	NA	NA	
Bacillus gobiensis	NZ_CP012600.1	TRUE	40.691	62.13333333	376	WP_053604838.1	371	
Liquorilactobacillus nagelii	NZ_CP018180.1	TRUE	38.873	53.33333333	355	WP_057886565.1	369	
Loigolactobacillus bifermentans	NZ_CP045872.1	TRUE	40.741	54.4	351	WP_057904439.1	371	
Lentilactobacillus parabuchneri	NZ_CP018796.1	TRUE	36.119	56.8	371	WP_057910071.1	380	
Limosilactobacillus pontis	NZ_CP045530.1	TRUE	36.765	48.53333333	340	WP_083479770.1	392	
Companilactobacillus futsaii	NZ_CP040736.1	TRUE	39.155	53.86666667	355	WP_057813131.1	396	
Lapidilactobacillus dextrinicus	NZ_CP044499.1	FALSE	NA	NA	NA	NA	NA	
Latilactobacillus graminis	NZ_CP045007.1	FALSE	NA	NA	NA	NA	NA	
Limosilactobacillus frumenti	NZ_CP044534.1	FALSE	NA	NA	NA	NA	NA	
Weissella viridescens	NZ_CP061835.1	FALSE	NA	NA	NA	NA	NA	
Pediococcus inopinatus	NZ_CP019981.1	TRUE	39.726	59.2	365	WP_057771748.1	393	
Bacillus paralicheniformis	NZ_CP023665.1	TRUE	44.058	57.86666667	345	WP_026580145.1	364	
Bacillus glycinifermentans	NZ_LT603683.1	TRUE	42.897	58.13333333	359	0	0	
Lentibacillus amyloliquefaciens	NZ_CP013862.1	TRUE	36.517	53.33333333	356	WP_068446015.1	394	
Paenibacillus naphthalenovorans	NZ_CP013652.1	TRUE	45.479	64.26666667	376	WP_062410649.1	377	
Enterococcus rotai	CP013655.1	TRUE	38.655	55.73333333	357	ALS36970.1	367	
Planococcus rifietoensis	NZ_CP013659.2	TRUE	41.962	59.46666667	367	WP_058381535.1	375	
Planococcus kocurii	NZ_CP013661.2	TRUE	41.962	58.4	367	WP_058386326.1	373	
Staphylococcus auricularis	NZ_CP065712.1	TRUE	64	80.26666667	375	WP_059106438.1	376	
Paenibacillus bovis	NZ_CP013023.1	TRUE	44.415	61.33333333	376	WP_060534837.1	382	
Bacillus halotolerans	NZ_CP029364.1	TRUE	47.09	66.4	378	WP_127696780.1	372	
Aerococcus urinaehominis	NZ_CP014163.1	TRUE	36.247	54.4	389	WP_067980627.1	408	
Limnochorda pilosa	NZ_AP014924.1	FALSE	NA	NA	NA	NA	NA	
Paenibacillus jilunlii	NZ_CP048429.1	FALSE	NA	NA	NA	NA	NA	
Faecalibaculum rodentium	NZ_CP011391.1	FALSE	NA	NA	NA	NA	NA	
Christensenella minuta	NZ_CP029256.1	FALSE	NA	NA	NA	NA	NA	

Sporosarcina psychrophila	NZ_CP014616.1	FALSE	NA	NA	NA	NA	NA
Planococcus maritimus	NZ_CP016538.2	TRUE	42.234	59.46666667	367	WP_068460859.1	375
Jeotgalibacillus malaysiensis	NZ_CP009416.1	TRUE	42.276	60.26666667	369	WP_039807049.1	375
Fictibacillus phosphorivorans	NZ_CP015378.1	TRUE	47.2	66.93333333	375	WP_082861231.1	372
Paenibacillus glucanolyticus	NZ_CP015286.1	FALSE	NA	NA	NA	NA	NA
Anoxybacillus amylolyticus	NZ_CP015438.1	FALSE	NA	NA	NA	NA	NA
Paenibacillus antarcticus	NZ_CP043611.1	TRUE	43.501	65.33333333	377	WP_068647499.1	372
Paenibacillus swuensis	NZ_CP011388.1	TRUE	45.479	64.53333333	376	WP_068611028.1	373
Streptococcus pantholopis	NZ_CP014699.1	TRUE	36.658	55.46666667	371	WP_067063517.1	972
Enterococcus thailandicus	NZ_CP023074.1	FALSE	NA	NA	NA	NA	NA
Clostridium septicum	NZ_CP023671.1	FALSE	NA	NA	NA	NA	NA
Planococcus plakortidis	NZ_CP016539.2	TRUE	44.669	58.4	347	WP_068872425.1	375
Streptococcus halotolerans	NZ_CP014835.1	TRUE	37.5	55.46666667	368	WP_099091454.1	969
Streptococcus marmotae	NZ_CP015196.1	FALSE	NA	NA	NA	NA	NA
Bacillus wiedmannii	NZ_CP032365.1	FALSE	NA	NA	NA	NA	NA
Paenibacillus kribbensis	NZ_CP020028.1	TRUE	41.27	61.6	378	WP_094156485.1	374
Paenibacillus yonginensis	NZ_CP014167.1	FALSE	NA	NA	NA	NA	NA
Bacillus beveridgei	NZ_CP012502.1	TRUE	40.608	61.6	362	WP_157101023.1	373
Clostridium taeniosporum	NZ_CP017253.2	FALSE	NA	NA	NA	NA	NA
Geosporobacter ferrireducens	NZ_CP017269.1	FALSE	NA	NA	NA	NA	NA
Weissella soli	NZ_CP017326.1	FALSE	NA	NA	NA	NA	NA
Streptococcus himalayensis	NZ_CP016953.1	TRUE	36.885	57.6	366	WP_068992780.1	367
Clostridium formicaceticum	NZ_CP020559.1	FALSE	NA	NA	NA	NA	NA
Clostridium cochlearium	NZ_LT906477.1	FALSE	NA	NA	NA	NA	NA
Clostridium gasigenes	NZ_CP071376.1	TRUE	40.426	59.46666667	376	WP_167867430.1	374
Planococcus faecalis	NZ_CP019401.1	TRUE	42.234	58.4	367	WP_071153996.1	373
Vagococcus teuberi	NZ_CP017267.1	TRUE	39.701	52.26666667	335	WP_157093687.1	368
Eubacterium callanderi	NC_014624.2	FALSE	NA	NA	NA	NA	NA
Clostridium estertheticum subsp. estertheticum	NZ_CP015756.1	TRUE	38.624	60.26666667	378	WP_071612659.1	372
Jeotgalicoccus saudimassiliensis	NZ_CCSE01000001.1	TRUE	37.941	52	340	WP_035811743.1	368
Ndongobacter massiliensis	NZ_LT635480.1	FALSE	NA	NA	NA	NA	NA
Weissella jogaejeotgali	NZ_CP014332.1	FALSE	NA	NA	NA	NA	NA
Ezakiella massiliensis	NZ_LT635475.1	FALSE	NA	NA	NA	NA	NA
Lachnoclostridium phocaeense	NZ_LT635479.1	FALSE	NA	NA	NA	NA	NA
Kroppenstedtia eburnea	NZ_CP048103.1	FALSE	NA	NA	NA	NA	NA

Bacillus weihaiensis	NZ_CP016020.1	TRUE	42.216	64.8	379	WP_072581643.1	380
Erysipelothrix larvae	NZ_CP013213.1	FALSE	NA	NA	NA	NA	NA
Desulfotomaculum ferrireducens	NZ_CP019698.1	FALSE	NA	NA	NA	NA	NA
Novibacillus thermophilus	NZ_CP019699.1	FALSE	NA	NA	NA	NA	NA
Virgibacillus dokdonensis	NZ_CP018622.1	FALSE	NA	NA	NA	NA	NA
Anaerococcus mediterraneensis	NZ_LT635772.1	FALSE	NA	NA	NA	NA	NA
Mesobacillus jeotgali	NZ_CP025025.1	TRUE	43.386	64	378	WP_102261517.1	374
Halobacillus mangrovi	NZ_CP020772.1	FALSE	NA	NA	NA	NA	NA
Staphylococcus lutrae	NZ_CP020773.1	TRUE	59.2	73.86666667	375	WP_085238010.1	375
Macrococcus canis	NZ_CP047361.1	FALSE	NA	NA	NA	NA	NA
Paenibacillus thiaminolyticus	NZ_CP041405.1	FALSE	NA	NA	NA	NA	NA
Herbinix luporum	NZ_LN879430.1	FALSE	NA	NA	NA	NA	NA
Paenibacillus donghaensis	NZ_CP021780.1	TRUE	42.553	59.46666667	376	WP_087918637.1	373
Acutalibacter muris	NZ_CP021422.1	FALSE	NA	NA	NA	NA	NA
Virgibacillus necropolis	NZ_CP022437.1	FALSE	NA	NA	NA	NA	NA
Dehalobacterium formicoaceticum	NZ_CP022121.1	TRUE	38.421	58.13333333	380	WP_089610861.1	376
Paenibacillus physcomitrellae	NZ_CP022584.1	FALSE	NA	NA	NA	NA	NA
Mogibacterium pumilum	NZ_CP016199.1	FALSE	NA	NA	NA	NA	NA
Tumebacillus algifaecis	NZ_CP022657.1	FALSE	NA	NA	NA	NA	NA
Paenibacillus ihbetae	NZ_CP016809.1	FALSE	NA	NA	NA	NA	NA
Staphylococcus muscae	NZ_LT906464.1	TRUE	58.4	75.46666667	375	WP_095116134.1	375
Mammaliicoccus stepanovicii	NZ_LT906462.1	TRUE	65.6	82.66666667	375	WP_095089585.1	379
Veillonella rodentium	NZ_LT906470.1	FALSE	NA	NA	NA	NA	NA
Cytobacillus kochii	NZ_CP022983.1	TRUE	46.561	65.86666667	378	WP_095372668.1	371
Staphylococcus saccharolyticus	NZ_CP066042.1	TRUE	77.067	89.33333333	375	WP_115313708.1	375
Clostridium isatidis	NZ_CP016786.1	FALSE	NA	NA	NA	NA	NA
Streptococcus troglodytae	NZ_AP014612.1	FALSE	NA	NA	NA	NA	NA
Aneurinibacillus soli	NZ_AP017312.1	FALSE	NA	NA	NA	NA	NA
Murdochiella vaginalis	NZ_LT632322.1	FALSE	NA	NA	NA	NA	NA
Companilactobacillus allii	NZ_CP019323.1	TRUE	38.859	57.86666667	368	WP_076618342.1	368
Enterococcus wangshanyuanii	NZ_CP021874.1	TRUE	39.42	52.53333333	345	WP_088271005.1	367
Acetivibrio saccincola	NZ_CP025197.1	FALSE	NA	NA	NA	NA	NA
Monoglobus pectinilyticus	NZ_CP020991.1	FALSE	NA	NA	NA	NA	NA
Terribacillus goriensis	NZ_CP008876.1	TRUE	55.956	72.8	361	WP_038557762.1	369
Pseudoclostridium thermosuccinogenes	NZ_CP021850.1	FALSE	NA	NA	NA	NA	NA

Staphylococcus felis	NZ_CP027770.1	TRUE	59.947	77.06666667	377	WP_103209055.1	380
Virgibacillus phasianinus	NZ_CP022315.1	FALSE	NA	NA	NA	NA	NA
Streptococcus pluranimalium	NZ_CP025536.1	FALSE	NA	NA	NA	NA	NA
Mogibacterium diversum	NZ_CP027228.1	FALSE	NA	NA	NA	NA	NA
Fastidiosipila sanguinis	NZ_CP027226.1	FALSE	NA	NA	NA	NA	NA
Tumebacillus avium	NZ_CP021434.1	TRUE	44.149	62.4	376	WP_087459141.1	377
Anaerobacillus isosaccharinicus	NZ_CP063356.1	FALSE	NA	NA	NA	NA	NA
Thermodesulfobium acidiphilum	NZ_CP020921.1	FALSE	NA	NA	NA	NA	NA
Planococcus versutus	NZ_CP016540.2	TRUE	42.234	59.46666667	367	WP_049694426.1	373
Romboutsia ilealis	NZ_LN555523.1	FALSE	NA	NA	NA	NA	NA
Paenibacillus barcinonensis	NZ_CP054614.1	TRUE	44.681	64.53333333	376	WP_110895968.1	376
Leuconostoc suionicum	NZ_CP015247.1	TRUE	39.295	56.8	369	WP_072614397.1	396
Lachnoanaerobaculum umeaense	NZ_CP032364.1	FALSE	NA	NA	NA	NA	NA
Macrococcus bohemicus	NZ_CP054482.1	FALSE	NA	NA	NA	NA	NA
Lactobacillus terrae	NZ_CP024610.1	FALSE	NA	NA	NA	NA	NA
Bacillus luti	NZ_CP040336.1	TRUE	38.904	57.33333333	365	WP_156576495.1	377
Bacillus tropicus	NZ_CP049019.1	FALSE	NA	NA	NA	NA	NA
Bombilactobacillus bombi	NZ_CP031513.1	FALSE	NA	NA	NA	NA	NA
Suicoccus acidiformans	NZ_CP023434.1	TRUE	36.467	53.06666667	351	WP_118990977.1	402
Megasphaera stantonii	NZ_CP029462.1	TRUE	39.385	55.73333333	358	WP_107195535.1	367
Lactobacillus paragasseri	NZ_AP018549.1	FALSE	NA	NA	NA	NA	NA
Kyrpidia spormannii	NZ_CP024955.1	FALSE	NA	NA	NA	NA	NA
Streptococcus respiraculi	NZ_CP022680.1	TRUE	35.925	54.4	373	WP_119876388.1	968
Streptococcus ruminantium	NZ_AP018400.1	FALSE	NA	NA	NA	NA	NA
Eubacterium maltosivorans	NZ_CP029487.1	FALSE	NA	NA	NA	NA	NA
Biomaibacter acetigenes	NZ_CP033169.1	FALSE	NA	NA	NA	NA	NA
Tetragenococcus osmophilus	NZ_CP027783.1	TRUE	41.935	56.26666667	341	WP_123933954.1	395
Abyssicoccus albus	NZ_CP019573.1	FALSE	NA	NA	NA	NA	NA
Staphylospora marina	NZ_CP034118.1	FALSE	NA	NA	NA	NA	NA
Petrocella atlantisensis	NZ_LR130778.1	FALSE	NA	NA	NA	NA	NA
Intestinibaculum porci	NZ_AP019309.1	FALSE	NA	NA	NA	NA	NA
Paenibacillus lentus	NZ_CP034248.1	FALSE	NA	NA	NA	NA	NA
Paenibacillus baekrokdamisoli	NZ_AP019308.1	TRUE	43.351	63.73333333	376	WP_125655979.1	134
Peptoniphilus ivorii	NZ_LR134523.1	FALSE	NA	NA	NA	NA	NA
Methylomusa anaerophila	NZ_AP018449.1	TRUE	35.227	52	352	WP_126306787.1	645
Vagococcus carniphilus	NZ_CP060720.1	TRUE	37.366	59.2	372	WP_126790925.1	395

Anoxybacter fermentans	NZ_CP016379.1	FALSE	NA	NA	NA	NA	NA
Caldicellulosiruptor changbaiensis	NZ_CP034791.1	FALSE	NA	NA	NA	NA	NA
Oenococcus sicerae	NZ_CP029684.2	TRUE	39.496	55.46666667	357	WP_128686440.1	372
Ureibacillus thermophilus	CP036528.1	TRUE	45.623	65.33333333	377	QBK25510.1	376
Keratinibaculum paraultunense	NZ_CP068564.1	FALSE	NA	NA	NA	NA	NA
Paenisporosarcina antarctica	NZ_CP038015.1	TRUE	44.533	66.93333333	375	WP_134210861.1	374
Dysosmobacter welbionis	NZ_CP034413.2	FALSE	NA	NA	NA	NA	NA
Levilactobacillus suantsaii	NZ_CP059603.1	TRUE	39.944	55.73333333	358	WP_182150922.1	393
Anaerostipes rhamnosivorans	NZ_CP040058.1	FALSE	NA	NA	NA	NA	NA
Hathewaya histolytica	NZ_LR590481.1	FALSE	NA	NA	NA	NA	NA
Lysinibacillus timonensis	NZ_LT985980.1	TRUE	45.358	67.46666667	377	WP_106785157.1	374
Bacillus ciccensis	NZ_CP041305.1	FALSE	NA	NA	NA	NA	NA
Sporomusa termitida	NZ_CP036259.1	FALSE	NA	NA	NA	NA	NA
Paenibacillus guangzhouensis	NZ_CP045293.1	FALSE	NA	NA	NA	NA	NA
Paenibacillus cellulositrophicus	NZ_CP045295.1	TRUE	44.681	64	376	WP_152401067.1	378
Paenibacillus brasilensis	NZ_CP045298.1	TRUE	40.476	61.33333333	378	WP_152380333.1	374
Thermoanaerosceptrum fracticalcis	NZ_CP045798.1	FALSE	NA	NA	NA	NA	NA
Heliorestis convoluta	NZ_CP045875.1	FALSE	NA	NA	NA	NA	NA
Cohnella candidum	NZ_CP033433.1	TRUE	43.57	64	381	WP_123042705.1	378
Faecalibacillus intestinalis	NZ_AP024085.1	FALSE	NA	NA	NA	NA	NA
Cohnella abietis	NZ_AP019400.1	TRUE	42.042	56.53333333	333	WP_130609345.1	398
Salicibibacter halophilus	NZ_CP035485.1	TRUE	69.333	86.4	375	WP_142088024.1	377
Radiobacillus deserti	NZ_CP041666.1	FALSE	NA	NA	NA	NA	NA
Paenibacillus psychroresistens	NZ_CP034235.1	TRUE	39.05	60	379	WP_155698896.1	373
Enterococcus lactis	NZ_CP065211.1	TRUE	41.52	56	342	WP_002292552.1	363
Salicibibacter kimchii	NZ_CP031092.1	FALSE	NA	NA	NA	NA	NA
Gudongella oleilytica	NZ_CP035130.1	FALSE	NA	NA	NA	NA	NA
Acetilactobacillus jinshanensis	NZ_CP034726.1	FALSE	NA	NA	NA	NA	NA
Saccharibacillus brassicae	NZ_CP041217.1	TRUE	42.781	62.13333333	374	WP_141448256.1	377
Psychrobacillus glaciei	NZ_CP031223.1	TRUE	43.869	61.86666667	367	WP_151700783.1	374
Sporosarcina ureilytica	NZ_CP017560.1	FALSE	NA	NA	NA	NA	NA
Streptococcus koreensis	NZ_CP032620.1	FALSE	NA	NA	NA	NA	NA
Streptococcus gwangjuense	NZ_CP032621.1	FALSE	NA	NA	NA	NA	NA
Streptococcus periodonticum	NZ_CP034543.1	FALSE	NA	NA	NA	NA	NA
Alkalihalobacillus miscanthi	NZ_CP042163.1	FALSE	NA	NA	NA	NA	NA
Paenibacillus lutimineralis	NZ_CP034346.1	TRUE	41.543	54.13333333	337	WP_126998461.1	397

Dialister massiliensis	NZ_LT996885.1	FALSE	NA	NA	NA	NA	NA
Aminipila butyrica	NZ_CP048649.1	FALSE	NA	NA	NA	NA	NA
Paenibacillus albus	NZ_CP034437.1	FALSE	NA	NA	NA	NA	NA
Massilistercora timonensis	NZ_LT990039.1	FALSE	NA	NA	NA	NA	NA
Jeotgalibaca arthritidis	NZ_CP049740.1	TRUE	40.113	57.33333333	354	WP_166162819.1	368
Paenibacillus protaetiae	NZ_CP035492.1	FALSE	NA	NA	NA	NA	NA
Jeotgalibaca porci	NZ_CP049889.1	TRUE	37.433	55.73333333	374	WP_166063722.1	363
Enterococcus saigonensis	NZ_AP022822.1	FALSE	NA	NA	NA	NA	NA
Kroppenstedtia pulmonis	NZ_CP048104.1	FALSE	NA	NA	NA	NA	NA
Jeotgalibaca ciconiae	NZ_CP034465.1	TRUE	37.808	58.93333333	365	WP_126110928.1	367
Weissella cryptocerci	NZ_CP037940.1	TRUE	36.188	53.06666667	362	WP_133362852.1	389
Amedibacterium intestinale	NZ_AP019711.1	FALSE	NA	NA	NA	NA	NA
Anoxybacillus caldiproteolyticus	NZ_CP064060.1	FALSE	NA	NA	NA	NA	NA
Bacillus dafuensis	NZ_CP042593.1	FALSE	NA	NA	NA	NA	NA
Planococcus dechangensis	NZ_CP059540.1	TRUE	42.234	59.46666667	367	WP_182092828.1	375
Erysipelothrix piscisicarius	NZ_CP034234.1	FALSE	NA	NA	NA	NA	NA
Anaerocolumna cellulosilytica	NZ_AP023367.1	FALSE	NA	NA	NA	NA	NA
Paenibacillus lycopersici	NZ_CP048209.1	FALSE	NA	NA	NA	NA	NA
Paenibacillus rhizovicinus	NZ_CP048286.1	TRUE	44.149	62.4	376	WP_162637954.1	374
Clostridium thermarum	NZ_CP040924.1	FALSE	NA	NA	NA	NA	NA
Lactococcus taiwanensis	NZ_CP070872.1	TRUE	41.159	52	328	WP_205871913.1	360
Blautia argi	NZ_CP030280.1	FALSE	NA	NA	NA	NA	NA
Clostridium fermenticellae	NZ_CP032416.1	FALSE	NA	NA	NA	NA	NA
Veillonella nakazawae	NZ_AP022321.1	FALSE	NA	NA	NA	NA	NA
Paraliobacillus zengyii	NZ_CP029797.1	TRUE	44	64.8	375	WP_112179808.1	375
Streptococcus chenjunshii	NZ_CP031733.1	TRUE	40.426	57.33333333	376	WP_116877593.1	399
Vagococcus zengguangii	NZ_CP039712.1	FALSE	NA	NA	NA	NA	NA
Bacillus subtilis subsp. spizizenii ATCC 6633	NZ_CP034943.1	TRUE	47.884	66.93333333	378	WP_003226431.1	372
Bacillus inaquosorum	NZ_CP013984.1	TRUE	47.09	65.06666667	378	WP_060398995.1	372
Apilactobacillus bombintestini	NZ_CP032626.1	FALSE	NA	NA	NA	NA	NA
Lactococcus allomyrinae	NZ_CP032627.1	TRUE	40.588	53.6	340	WP_120772358.1	361
Lactococcus carnosus	NZ_CP017194.1	FALSE	NA	NA	NA	NA	NA
Lactococcus paracarnosus	NZ_CP017195.1	FALSE	NA	NA	NA	NA	NA
Staphylococcus debuckii	NZ_CP033460.1	TRUE	70.133	84.53333333	375	WP_123144280.1	374
Vagococcus coleopterorum	NZ_CP049886.1	FALSE	NA	NA	NA	NA	NA

Vagococcus hydrophili	NZ_CP049887.1	TRUE	44.838	56.8	339	WP_166034452.1	395
Bacillus vini	NZ_CP065425.1	FALSE	NA	NA	NA	NA	NA
Companilactobacillus pabuli	NZ_CP049366.1	FALSE	NA	NA	NA	NA	NA
Ruminiclostridium herbifermentans	NZ_CP061336.1	FALSE	NA	NA	NA	NA	NA
Lactococcus lactis subsp. cremoris KW2	NC_022369.1	TRUE	38.522	55.2	379	WP_021037634.1	391
Koleobacter methoxysyntrophicus	NZ_CP059066.1	FALSE	NA	NA	NA	NA	NA
Oceanobacillus zhaokaii	NZ_CP024848.1	TRUE	48.529	62.66666667	340	WP_114916522.1	368
Staphylococcus lloydii	NZ_CP064056.1	TRUE	69.681	86.13333333	376	WP_195718417.1	377
Anaerocolumna sedimenticola	NZ_CP048000.1	FALSE	NA	NA	NA	NA	NA

Table S5. Genes deregulated in EI046/JE2 strain with P-value < 0.05.

Locus	Gene symbol	Description	Feature	Regulator	Log2FoldChange	P-value
SAUSA300_0107	*nptA*	Na/Pi cotransporter family protein	CDS	GraRS	0.529171	5.57E-05
SAUSA300_0170	*aldA*	aldehyde dehydrogenase	CDS	CcpA	-0.502329	6.55E-03
SAUSA300_0181	*ausA*	non-ribosomal peptide synthetase	CDS		-0.233138	3.11E-02
SAUSA300_0185	*argJ*	bifunctional ornithine acetyltransferase/N-acetylglutamate synthase protein	CDS	ArgR	-0.406380	2.40E-02
SAUSA300_0188	*brnQ1*	branched-chain amino acid transport system II carrier protein	CDS	GraRS	0.240291	2.18E-02
SAUSA300_0189	-	isochorismatase	CDS		0.691455	2.88E-05
SAUSA300_0190	-	indole-3-pyruvate decarboxylase	CDS	CcpA	0.608584	3.05E-04
SAUSA300_0212	-	Gfo/Idh/MocA family oxidoreductase	CDS	MalR	-0.453878	1.64E-03
SAUSA300_0213	-	Gfo/Idh/MocA family oxidoreductase	CDS	MalR	-0.294745	2.92E-02
SAUSA300_0214	-	hypothetical protein	CDS	MalR	-0.342553	1.93E-02
SAUSA300_0221	*pflA*	pyruvate formate-lyase activating enzyme	CDS	Rex	-0.607220	4.10E-03
SAUSA300_0278	*esxA*	hypothetical protein	CDS	GraRS	-0.338047	6.93E-03
SAUSA300_0283	*essC*	essC protein	CDS		-0.358306	2.51E-02
SAUSA300_0339	-	hypothetical protein	CDS	QsrR	-0.481196	1.90E-03
SAUSA300_0340	-	NADH-dependent FMN reductase	CDS	QsrR	-0.552657	3.82E-03
SAUSA300_0358	*metF*	bifunctional homocysteine S-methyltransferase/5,10-methylenetetrahydrofolate reductase protein	CDS	MgrA	-0.395075	3.40E-02
SAUSA300_0372	-	hypothetical protein	CDS	SigB	-0.713585	2.71E-03
SAUSA300_0386	*xpt*	xanthine phosphoribosyltransferase	CDS	G-box	0.355888	1.05E-03
SAUSA300_0387	*pbuX*	xanthine permease	CDS	G-box	0.271456	2.97E-03
SAUSA300_0424	*zagA*	putative cobalamin synthesis protein	CDS	Zur	0.457842	4.97E-02
SAUSA300_0429	-	hypothetical protein	CDS		-0.228977	3.55E-02
SAUSA300_0431	-	hypothetical protein	CDS		-0.558496	4.45E-02
SAUSA300_0445	*gltB*	glutamate synthase, large subunit	CDS	GltC	-0.446180	8.17E-03
SAUSA300_0446	*gltD*	glutamate synthase subunit beta	CDS	GltC	-0.390831	9.85E-03
SAUSA300_0455	*rrsA*	16S ribosomal RNA	rRNA	rRNA	-3.081901	3.16E-06
SAUSA300_0456	*rrl1*	23S ribosomal RNA	rRNA	rRNA	-2.349657	2.19E-05
SAUSA300_0469	*rnmV*	hypothetical protein	CDS		-0.324606	1.09E-02
SAUSA300_0470	*ksgA*	dimethyladenosine transferase	CDS		-0.279723	3.02E-02
SAUSA300_0476	-	hypothetical protein	CDS		0.358910	2.06E-02
SAUSA300_0499	*rrsB*	16S ribosomal RNA	rRNA	rRNA	-3.068966	3.55E-06
SAUSA300_0501	*rrlB*	23S ribosomal RNA	rRNA	rRNA	-2.329682	2.08E-05

SAUSA300_0566	-	amino acid permease	CDS	SigB	-0.460741	5.52E-03
SAUSA300_0570	*pta*	phosphotransacetylase	CDS		0.293665	7.75E-03
SAUSA300_0571	*lipL*	lipoate-protein ligase A family protein	CDS		0.213388	3.22E-02
SAUSA300_0591	-	acetyltransferase	CDS		0.310231	2.06E-02
SAUSA300_0595	-	hypothetical protein	CDS		0.327664	7.42E-03
SAUSA300_0629	*pbp4*	penicillin-binding protein 4	CDS		0.260920	4.31E-02
SAUSA300_0632	-	hypothetical protein	CDS	CodY	0.202688	4.72E-02
SAUSA300_0637	*dhaL*	dihydroxyacetone kinase subunit DhaL	CDS	CcpA	-0.438330	1.70E-02
SAUSA300_0693	*saeP*	hypothetical protein	CDS	SaeRS	0.327637	7.58E-03
SAUSA300_0712	*dtpT*	amino acid/peptide transporter (peptide:H+ symporter)	CDS	CodY	0.288877	4.16E-03
SAUSA300_0718	*sstA*	iron compound ABC transporter permease	CDS	Fur	0.424746	2.93E-03
SAUSA300_0719	*sstB*	iron compound ABC transporter permease	CDS	Fur	0.381946	2.84E-02
SAUSA300_0774	*emp*	secretory extracellular matrix and plasma binding protein	CDS	MgrA	-0.384724	4.14E-02
SAUSA300_0815	*ear*	Ear protein	CDS	SaeRS	-0.902575	6.36E-05
SAUSA300_0841	-	hypothetical protein	CDS		0.351338	3.66E-03
SAUSA300_0856	*kapB*	hypothetical protein	CDS		0.362823	2.41E-02
SAUSA300_0857	*ppiB*	hypothetical protein	CDS		0.307918	1.60E-03
SAUSA300_0858	-	hypothetical protein	CDS		-0.893341	2.33E-05
SAUSA300_0859	-	NADH-dependent flavin oxidoreductase	CDS		-8.080708	2.15E-64
SAUSA300_0860	*rocD*	ornithine--oxo-acid transaminase	CDS	CcpA	2.820476	1.35E-76
SAUSA300_0861	*gudB*	NAD-specific glutamate dehydrogenase	CDS	CcpA	1.924808	2.87E-35
SAUSA300_0877	*clpB*	chaperone clpB	CDS	CtsR	-0.312933	3.11E-02
SAUSA300_0897	*trpS*	tryptophanyl-tRNA synthetase	CDS		0.215267	4.97E-02
SAUSA300_0964	-	hypothetical protein	CDS		0.350311	2.00E-03
SAUSA300_0971	*purL*	phosphoribosylformylglycinamidine synthase II	CDS	PurR	-0.325970	3.10E-02
SAUSA300_0972	*purF*	amidophosphoribosyltransferase	CDS	PurR	-0.329243	2.65E-02
SAUSA300_0973	*purM*	phosphoribosylaminoimidazole synthetase	CDS	PurR	-0.353701	1.31E-02
SAUSA300_0974	*purN*	phosphoribosylglycinamide formyltransferase	CDS	PurR	-0.378502	7.98E-03
SAUSA300_0975	*purH*	bifunctional phosphoribosylaminoimidazolecarboxamide formyltransferase/IMP cyclohydrolase	CDS	PurR	-0.386208	4.08E-03
SAUSA300_0976	*purD*	phosphoribosylamine--glycine ligase	CDS	PurR	-0.349812	1.59E-02
SAUSA300_1005	*mntH*	manganese transport protein MntH	CDS	MntR	-0.415872	3.06E-02
SAUSA300_1008	-	hypothetical protein	CDS		0.482818	1.25E-02
SAUSA300_1010	-	hypothetical protein	CDS		0.320503	4.15E-02
SAUSA300_1030	*isdC*	iron transport associated domain-containing protein	CDS	Fur	0.205684	4.27E-02
SAUSA300_1048	*sdhB*	succinate dehydrogenase iron-sulfur subunit	CDS	CcpA	-0.439766	8.33E-03

SAUSA300_1053	*flr*	formyl peptide receptor-like 1 inhibitory protein	CDS	MgrA	-0.597587	2.22E-02
SAUSA300_1058	*hla*	alpha-hemolysin	CDS	SaeRS	-0.369417	7.84E-03
SAUSA300_1059	*ssl12*	superantigen-like protein	CDS		-0.698406	1.45E-02
SAUSA300_1090	-	hypothetical protein	CDS		0.268964	3.77E-02
SAUSA300_1091	*pyrR*	bifunctional pyrimidine regulatory protein PyrR uracil phosphoribosyltransferase	CDS	TraP	0.977715	5.34E-04
SAUSA300_1092	*pyrP*	uracil permease	CDS	PyrR	1.325441	2.22E-03
SAUSA300_1093	*pyrB*	aspartate carbamoyltransferase catalytic subunit	CDS	PyrR	1.160946	6.71E-06
SAUSA300_1094	*pyrC*	dihydroorotase	CDS	PyrR	1.113761	3.38E-05
SAUSA300_1095	*carA*	carbamoyl phosphate synthase small subunit	CDS	PyrR	1.093776	1.33E-05
SAUSA300_1096	*carB*	carbamoyl phosphate synthase large subunit	CDS	PyrR	1.057255	5.10E-06
SAUSA300_1097	*pyrF*	orotidine 5'-phosphate decarboxylase	CDS	PyrR	1.057736	1.15E-05
SAUSA300_1098	*pyrE*	orotate phosphoribosyltransferase	CDS	PyrR	1.051715	3.79E-06
SAUSA300_1099	-	hypothetical protein	CDS	PyrR	0.914792	2.71E-04
SAUSA300_1100	-	hypothetical protein	CDS		0.333136	6.28E-03
SAUSA300_1117	*rpmB*	50S ribosomal protein L28	CDS		0.343053	1.29E-02
SAUSA300_1131	*rpsP*	30S ribosomal protein S16	CDS		0.284702	9.43E-03
SAUSA300_1198	*hflX*	putative GTP-binding protein	CDS	MgrA	0.396897	4.02E-03
SAUSA300_1200	*glnR*	glutamine synthetase repressor	CDS	GlnR	0.771931	2.66E-03
SAUSA300_1201	*glnA*	glutamine synthetase, type I	CDS	GlnR	0.767979	5.03E-05
SAUSA300_1222	*nuc2*	thermonuclease	CDS		0.377881	3.41E-02
SAUSA300_1231	-	gamma-aminobutyrate permease	CDS	CodY	0.243291	4.28E-02
SAUSA300_1233	*rpmG2*	50S ribosomal protein L33	CDS	Zur	0.458785	1.30E-03
SAUSA300_1241	-	hypothetical protein	CDS		0.294555	1.73E-02
SAUSA300_1244	*mscL*	large-conductance mechanosensitive channel	CDS		0.322854	1.35E-02
SAUSA300_1245	*opuD1*	glycine betaine transporter opuD	CDS		0.303888	2.15E-03
SAUSA300_1305	*sucB*	dihydrolipoamide succinyltransferase	CDS	AgrA	-0.268837	3.28E-02
SAUSA300_1309	-	IS200 family transposase	CDS		-0.268157	4.80E-02
SAUSA300_1322	*cvfC*	hypothetical protein	CDS	BSH	0.187451	3.91E-02
SAUSA300_1323	-	hypothetical protein	CDS	BSH	0.340727	3.57E-03
SAUSA300_1329	-	amino acid permease	CDS	CodY	-0.457359	9.36E-03
SAUSA300_1330	*ilvA1*	threonine dehydratase	CDS	CodY	-0.542518	4.67E-04
SAUSA300_1331	*ald1*	alanine dehydrogenase	CDS	CodY	-0.459418	9.73E-03
SAUSA300_1334	-	hypothetical protein	CDS		0.482534	3.04E-02
SAUSA300_1340	*recU*	Holliday junction-specific endonuclease	CDS		0.276869	2.09E-02
SAUSA300_1341	*pbp2*	penicillin binding protein 2	CDS		0.247509	2.43E-02

SAUSA300_1367	*cmk*	cytidylate kinase	CDS		0.247354	3.38E-02
SAUSA300_1368	*ansA*	L-asparaginase	CDS		0.420570	6.91E-04
SAUSA300_1369	*ypdA*	pyridine nucleotide-disulfide oxidoreductase	CDS	BSH	0.240928	3.93E-02
SAUSA300_1456	*malA*	alpha-D-1,4-glucosidase	CDS	CcpA	-0.426726	1.73E-02
SAUSA300_1495	-	hypothetical protein	CDS	CodY	0.283881	3.87E-03
SAUSA300_1525	*glyS*	glycyl-tRNA synthetase	CDS	T-box	0.187122	3.86E-02
SAUSA300_1545	*rpsT*	30S ribosomal protein S20	CDS		0.239290	3.63E-02
SAUSA300_1558	*mtnN*	5'-methylthioadenosine/S-adenosylhomocysteine nucleosidase	CDS		0.250736	3.00E-02
SAUSA300_1581	-	hypothetical protein	CDS	SigB	-0.516195	1.38E-02
SAUSA300_1582	*csbD*	hypothetical protein	CDS	SigB	-0.489517	5.54E-03
SAUSA300_1583	*cymR*	hypothetical protein	CDS		0.309793	1.98E-03
SAUSA300_1607	-	hypothetical protein	CDS		0.291708	2.37E-02
SAUSA300_1653	-	metal-dependent hydrolase	CDS	SigB	0.292049	4.35E-02
SAUSA300_1666	*rpsD*	30S ribosomal protein S4	CDS		0.418813	1.93E-02
SAUSA300_1678	*fhs*	formate--tetrahydrofolate ligase	CDS	GraRS	-0.384792	1.61E-02
SAUSA300_1680	*acuA*	acetoin utilization protein AcuA	CDS	CcpA	-0.767489	3.32E-02
SAUSA300_1711	*putA*	proline dehydrogenase	CDS	CcpA	-0.692700	2.60E-02
SAUSA300_1731	*pckA*	phosphoenolpyruvate carboxykinase	CDS	CcpA	-0.369661	3.92E-02
SAUSA300_1760	*epiG*	lantibiotic epidermin immunity protein F	CDS	SigB	-0.463894	2.46E-02
SAUSA300_1797	-	hypothetical protein	CDS		0.362438	7.77E-03
SAUSA300_1802	-	hypothetical protein	CDS		0.420807	7.16E-03
SAUSA300_1838	*rrl3*	23S ribosomal RNA	rRNA	rRNA	-2.348891	1.79E-05
SAUSA300_1841	*rrsD*	16S ribosomal RNA	rRNA	rRNA	-3.075666	3.50E-06
SAUSA300_1848	-	hypothetical protein	CDS		0.241942	1.07E-02
SAUSA300_1857	-	hypothetical protein	CDS		0.467007	8.24E-03
SAUSA300_1864	-	hypothetical protein	CDS	SigB	-0.333991	4.47E-02
SAUSA300_1873	*murT*	Mur ligase family protein	CDS		0.211002	4.01E-02
SAUSA300_1883	*putP*	high affinity proline permease	CDS	CodY	0.308514	2.48E-02
SAUSA300_1940	-	phage portal protein	CDS		0.458627	3.24E-02
SAUSA300_1941	-	phi77 ORF003-like protein, phage terminase, large subunit	CDS		0.645374	5.30E-03
SAUSA300_1943	-	phi77 ORF040-like protein	CDS		0.878326	2.33E-02
SAUSA300_1975	*lukH*	Aerolysin/leukocidin family protein	CDS	SaeRS	-0.324336	3.93E-02
SAUSA300_1995	*scrR*	sucrose operon repressor	CDS	CcpA	-0.354531	1.92E-02
SAUSA300_2016	*rrlE*	23S ribosomal RNA	rRNA	rRNA	-2.332242	2.18E-05
SAUSA300_2017	*rrsE*	16S ribosomal RNA	rRNA	rRNA	-3.018255	5.08E-06
SAUSA300_2022	*sigB*	RNA polymerase sigma factor SigB	CDS	SigB	-0.246278	3.73E-02

SAUSA300_2023	*rsbW*	serine-protein kinase RsbW	CDS	SigB	-0.245474	2.23E-02
SAUSA300_2056	-	hypothetical protein	CDS	SigB	0.460628	4.56E-02
SAUSA300_2074	*rpmE2*	50S ribosomal protein L31 type B	CDS		0.303758	9.37E-03
SAUSA300_2080	-	hypothetical protein	CDS		0.252262	4.63E-02
SAUSA300_2081	*pyrG*	CTP synthetase	CDS	MgrA	0.379870	1.79E-02
SAUSA300_2097	-	hypothetical protein	CDS	SigB	-0.446302	4.56E-02
SAUSA300_2106	*mtlR*	putative transcriptional regulator	CDS	MtlR	-0.549376	3.13E-03
SAUSA300_2107	*mtlA*	PTS system, mannitol specific IIA component	CDS	MtlR	-0.539293	1.40E-02
SAUSA300_2108	*mtlD*	mannitol-1-phosphate 5-dehydrogenase	CDS	MtlR	-0.445857	8.29E-03
SAUSA300_2123	*rrlF*	23S ribosomal RNA	rRNA	rRNA	-2.335558	2.09E-05
SAUSA300_2124	*rrsF*	16S ribosomal RNA	rRNA	rRNA	-3.066793	3.48E-06
SAUSA300_2142	*asp23*	alkaline shock protein 23	CDS	SigB	-0.534359	7.04E-04
SAUSA300_2144	*amaP*	hypothetical protein	CDS	SigB	-0.548346	3.36E-04
SAUSA300_2164	-	hypothetical protein	CDS	SigB	-1.579792	5.00E-03
SAUSA300_2171	*rpsI*	30S ribosomal protein S9	CDS	L13 leader	0.482163	1.63E-02
SAUSA300_2172	*rplM*	50S ribosomal protein L13	CDS	L13 leader	0.478093	5.94E-03
SAUSA300_2219	*moaA*	molybdenum cofactor biosynthesis protein A	CDS	MgrA	-0.267387	2.29E-02
SAUSA300_2223	*mobB*	molybdopterin-guanine dinucleotide biosynthesis protein B	CDS	MgrA	-0.256143	2.90E-02
SAUSA300_2224	*moeA*	molybdopterin biosynthesis protein A	CDS	MgrA	-0.280256	1.18E-02
SAUSA300_2235	*fhuD2*	iron compound ABC transporter iron compound-binding protein	CDS	Fur	0.321983	2.21E-02
SAUSA300_2261	-	hypothetical protein	CDS	CcpA	-0.337063	1.71E-02
SAUSA300_2286	-	hypothetical protein	CDS		0.518715	5.04E-04
SAUSA300_2294	-	hypothetical protein	CDS	GraRS	-0.244185	4.50E-02
SAUSA300_2298	*emrB*	multidrug resistance protein B, drug resistance transporter	CDS		0.320580	3.77E-02
SAUSA300_2300	-	TetR family transcriptional regulator	CDS	CodY	0.271153	4.55E-02
SAUSA300_2327	-	hypothetical protein	CDS	SigB	-0.355997	3.32E-02
SAUSA300_2374	-	ABC transporter ATP-binding/permease	CDS	GraRS	-0.286559	4.51E-02
SAUSA300_2385	*kimA*	hypothetical protein	CDS		0.343237	3.47E-03
SAUSA300_2392	*opuCB*	glycine betaine/carnitine/choline ABC transporter	CDS	GraRS	-0.496729	4.69E-02
SAUSA300_2396	*pnbA*	para-nitrobenzyl esterase	CDS	SigB	-0.460766	1.08E-02
SAUSA300_2417	-	putative transporter	CDS	CodY	0.273718	2.79E-02
SAUSA300_2443	*gntK*	gluconate kinase	CDS	GntR	-0.446052	1.13E-02
SAUSA300_2447	-	hypothetical protein	CDS	SigB	-0.596698	1.20E-03
SAUSA300_2455	*fbp*	putative fructose-1,6-bisphosphatase	CDS	GraRS	-0.344012	9.80E-03

SAUSA300_2468	-	acetyltransferase	CDS		0.523080	3.06E-03
SAUSA300_2477	*cidC*	pyruvate oxidase	CDS	CidR	-0.325630	6.64E-03
SAUSA300_2498	*crtN*	squalene synthase	CDS	SigB	-0.463055	3.73E-03
SAUSA300_2499	*crtM*	squalene desaturase	CDS	SigB	-0.670453	3.25E-03
SAUSA300_2500	*crtQ*	glycosyl transferase	CDS	SigB	-0.580864	4.74E-03
SAUSA300_2501	*crtP*	phytoene dehydrogenase	CDS	SigB	-0.558134	5.83E-03
SAUSA300_2504	*oatA*	acyltransferase	CDS	GraRS	0.310238	9.12E-03
SAUSA300_2505	-	acetyltransferase	CDS		0.440882	3.28E-02
SAUSA300_2517	-	amidohydrolase family protein	CDS	SigB	-0.362247	3.40E-02
SAUSA300_2518	-	hydrolase family protein	CDS	SigB	-0.386245	2.92E-02
SAUSA300_2526	*pyrD*	dihydroorotate dehydrogenase 2	CDS		0.597480	2.89E-04
SAUSA300_2527	-	hypothetical protein	CDS		0.512625	5.02E-03
SAUSA300_2541	*lqo*	malate:quinone oxidoreductase	CDS		0.189536	3.68E-02
SAUSA300_2548	-	hypothetical protein	CDS	SigB	-0.368208	2.15E-02
SAUSA300_2623	*pcp*	pyrrolidone-carboxylate peptidase	CDS		0.413689	3.24E-02
SAUSA300_2631	-	putative N-acetyltransferase	CDS		-0.386144	2.07E-02
SAUSA300_2647	*rnpA*	ribonuclease P	CDS		0.389345	2.25E-02

Table S6. GSEA analysis results.

**Locus**	**Gene symbol**	**Description**	**Enriched pathway in Δ*ofrA***	**Log2FoldChange**	**P-value**
SAUSA300_0358	*metF*	bifunctional homocysteine S-methyltransferase/5,10-methylenetetrahydrofolate reductase protein	One carbon pool by folate	-0.395075	3.40E-02
SAUSA300_0974	*purN*	phosphoribosylglycinamide formyltransferase		-0.378502	7.98E-03
SAUSA300_0975	*purH*	bifunctional phosphoribosylaminoimidazolecarboxamide formyltransferase/IMP cyclohydrolase		-0.386208	4.08E-03
SAUSA300_1678	*fhs*	formate--tetrahydrofolate ligase		-0.384792	1.61E-02
SAUSA300_2498	*crtN*	squalene synthase	Carotenoid biosynthesis	-0.463055	3.73E-03
SAUSA300_2499	*crtM*	squalene desaturase		-0.670453	3.25E-03
SAUSA300_2500	*crtQ*	glycosyl transferase		-0.580864	4.74E-03
SAUSA300_2501	*crtP*	phytoene dehydrogenase		-0.558134	5.83E-03
